# Patient-derived pediatric brain tumor orthotopic xenografts and tumor organoids faithfully recapitulate primary tumors

**DOI:** 10.1126/sciadv.aea4966

**Published:** 2026-04-15

**Authors:** Justin S. Williams, Dana M. Farmer, Qianqian Li, Jake D. Friske, Jose Grenet, Sarah Robinson, Kimberly S. Mercer, Vanshita Goel, Laura Janke, Liusheng He, Paul Klimo, Jason C.-H. Chiang, Giles W. Robinson, Brent A. Orr, Martine F. Roussel

**Affiliations:** ^1^ Department of Tumor Cell Biology, St. Jude Children’s Research Hospital, Memphis, TN, USA.; ^2^ Comparative Pathology Core, St. Jude Children’s Research Hospital, Memphis, TN, USA.; ^3^ Department of Host Microbe Interactions, St. Jude Children’s Research Hospital, Memphis, TN, USA.; ^4^ Department of Surgery, Le Bonheur Children’s Hospital, Memphis, TN, USA.; ^5^ Department of Pathology, St. Jude Children’s Research Hospital, Memphis, TN, USA.; ^6^ Department of Neuro-Oncology, St. Jude Children’s Research Hospital, Memphis, TN, USA.

## Abstract

Extensive molecular analyses by many groups have revealed a heterogenous landscape of embryonal brain tumors, but patient-derived tumor organoid (TO) model development has remained limited. Here, we describe the establishment of TO and TO xenografts (TOX) from patient-derived orthotopic xenografts (PDOXs) of medulloblastoma, embryonal tumor with multilayer rosettes, and atypical teratoid rhabdoid tumors. DNA methylation, bulk- and single-cell RNA sequencing, and whole-genome sequencing demonstrated that TOs and TOXs faithfully recapitulate the epigenetic, transcriptomic, and genetic landscape of PDOXs, as well as replicate the intratumor cellular heterogeneity of PDOXs that are often lost in established cell lines. We show that TOs and PDOXs have similar drug responses. The development of embryonal brain TOs will facilitate in vitro functional assays, including high-throughput drug or CRISPR screens, without the need for fresh tumors from tumor-bearing mice. This will accelerate the identification and validation of vulnerabilities and therapeutic strategies for preclinical testing toward clinical trials.

## Teaser

Tumor organoids from embryonal brain tumors can be used for functional and translational assays to accelerate clinical trials.

## INTRODUCTION

Pediatric brain and central nervous system (CNS) tumors are a leading cause of disease-related deaths in the United States annually (*1*). Childhood mortality due to primary malignant brain tumors is disproportionate among other cancers: high-grade gliomas (HGGs), ependymomas (EPNs), atypical teratoid rhabdoid tumors (ATRTs), medulloblastomas (MBs), and other embryonal brain tumors such as embryonal tumor with multilayer rosettes (ETMRs) accounted for ~75% of deaths from 2014–2018 (*2*). An increased understanding of the underlying biology of these diseases through genomic and epigenetic analyses (*3*) has guided molecularly driven clinical trials, including SJDAWN (NCT03434262), SJELIOT (NCT04023669), and SJMB12 (NCT01878617). While these trials are encouraging advances in the field, more is needed to improve treatment and quality-of-life outcomes. Therapeutic intervention consists of surgical resection, when possible, followed by radiotherapy for children older than 3, and drug combination with cytotoxic chemotherapy including vincristine and etoposide (*4*, *5*). Because of the marginal effectiveness of chemotherapy for the treatment of pediatric HGG and EPN, its use has been limited to pediatric embryonal tumors (MB, ATRT, and ETMR). Survival outcomes are variable among the different tumor entities, with MB and EPN being the most responsive to therapeutic intervention (*6*). Still, survivors continue to suffer from treatment-associated morbidity, including neurocognitive, neuroendocrine, and psychological deficits (*7*). These life-long afflictions compromise survivors’ quality of life and highlight the urgent need to identify more effective therapies that reduce treatment-related complications and unwanted lasting effects.

Advances in genetically engineered mouse models (GEMMs) have considerably advanced our knowledge of tumor biology and have provided a mechanism for evaluating therapeutic strategies in a syngeneic, immune-competent environment (*8*–*10*). While these models have been beneficial, they fail to recapitulate the heterogenous molecular landscapes that are observed in human tumors (*11*). For example, most GEMMs of group 3 MB (MB-G3) are generated by overexpression of *Myc* with *Trp53* loss or *Myc* with *Gfi1* or *Gfi1B* overexpression in neuronal progenitors followed by orthotopic implant in the cerebella or cortices of naïve recipient mice (*10*, *12*). GEMMs of Sonic hedgehog MB (MB-SHH) that rely on the deletion of *Ptch1* with or without *Trp53* loss or enforced expression of *Mycn* and loss of *Trp53* in Atoh1-positive granule neuron progenitors (GNP) populations, lack mutations of other SHH drivers, such as *Sufu*, *Smo*, and *Gli2*, commonly observed in patient populations (*13*, *14*). Failure to recapitulate the inter and intratumoral heterogeneity of human tumors in GEMMs has resulted in few translational findings and acts as a barrier to improved clinical outcomes.

To recapitulate the diversity of the pediatric CNS tumor landscape, patient-derived orthotopic xenografts (PDOXs) (*15*–*17*) of childhood brain tumors have recently emerged as an essential resource for understanding disease biology and investigating and testing more effective therapies. At St. Jude Children’s Research Hospital (SJCRH), over a period of 12 years (September 2012 to December 2024), we have established, characterized, and maintained 81 PDOX models representing a diverse range of pediatric brain tumors, among which 39 were previously published (*17*). These models have been used to identify novel targeted therapies, some of which have been translated into clinical trials for children with primary or recurrent/progressive malignant brain tumors (*18*–*21*). Even with these models, bench-to-bedside translations have remained limited, in part because most PDOXs must be propagated from mouse to mouse and grow ex vivo only transiently, which limits the use of high-throughput assays or other functional manipulations. This hindered their impact for functional studies and limits their utilization to in vivo preclinical studies (*20*). To address these limitations, the field has turned to the development and implementation of organoid technology for establishing and expanding three-dimensional (3D) cell cultures, which efficiently recapitulate human disease (*22*). We and others found that organoids derived from tumor tissue [referred to as tumor organoids (TOs) hereafter] preserve many tumor characteristics, including genetic and transcriptomic profiles, even after long-term passaging in 3D cultures (*22*, *23*). While the tumor microenvironment (TME) is not conserved because of the loss of immune cells, TOs effectively predict patient-specific drug responses, highlighting their potential for identifying therapeutic regimens (*24*–*26*). Since there is still a dearth of TO models for malignant pediatric brain tumors (*23*, *27*, *28*), we developed a pipeline for establishing and characterizing patient-derived TO models of pediatric CNS tumors. Using a combination of genetic, epigenetic, and transcriptomic techniques, we demonstrate that these TOs recapitulate the molecular characteristics of the tumors from which they are derived. We also found that several TOs grew in mice after reimplantation [TO xenograft (TOX)] that mimic PDOXs and primary tumors, broadening the range of preclinical studies to support clinical trials.

## RESULTS

### Establishment of PDOXs

PDOXs were generated from primary tumor samples obtained by surgical resection or autopsy from primary or recurrent malignant CNS tumors from SJCRH and the Children’s Oncology Group (COG) (Table 1). Primary tumors that successfully engrafted in NSG mice were passaged in CD-1 nude mice for no more than three passages without any intermediate culture steps to preserve the phenotypic and molecular characteristics of the primary patient tumors as outlined in our previous manuscript (*17*). We switched from NSG to CD-1 nude mice due to NSG mice being more prone to infection than CD1-nude. Because preclinical trials sometimes take up to 6 months, we used CD-1 nude mice for translational studies over NSGs. We did not observe growth rate differences between NSG and CD-1 nude mice once PDOX models were established, in this study and as previously described (*17*).

**Table 1.** Phenotypic and molecular annotations of 46 newly established PDOX embryonal brain tumor models. Wingless (WNT), Sonic Hedgehog (SHH), group 3 (G3), group 4 (G4), posterior fossa EPN group A (EPN PFA), atypical teratoid rhabdoid tumor (ATRT), medullomyoblastoma (MMB), neuroblastoma (NBL); NP, not provided; NA, not analyzed; OE, overexpression; amp, amplification; pred, predicted. PDOX1 and PDOX2: from the same patient.

Model ID	Age	Sex (M/F)	Source	Classification	Genotype	PDOX tumor latency (months)
**Medulloblastoma**						
SJMB030748_PDOX	16	F	Primary	MB WNT	*CREBBP*, *CTNNB1*, 6q loss	4
SJMB075701_PDOX	27	M	Primary	MB WNT	*CTNNB1*, *PTCH1*, *PMS2*	9
SJMB080134_PDOX	5	M	Primary	MB WNT	*SMARCA4*, 6 monosomy	4
SJMMB031023_PDOX	21	F	Primary	MMB	*CTNNB1*	7
SJMB031645_PDOX	6	F	Primary	MB SHH-3	*PTCH1*, *PTEN*	4
SJMB031813_PDOX	8	M	NA	MB SHH-3	*GLI2* gain/rearranged	3
SJMB031418_PDOX1	1	M	Relapse	MB SHH-1	*PTCH1*, *TP53*, i17q	3
SJMB031476_PDOX	2	F	Primary	MB SHH-1	*PIK3CA*	2
SJMB031418_PDOX3	3	M	Autopsy	MB SHH-1	*PTCH1*, *TP53*	4
SJMB031418_PDOX2	3	M	Autopsy	MB SHH-1	*PTCH1*, *TP53*	2
SJMB032603_PDOX1	16	F	Primary	MB SHH-4	*GLI2* amp, *CCND1* gain/rearranged	4
SJMB032334_PDOX	3	M	Relapse	MB SHH-1	*MYCN* amp	9
SJMB033384_PDOX	5	M	Primary	MB SHH-2	*PTCH1*, *CCND2* amp, *TERT* amp, *SETBP1*	8
SJMB032603_PDOX2	11	F	Relapse	MB SHH-4	*GLI2* amp, *CCND1* gain/rearranged	2
SJMB093837_PDOX	17	F	Primary	MB SHH-4	*SUFU*	9
SJMB036222_PDOX	4	M	Primary	MB SHH-3	*MYCN* amp, *GLI2* amp, *TP53*	2
SJMB032268_PDOX1	5	F	Primary	MB–G3/G4-4	3, 4, 8, 9, 10, 15q, 16q, 20, 21 loss	8
SJMB032268_PDOX2	5	F	Primary	MB–G3/G4-4	3, 4, 8, 9, 10, 15q, 16q, 20, 21 loss	7
SJMB032425_PDOX	2	M	Primary	MB–G3/G4-2	*MYC* amp	4
SJMB073562_PDOX	2	F	Primary	MB–G3/G4-2	*MYC* amp, *TP53*, i17q	2
SJMB033373_PDOX	5	M	Primary	MB–G3/G4-2	*MYC* amp, *CTDNEP1*, i17q	1
SJMB034878_PDOX	10	M	Primary	MB–G3/G4-1	*MYCN* amp	6
SJMB031757_PDOX	16	M	Primary	MB–G3/G4-8	*PRDM6*/*SNCAIP* gain, i17q	12
SJMB031857_PDOX	11	M	Primary	MB–G3/G4-8	*SUPT16H*/*METTL3* gain, i17q	9
**Embryonal tumor with multilayer rosettes**						
SJETMR031447_PDOX	3	M	Primary	ETMR C19MC-altered	*CTNNB1*, *TTYH1*-*C19MC*	3
SJETMR085464_PDOX	2	M	Primary	ETMR C19MC-altered	*TTYH1* − *C19MC*	4
**Atypical teratoid rhabdoid tumor**						
SJATRT030443_PDOX	2	M	NA	ATRT MYC	*SMARCB1*	10
SJATRT031880_PDOX	4	F	Primary	ATRT MYC	*SMARCB1*, 2 gain	2
SJATRT034656_PDOX	2	F	Autopsy	ATRT MYC	*SMARCB1*	1
SJATRT034110_PDOX	1	M	Autopsy	ATRT MYC	*SMARCB1*	3
SJATRT032346_PDOX	4	F	Primary	ATRT SHH	*SMARCB1*, 8 gain	2
SJATRT096759_PDOX1	NA	NA	Autopsy	ATRT SHH	*SMARCB1*	3
SJATRT096759_PDOX2	NA	NA	Autopsy	ATRT SHH	*SMARCB1*	3
**Ependymoma**						
SJEPD030791_PDOX	14	M	Relapse	EPN ZFTA fusion-e	*ZFTA*-*RELA*, *CDKN2A*/*B*	6
SJEPD030795_PDOX	9	F	Relapse	EPN PFA1	*EZHIP* OE	6
SJEPD031102_PDOX	6	F	Relapse	EPN ZFTA fusion-d	*ZFTA*-*RELA*	4
SJEPD030399_PDOX	7	M	Relapse	EPN PFA2	*EZHIP* OE	11
SJEPD031150_PDOX	5	M	Autopsy	ZFTA-RELA	*ZFTA*-*RELA*, *CDKN2A*/*B*	8
SJEPD031786_PDOX	4	M	Relapse	EPN PFA1	*EZHIP* OE	2
SJEPD032263_PDOX	2	M	Primary	EPN PFA1	*EZHIP* OE	3
SJEPD055862_PDOX	7	M	Relapse	EPN PFA1	*EZHIP* OE	6
**Other**						
SJSAR032239_PDOX	10	M	Primary	Sar CIC-rearranged	*PRDM5*-*PRR12*	1
**COG**						
COGEPD059007_PDOX	15	M(pred)	Primary	EPN PFA-1	*EZHIP* OE	9
COGEPD093838_PDOX	16	NA	Primary	EPN ZFTA fusion-a	NA	8
COGMB055618_PDOX	NA	F	Primary	MB SHH-4	*SUFU*, *CCND2* amp, *TP53*	5
COGNBL055619_PDOX	2	F(pred)	Metastatic	NBL FOXR2	*FOXR2* gain	4

From 2018 to 2024, we established and amplified 42/46 PDOXs from 136 implanted primary tumors from SJCRH, representing a diverse range of malignant childhood CNS tumor types (fig. S1). We generated seven ATRTs (four ATRT-MYC and three ATRT-SHH), eight EPNs [five EPN–posterior fossa (PFA) and three EPN-ZFTA fusion], and two ETMRs, both with C19MC locus amplification and rearrangement (Table 1). We developed 25 MB PDOXs, representing each of the four molecular subgroups: Wingless (MB-WNT, *n* = 3), MB-SHH (*n* = 12), MB-G3 (*n* = 6), and MB-G4 (*n* = 2), and a rare variant of MB, medullomyoblastoma (MMB, *n* = 1). Only one *CIC*-rearranged sarcoma (SAR) engrafted in the mouse brains. PDOX tumor latencies varied from 1 to 12 months (Table 1). As previously reported, PDOXs retained tumor latencies over multiple passages from mouse to mouse (*17*). If implants were generated from cryopreserved tumors, latency increased by 1 to 2 months. A retrospective analysis of all implanted tumors from 2018 to 2023 indicated a significant difference in the median of viable tumor cells implanted between failed and successful engraftment (failed: 2.7 × 10^5^ tumor cells, successful: 7.2 × 10^5^ tumor cells, *P* value: 1.65 × 10^−4^) (fig. S1, A to C). Subsequent implants from 2023 to 2024 with 1.5 to 2.0 × 10^6^ viable tumor cells per mouse resulted in an increase in the engraftment rate from 28% (35 of 124) to 58% (7 to 12) (fig. S1D).

From 2016 to 2024, we successfully developed 4/46 PDOXs from 55 primary COG tumors (Table 1). These included two EPNs: one EPN-PFA and one EPN-ZFTA, one MB-SHH, and one CNS neuroblastoma, FOXR2-activated (CNS-NB-FOXR2). The lower efficiency was attributed to challenges related to tumor viability and the limited number of tumor cells available for implantation.

### Molecular characterization of pediatric brain tumor PDOXs

**Fig. 1.**
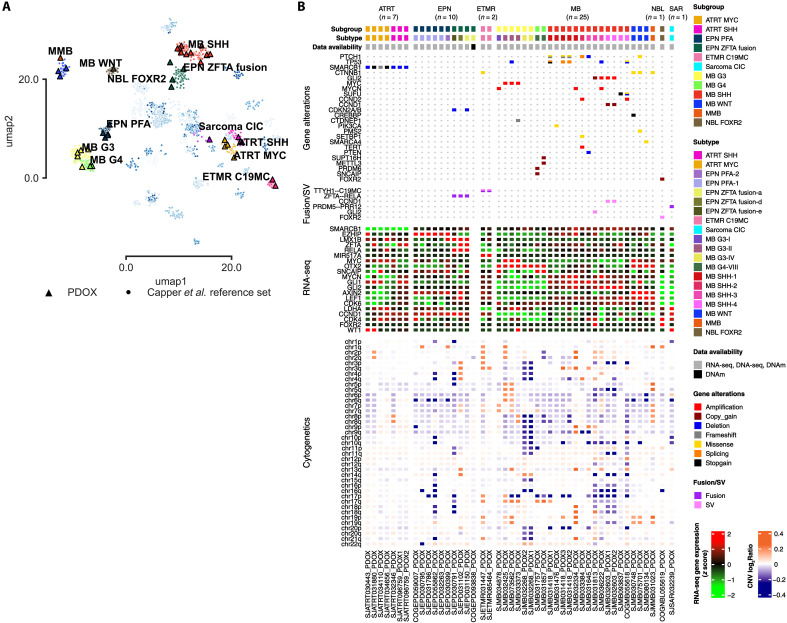
Molecular analysis of 46 established pediatric CNS PDOX models. (**A**) UMAP projection of established pediatric CNS tumor PDOX models (triangles) overlaid on the Capper *et al.* (*29*) brain tumor methylation dataset (dots). (**B**) Oncoprint of PDOX models emphasizing gene alterations including mutations, amplifications (defined as estimated copy number ≥ 5), deletions, structural variants, RNA fusions, cytogenetic alterations, and previously reported cancer entity–specific RNA-seq gene expression. Chromosomal copy variation is reported as the log ratio of DNAm profiles in comparison to the control tissue reference set. The mouse passage at analysis of each model is listed in table S1.

Molecular characterization of each PDOX tumor was performed by EPIC DNA methylation (DNAm) array profiling, DNA sequencing [whole-genome (WGS) and whole-exome (WES)], and bulk RNA sequencing (RNA-seq). PDOX models were first classified by DNAm array using the MNP classifier v12 (*29*) (table S1) and confirmed by Uniform Manifold Approximation and Projection (UMAP) analysis (Fig. 1A) and unsupervised hierarchical clustering of the topmost variably methylated probes (fig. S2). Transcriptomic profiles were compared by unsupervised hierarchical clustering of the 1000 topmost variably expressed genes (fig. S3). Characterization of the molecular alterations of each PDOX was performed using WGS/WES with paired patient germline samples when available (*n* = 28 of 42; 67%) (Fig. 1B). Known or candidate driver genes and molecular alterations were identified for the majority of established PDOX (*n* = 43 of 46; 93%). Models with paired source tumor DNA sequencing (*n* = 17 of 42; 40%) showed maintenance of genetic alterations in 16 of 17 PDOXs (fig. S4 and tables S2 to S4). We confirmed the molecular fidelity of transcriptomic profiles between PDOX and matching source tumor in 22 of 42 tumors by unsupervised hierarchical clustering of the pairwise Pearson correlation distances (fig. S5).

### Atypical teratoid rhabdoid tumors

All seven ATRT PDOXs contained biallelic loss of *SMARCB1* (Fig. 1B). PDOX model SJATRT032346 displayed an RNA-seq profile inconsistent to that of the other ATRT-SHH models (fig. S3), and copy gain of chromosome 8 (Fig. 1B). Classification by DNAm profile analysis for this model demonstrated confident scoring for ATRT-SHH (≥0.90) by the MNP classifier (table S1) and ATRT-specific clustering (Fig. 1A and fig. S2). *MYC* gene expression in this model was higher than the other classified ATRT-SHH PDOX models, consistent with *MYC* copy gain (fig. S3), providing a likely explanation of the inconsistencies between DNAm and RNA-seq profile analyses.

### Ependymoma

Of the 10 EPN PDOXs, 6 were EPN-PFA (PFA1, *n* = 5; PFA2, *n* = 1) with few notable gene alterations, except for overexpression of *EZHIP* at the RNA-seq level (Fig. 1B). PDOX classified as EPN-ZFTA with available RNA-seq data revealed ZFTA-RELA fusions (*n* = 3 of 3) (table S4 and fig. S6) and overexpression of *LMX1B* (Fig. 1B). PDOX SJEPD031102 (EPN-ZFTA) showed differences in transcriptomic profiles to other classified EPN-ZFTA fusion PDOX samples (fig. S3); it harbored gain of chromosomes 3, 4, 13q, and 14q, in contrast to the copy-loss or copy-neutral status of these chromosomes in the other EPN-ZFTA PDOX (Fig. 1B).

### Embryonal tumor with multilayer rosettes

Two ETMR PDOXs engrafted in mice, both of which showed partial chromosome 19 duplication and rearrangement, resulting in the defining fusion of *TTYH1* with the *C19MC* microRNA cluster (figs. S3 and S7) (*30*). PDOX SJETMR031447 also contained a *CTNNB1* hotspot mutation (Fig. 1B), previously identified in a small subset of *C19MC*-rearranged ETMRs (*31*).

### Medulloblastoma

Twenty five MB PDOXs were established, consisting of 8 MB-G3/G4 (G3/G4-1, *n* = 1; G3/G4-2, *n* = 3; G3/G4-4, *n* = 2 G3/G4-8, *n* = 2), 13 MB-SHH (SHH-1, *n* = 5; SHH-2, *n* = 1; SHH-3, *n* = 3; SHH-4, *n* = 4), 3 MB-WNT, and 1 MMB. *CTNNB1* hotspot mutations were found in two of three MB-WNT and the MMB PDOXs. One MB-WNT contained monosomy 6 (SJMB080134_PDOX), while SJMB030748 had chromosome 6q copy-loss. MB-WNT SJMB080134 did not have a canonical hotspot *CTNNB1* mutation, nor did we find other frequently reported driver gene mutations (*11*) but carried a heterozygous missense mutation in *SMARCA4* [p.D1183H; variant allele frequency (VAF) = 0.48; Rare Exome Variant Ensemble Learner (REVEL) = 0.89], previously reported to have a dominant-negative effect for inactivating mutations in the adenosine triphosphatase domain (table S2) (*32*).

Five MB-SHH had inactivating *TP53* alterations in addition to *SUFU* and *CCND2* amplification (*n* = 1), *MYCN* and *GLI2* amplification (*n* = 1), and *PTCH1* alterations (*n* = 3) (Fig. 1B). MB-SHH SJMB033384 presented with several alterations, including *PTCH1* splicing, *CCND2* amplification, *TERT* amplification, and a *SETBP1* missense mutation. MB-SHH SJMB031645 deleted *PTCH1* and *PTEN*. Two MB-SHH PDOX from the same patient (SJMB032603_PDOX1 and SJMB032603_PDOX2) amplified *GLI2* with rearrangement of chromosome 11q, including *CCND1* (Fig. 1B and fig. S8). Similarly, MB-SHH SJMB031813 had a chromosome 2 rearrangement resulting in *GLI2* copy gain (fig. S9). A single gene alteration was identified in three MB-SHH PDOXs: SJMB032334 with *MYCN* amplification, SJMB093837 with a nonsense mutation in *SUFU*, and SJMB031476 with a hotspot missense mutation in *PIK3CA* (Fig. 1B and table S2).

Three MB-G3/G4 models amplified *MYC* in addition to *TP53* deletion (*n* = 1) and *CTDNEP1* frameshift (*n* = 1). *MYC* amplification was identified in SJMB034878, classified as MB–G3/G4-1. Both MB–G3/G4-8, SJMB031757 and SJMB031857, had small focal tandem duplications of *PRDM6*/*SNCAIP* and *SUPT16H*/*METTL3*, respectively (Fig. 1B and table S3). Two MB–G3/G3–4 PDOXs established from the same patient, SJMB032268_PDOX1 and SJMB032268_PDOX2, showed no evidence of any hallmark genetic alterations, although both display broad genomic copy loss (Fig. 1B).

### Other tumor types

A neuroblastoma PDOX COGNBL055619 contained a partial rearrangement of chromosome X, resulting in *FOXR2* copy gain by tandem duplication and *FOXR2* overexpression (Fig. 1B). We found no genetic driver alterations in the sarcoma PDOX SJSAR032239, although we identified a chr4-chr19 translocation typical of *CIC*-rearranged sarcomas (fig. S10A). SJSAR032239 had a previously unreported in-frame RNA fusion of *PRDM5*, a transcription factor, with *PRR12*, a proline-rich DNA binding protein with uncharacterized function (fig. S10B and table S4).

#### Pipeline for establishing TOs

**Fig. 2.**
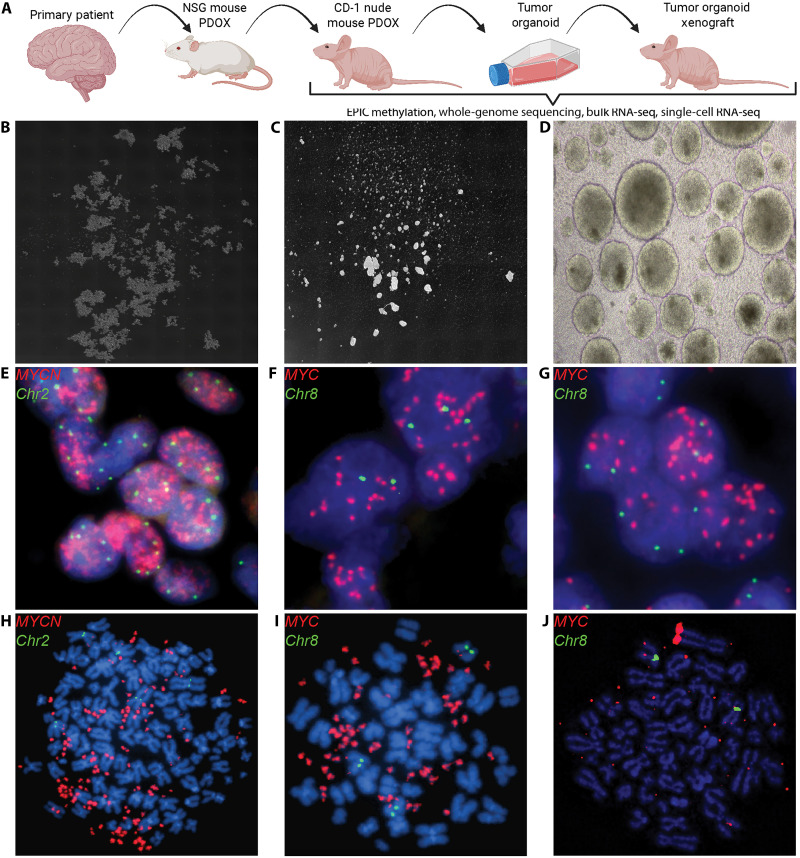
Establishment of TO models. (**A**) Schematic of the development pipeline to establish and molecularly characterize TO and TOX models. (**B** and **C**) Representative images of TO models (4× magnification) growing as grape-like clusters MB-SHH SJMB016874 (B), and as spheroids MB-G3 SJMB030315 (C). (**D**) Representative image of the TO model SJETMR031447 with dark centers ready for passaging (10× magnification). (**E** to **J**) FISH images of primary patient tumors, MB-SHH SJMB016876 (E), MB- G3 SJMB030315 (F), and MB-G3 SJMB032425 (G) showed *MYCN* and *MYC* amplification, respectively. (H) to (J) Highlight maintenance of *MYCN* and *MYC* amplification in TOs in the form of double minutes and homologous staining regions for the same tumors as in (E) to (J).

We developed a stepwise pipeline to generate TOs from PDOXs (Fig. 2A). Tumor cells dissociated from PDOXs were seeded in 96-, 24-, or 6-well plates, on the basis of the number of tumor cells available, in Tumor Stem Medium (TSM) full or EFab media and expanded as spheroids in vitro (Table 2, table S8, and Materials and Methods). We observed a latency period between the initial passage and TO establishment (fig. S11). At each passage during the establishment period, TOs were counted, reseeded, and allowed to expand. The doubling time was calculated on the basis of the number of cells seeded compared to the final live cell count at each passage (Materials and Methods). A sliding window of three consecutive doubling times was used to calculate the SD as a measurement of consistency. When this measure was less than or equal to 20% of the maximum doubling time for a particular TO (with a minimum threshold of 1 day), a TO was considered “established.” For the TOs presented in fig. S11, this threshold of 20% of the maximum doubling time was 2.3, 1.6, 3.3, and 1.0 days and were considered established at days 75, 68, 86, and 49 for MB-SHH SJMB016874, MB-G3 SJMB016880, MB-G3 SJMB030315, and ATRT-MYC SJATRT034110, respectively. TOs derived from PDOX tumors took an average of 75.9 days from initial seeding to establishment (*n* = 9, minimum = 37, maximum = 155, range = 118) (Table 2). There is an example where the TO culture MB-G3 SJMB033373 contained mostly mouse cells, preventing its molecular characterization. Mouse cell depletion before initial seeding allowed the establishment of the TO model and downstream molecular analysis. We subsequently used mouse cell depletion before initial seeding of PDOX cells for TO establishment and observed a significant decrease in the amount of time (in days) until the cells were ready for the first passage (*P* = 0.0476) (fig. S12).

**Table 2.** List of TO attempted and engrafted as TOX. Tumor cells from PDOXs cryopreserved (cryovial) or from tumors isolated from the brain of tumor-bearing animals (fresh) at moribund age were grown in 3D cultures. Doubling time is the average doubling time for three consecutive passages provided with the standard deviation (SD) for the three consecutive passages. MB-SHH SJMB032603 and SJMB032603_2 are samples from the same patient. PDOX, patient-derived orthotopic xenografts; TOX, TO xenograft; Amp, amplification; mut, mutation; N/A, not applicable; i17q, isochromosome 17q; TBD, to be done.

Model	Genotype	PDOX	Tumor organoid	Source PDOX	Growth media	Doubling time (days)	Time to establishment (days)	T.O.X. latency (weeks)
**Medulloblastoma**								
**WNT**								
SJMB041804	*CTNNB1*, *MLL3*	Yes	Failed	N/A	N/A	N/A	N/A	N/A
SJMB030748	*CTNNB1*, *CREBBP*	Yes	Failed	N/A	N/A	N/A	N/A	N/A
SJMB080134	*SMARCA4*, monosomy chr 6	Yes	Failed	N/A	N/A	N/A	N/A	N/A
**SHH**								
SJMB016874	*MYCN* amp, *TP53*	Yes	Yes	Cryovial	TSM full	5.2 ± 0.8	75	7
SJMB016876	*CDK4* amp, *MYCN* amp, *TP53*, i17q	Yes	Yes	Cryovial	TSM full	2.7 ± 1.1	51	3.5
SJMB044820	*CDK4* gain, *MYCN* amp, i17q	Yes	Failed	N/A	N/A	N/A	N/A	N/A
SJMB041807	*TP53*, *PTCH1*, i17q	Yes	In progress	Fresh	N/A	TBD	TBD	TBD
SJMB055853	*GLI2* amp, *TP53*	Yes	Failed	Cryovial	N/A	N/A	N/A	N/A
SJMB031813	*GLI2* gain/rearranged	Yes	In progress	Fresh	N/A	TBD	TBD	TBD
SJMB032603	*GLI2* amp, *CCND1* gain/rearranged	Yes	Failed	Fresh	N/A	N/A	N/A	N/A
SJMB032603_2	*GLI2* amp, *CCND1* gain/rearranged	Yes	Failed	Fresh	N/A	N/A	N/A	N/A
SJMB093837	*SUFU*	Yes	Failed	Fresh	N/A	N/A	N/A	N/A
SJMB036222	*MYCN* amp, *GLI2* amp, *TP53*	Yes	Yes	Fresh	TSM full	8.7 ± 0.7	130	TBD
**Group 3**								
SJMB016880	*MYC* amp, *i17q*	Yes	Yes	Cryovial	TSM full	2.6 ± 0.2	68	10
SJMB030315	*MYC amp*, *CTDNEP1, i17q*	Yes	Yes	Cryovial	TSM full	4.8 ± 1.1	86	12
SJMB032425	*MYC* amp	Yes	Yes	Fresh	TSM full	5.4 ± 0.9	37	TBD
SJMB033373	*MYC* amp, *CTDNEP1*, *i17q*	Yes	Yes	Cryovial	TSM full	3.8 ± 1.9	64	2.5
**Group 4**								
SJMB058997	*MYCN* amp, *PRDM6*	Yes	Failed	N/A	N/A	N/A	N/A	N/A
SJMB054606	*MYCN* amp, *MUC16*	Yes	In progress	Fresh	N/A	TBD	TBD	TBD
**Embryonal tumor with multilayer rosettes**								
SJETMR031447	*CTNNB1*, *TTYH1 − C19MC*	Yes	Yes	Fresh	EFab	12.6 ± 2.3	44	TBD
SJETMR085464	*TTYH1 − C19MC*	Yes	Failed	Fresh	N/A	N/A	N/A	N/A
**Atypical teratoid rhabdoid tumor**								
**SHH**								
SJATRT041800 [AT-SHH02 (*23*)]	*SMARCB1*, *CDKN2A/B*, *TSC1*	Yes	Yes	Cryovial	TSM full	2	(*23*)	2.5
SJATRT032047 [AT-SHH18 (*23*)]	*SMARCB1*	Yes	Yes	Cryovial	TSM full	4.5	(*23*)	Failed
SJATRT032417 [AT-SHH17 (*23*)]	*SMARCB1*	Yes	Yes	Cryovial	TSM full	2.8	(*23*)	Failed
SJATRT096759	*SMARCB1*	Yes	In progress	Fresh	N/A	TBD	TBD	TBD
**MYC**								
SJATRT034656	*SMARCB1*	Yes	Yes	Fresh	TSM full	5.4 ± 1.5	155	N/A
SJATRT034110	*SMARCB1*	Yes	Yes	Fresh	TSM full	3.2 ± 0.2	49	3
SJATRT059003 [AT-MYC07 (*23*)]	*SMARCB1*, *NIPBL*, *ABL1*	Yes	Yes	Cryovial	TSM full	2.2	(*23*)	Failed
SJATRT059003_2 [AT-MYC08 (*23*)]	*SMARCB1*, *NIPBL*, *ABL1*	Yes	Yes	Cryovial	TSM full	3.3	(*23*)	Failed
SJATRT030997	*SMARCB1*	Yes	Yes	PDOX cryovial	TSM full	2.5	(*23*)	12 (NSG)

TO cultures were attempted from 24 established PDOXs, including 19 MB (WNT, *n* = 3; SHH, *n* = 10; G3, *n* = 4; G4, *n* = 2), 2 ETMR, and 3 ATRT (Table 2 and fig. S13). Ten PDOXs were established as TOs: three *MYCN*-amplified/*TP53*-mutated MB-SHH, four *MYC*-amplified MB-G3, two ATRT-MYC, and one ETMR (Table 2 and fig. S13). We found that fibroblast growth factor 2 (FGF2) and platelet-derived growth factor AA (PDGF-AA) were required for MB-SHH TO while FGF2 and epidermal growth factor (EGF) alone provided the best proliferation of MB-G3 TO without the requirement for PDGF-AA or PDGF-BB (fig. S14 and table S5). As previously reported, ATRT required PDFG-AA and PDGF-BB in addition to EGF and FGF2 (*23*). To understand why different media formulations affected TO proliferation between MB-SHH and MB-G3, we analyzed the expression of relevant growth factor receptors (fig. S15) (*33*). PDGF receptor expression was detected in MB-SHHs, supporting the need for PDGF-AA to promote proliferation in vitro. FGFR3 and FGFR4 were highly expressed in MB-G3 subtype 2 (II), hallmarked by *MYC* amplification representing the subgroup/subtype classification of all established MB-G3 TOs described here. This reinforces the finding that FGF2 was necessary to promote proliferation in these TOs, the absence of which led to poorer growth kinetics (table S5 and fig. S14). TOs grew in suspension as cell clusters or spheroids. Some TO models grew as grape-like clusters (Fig. 2B) with irregular borders and asymmetry, while others grew as rounded spheroids with circular borders and minimal clumping between clusters (Fig. 2C). TOs were ready to passage when they were 325 to 650 μm across (spheroids) or 750 to 1250 μm across (grape-like clusters) and had formed dark centers with the accumulation of loose single-cells surrounding the large clusters (Fig. 2D and fig. S16).

Fluorescence in situ hybridization (FISH) imaging of MB-SHH and MB-G3 primary patient tumors showed the presence of *MYCN* or *MYC* amplification, respectively (Fig. 2, E to G) (*17*). Similar staining of the TOs developed from each model showed consistent amplification of *MYCN* or *MYC* in the form of extrachromosomal DNA (ecDNA) as double minutes (Fig. 2, H and G) or homogeneous staining regions (Fig. 2J and fig. S17). The establishment of TOs from non-*MYC* or non-*MYCN* amplified tumors was unsuccessful using the same or previously reported media (*27*), likely due to other growth factor or TME requirements for proliferation.

#### Preservation of the transcriptomic, epigenetic, and genetic landscape of PDOX, TO, and TOX trios

**Fig. 3.**
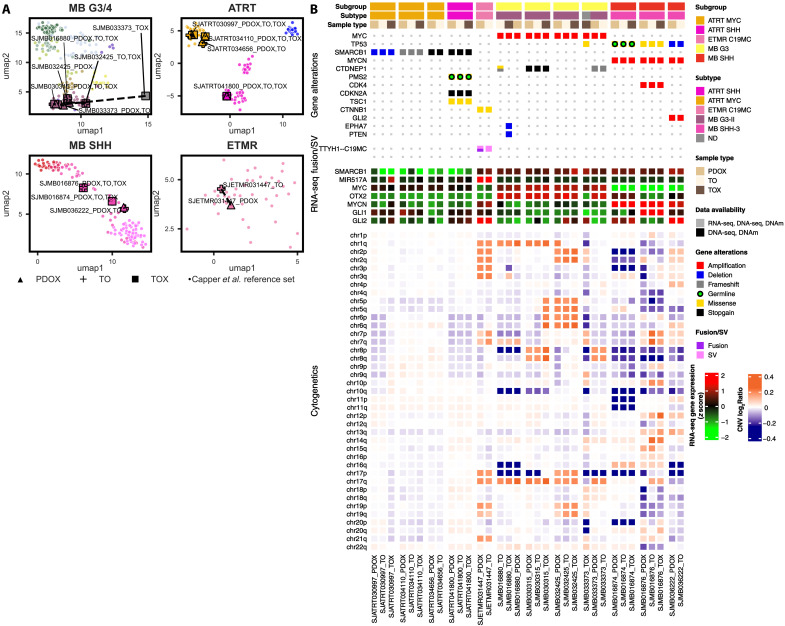
Bulk molecular analysis of PDOX, TO, and TOX datasets. (**A**) UMAP projection of the newly developed PDOX (triangle), TO (cross), and TOX (square) overlaid on the Capper *et al.* (*29*) brain tumor DNAm array dataset (dots). (**B**) Oncoprint of TO, TOX, and matching PDOXs emphasizing gene alterations including mutations, amplifications (defined as estimated copy number ≥ 5), deletions, structural variants, RNA fusions, cytogenetic alterations, and previously reported cancer entity–specific RNA-seq gene expression. Chromosomal copy variation is reported as the log ratio of DNAm profiles in comparison to the Capper *et al.* (*29*) control tissue reference set.

Molecular characterizations and comparisons of each PDOX, TO, and TOX trio were performed using the same approach as for the PDOXs. Each dataset was first classified by the MNP v12 classifier and UMAP analysis (Fig. 3A) in comparison to the Capper *et al.* reference set (*29*). We found consistent clustering by tumor type and annotated subgroups for all samples within PDOX/TO/TOX datasets except for MB-G3 SJMB033373 (Fig. 3A). We identified consistent unsupervised clustering using the topmost variable DNAm probes (fig. S18) and conservation of gene alterations, chromosomal amplifications, and deletions (Fig. 3B and fig. S19). While *MYC* amplification was maintained in MB-G3 SJMB033373, structural variation of this amplification changed from the PDOX profile (fig. S20).

Consistency was also seen in the transcriptomic profiles of trio datasets using an unsupervised clustering analysis of the topmost variably expressed genes (fig. S21). Genetic profiling by WGS showed conservation of key driver genes (fig. S20). However, in MB-G3 SJMB03373, a genetic shift occurred in culture at cell passage 3 to 4 that was replicated three times independently, as determined by short tandem repeat (STR) profiling (fig. S22 and table S6) (*34*).

#### Preservation of intratumoral heterogeneity in embryonal PDOXs, TOs, and TOXs

To determine whether the heterogeneity of established PDOX models was maintained in both TO and TOX, we compared transcriptomic profiles using single-cell RNA-seq (scRNA-seq) of PDOX/TO/TOX triad models, including three MB-G3, two MB-SHH, and one ATRT-SHH. Before analysis, we first integrated each quality-filtered triad and subsequently performed unsupervised clustering.

### Group 3 MB

**Fig. 4.**
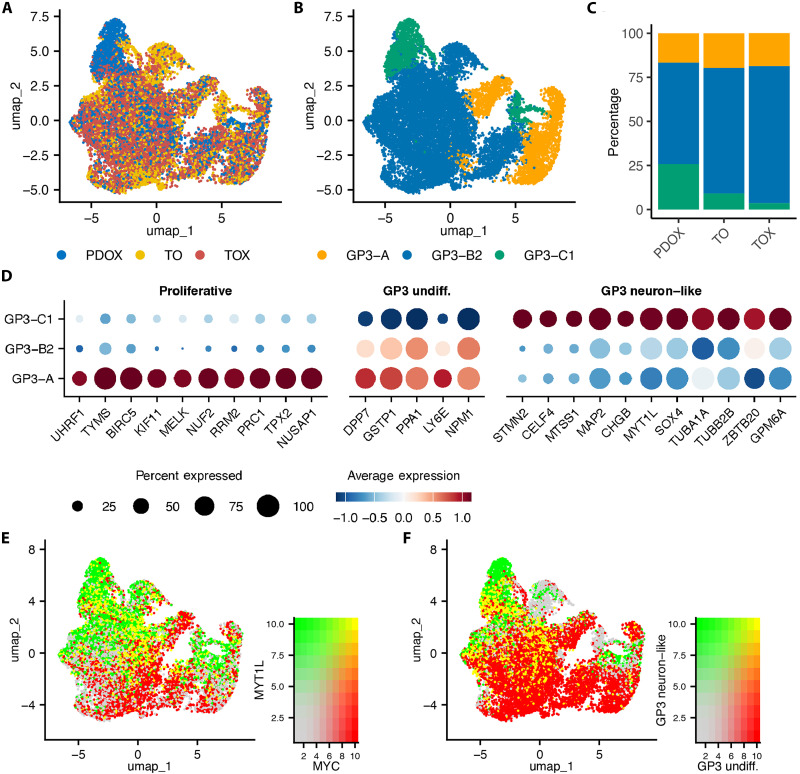
MB-G3 SJMB030315 maintains intratumoral heterogeneity between PDOX, TO, and TOX. (**A**) UMAP plot of the integrated scRNA-seq dataset composed of PDOX (blue), TO (yellow), and TOX (red) cells. (**B**) UMAP plot of the integrated dataset subclusters annotated by cell types previously reported in MB-G3. (**C**) Percentage of annotated cell types in each model from the integrated dataset. (**D**) Dotplot of key proliferative (GP3-A, orange), undifferentiated (GP3-B1, blue), and differentiated (GP3-C, green) cell markers differentially expressed (*P*_adj_ <0.05, percent expressed >50%, logFC >0.25) in annotated subclusters. (**E**) UMAP plot of the integrated dataset with scaled/blended expression of MB-G3 cell markers *MYC* (proliferative/progenitor) and *MYT1L* (neuron-like). (**F**) UMAP plot of the integrated dataset annotated by blended UCell scores using previously reported neuron-like and undifferentiated MB-G3 genesets.

We analyzed three MB-G3 by scRNA-seq (Fig. 4 and figs. S23 to S26). SJMB030315 scRNA-seq dataset showed representation of each annotated cell type in all models (Fig. 4, A to C). Cluster annotation was performed using SingleR (*35*) with previously annotated malignant MB-G3 datasets (*36*) by assigning the mode of cell types classified (fig. S23, A to E). We found clusters with partial differentiated or “neuron-like” signatures (fig. 23, B and C), and the continuum of differentiated/undifferentiated cell types was determined with UCell geneset ranking using the top 30 genes for each cell type/metaprogram previously reported (Fig. 4, B and C, and fig. S23, F to J) (*37*, *38*). Cluster annotation was confirmed and visualized using known cell type markers and UCell metaprogram scores (Fig. 4, D to F, and fig. S23, K to P) (*37*). In SJMB030315, we identified malignant cell types including GP3-A (characterized as proliferating cells), GP3-B2 (characterized as undifferentiated or progenitor cells), and GP3-C (characterized as differentiated or neuron-like cells). The top most differentially expressed genes for each annotated cluster showed a distinct program-specific signature: GP3-A annotated cells show high expression of cell cycle genes (*UHRF5*, *TYMS*, *BIRC5*, *KIF11*, *MELK*, *NUF2*, *RRM2*, *PRC1*, *TPX2*, and *NUSAP1*); GP3-B2 cells show high expression of genes involved in immune cell cytotoxicity suppression, ribosome biogenesis, DNA synthesis, metabolism, undifferentiated cell types, and “stemness” (*DPP7*, *MYC*, *PPA1*, *GSTP1*, *LY6E*, and *NPM1*); and GP3-C annotated cells show high expression of neuron-like or differentiated cell types (*STMN2*, *CELF4*, *MTSS1*, *MAP2*, *CHGB*, *MYT1L*, *SOX4*, *TUBA1A*, *ZBTB20*, and *GPM6A*) (Fig. 4, D and E). Overlap in expression between GP3-A and GP3-B2 was expected as many undifferentiated marker genes are also cell cycle genes, (including *MYC*, *GSPT1*, *PPA1*, and *NPM1*). Consistent with this finding, *MYC* expression levels in MB-G3 SJMB030315 were highest in proliferative (GP3-A) and undifferentiated (GP3-B) cells (Fig. 4E), further supported by the UCell MB-G3 undifferentiated cell type scores (Fig. 4F). Comparison of the TMEs between SJMB030315 PDOX and TOX using mouse cells in both scRNA-seq datasets (fig. S24) identified annotated clusters that show similar representation by model (fig. S24, A to C), as determined by SlimR/CellMarker2.0 annotation (*39*, *40*). Markers of myeloid cells, monocyte-derived macrophages, and Bergmann glial cells were found in their corresponding annotated clusters 0, 1, and 2, respectively (fig. S24D). We observed similar maintenance of intratumoral heterogeneity in each MB-G3 dataset analyzed, SJMB303015 and SJMB032425 (figs. S25 and S26).

### Sonic hedgehog MB

**Fig. 5.**
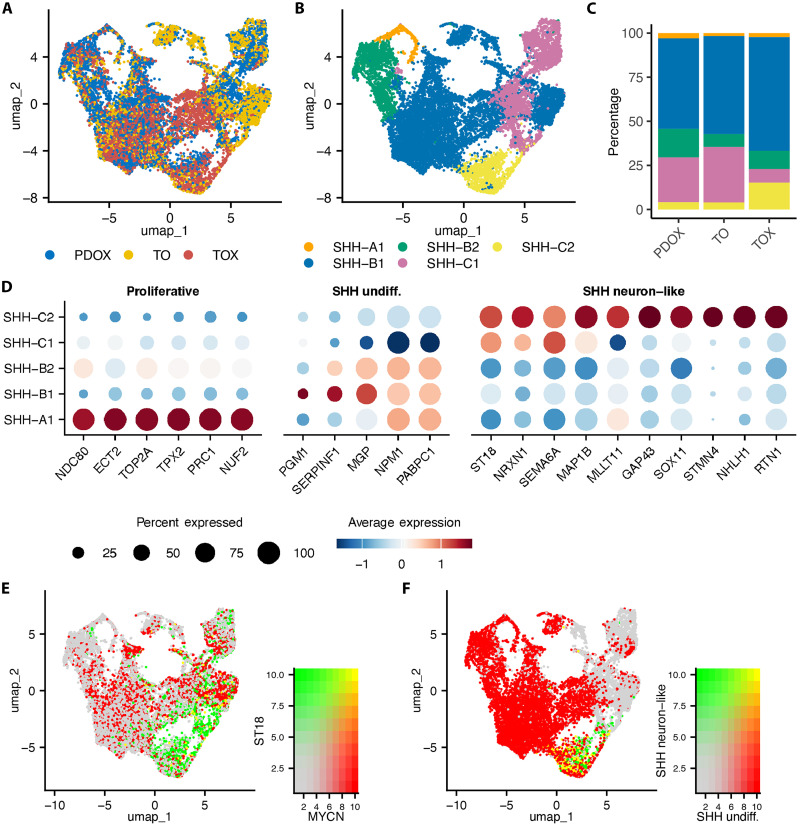
MB-SHH SJMB016874 maintains intratumoral heterogeneity between PDOX, TO, and TOX. (**A**) UMAP plot of the integrated scRNA-seq dataset composed of PDOX (blue), TO (yellow), and TOX (red) cells. (**B**) UMAP plot of the integrated dataset subclusters annotated by cell types previously reported in SHH MB, including proliferative (SHH-A1, orange), undifferentiated (SHH-B1,2, blue and green), and differentiated (GP3-C1,2, pink and yellow). (**C**) Percentage of annotated cell types in each model from the integrated dataset. (**D**) Dotplot of key proliferative, undifferentiated, and differentiated cell markers differentially expressed (*P*_adj_ <0.05, percent expressed >50%, logFC >0.25) in annotated subclusters. (**E**) UMAP plot of the integrated dataset with scaled/blended expression of SHH MB cell markers *MYCN* (proliferative/progenitor) and *ST18* (neuron-like). (**F**) UMAP plot of the integrated dataset annotated by UCell scores using previously reported neuron-like and undifferentiated SHH MB genesets.

We analyzed two MB-SHH models in the same manner as for MB-G3: SJMB016874 (Fig. 5 and fig. S27) and SJMB016876 (figs. S28 and S29). Similar to the MB-G3 PDOX/TO/TOX datasets, maintenance of each annotated cell type was seen in each MB-SHH model set (Fig. 5, A to C, and figs. S27 and S28). We found that SHH-A cells showed a marker expression pattern consistent with proliferating cells (*NDC80*, *ECT2*, *TOP2A*, *TPX2*, *PRC1*, and *NUF2*), and SHH-B cells were consistent with undifferentiated cell types, highly expressing marker genes (*PGM1*, *SERPINF1*, *MGP*, *NPM1*, and *PABPC1*) (Fig. 5D). *MYCN* gene expression was highest in proliferative and undifferentiated cell types (Fig. 5D and fig. S27). In SHH-C–annotated cells, we identified SHH neuron–like markers including *ST18*, *NRXN1*, *SEMA6A*, *MAP1B*, *MLLT11*, *GAP43*, *SOX11*, *STMN4*, *NHLH1*, and *RTN1*.

While intratumoral heterogeneity was maintained in MB-SHH SJMB016876, none of the cells were annotated as neuron-like or differentiated, and we only detected a slight neuron-like/differentiated signature (fig. S28) (*36*). Consistency between MB-SHH SJMB016876 PDOX and TOX TME was observed, with cell type–specific marker expression including macrophages and oligodendrocyte precursor cells corresponding to their respective annotated clusters (fig. S29).

### Atypical teratoid rhabdoid tumors

**Fig. 6.**
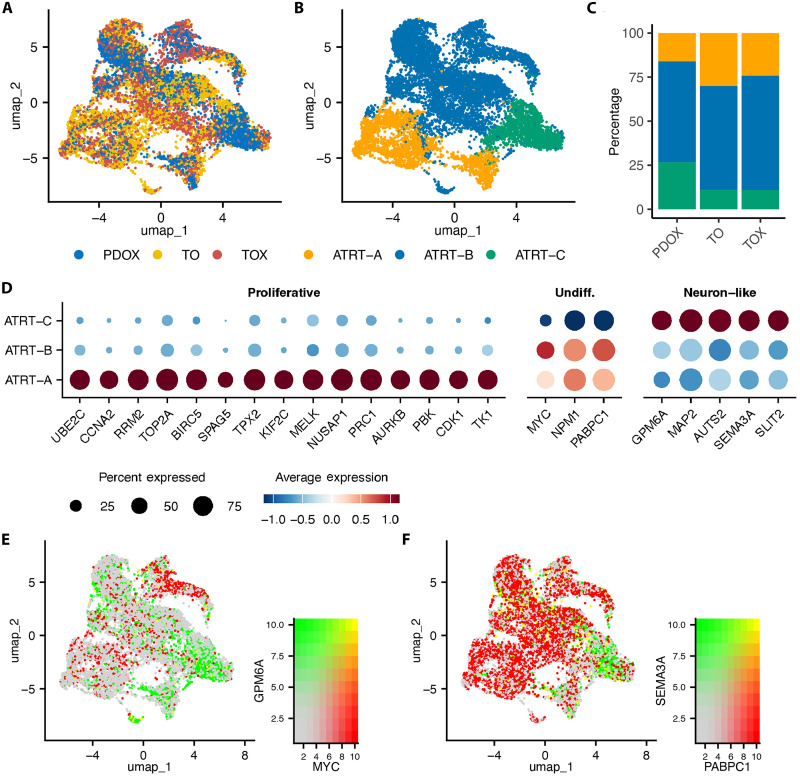
ATRT-SHH SJATRT041800 maintains intratumoral heterogeneity between PDOX, TO, and TOX. (**A**) UMAP plot of the integrated scRNA-seq dataset composed of PDOX (blue), TO (yellow), and TOX (red) cells. (**B**) UMAP plot of the integrated dataset subclusters annotated by UCell analysis and cell marker expression named in a similar fashion for consistency: proliferative (ATRT-A, orange), undifferentiated (ATRT-B, blue), and neuron-like (ATRT-C, green). (**C**) The percentage of annotated cell types in each model from the integrated dataset. (**D**) Dotplot of key proliferative, undifferentiated, and neuron-like/axonogeneis cell markers differentially expressed (*P*_adj_ <0.05, percent expressed >50%, logFC >0.25) in annotated subclusters. (**E**) UMAP plot of the integrated dataset with scaled/blended expression of *MYC* (proliferative/undifferentiated) and *GPM6A* (neuron-like). (**F**) UMAP plot of the integrated dataset with scaled/blended expression of *PABPC1* (undifferentiated) and *SEMA3A* (axonogenesis).

The ATRT-SHH SJATRT041800 dataset showed representation of three distinct cell signatures, maintenance of which was found in each model type (Fig. 6, A to C). Without ATRT-specific gene sets or annotated reference scRNA-seq datasets, the annotation of these cell types relied on differentially expressed cell marker expression and UCell geneset analysis (Fig. 6, D to F, and fig. S30). Three geneset signatures were enriched in a subcluster-specific manner by UCell analysis, which broadly fits as proliferative (using HALLMARK_G2M_CHECKPOINT as a surrogate for cell cycle/proliferating cells), “undifferentiated” (HALLMARK_MYC_TARGETS_V1), and “neuron-like/differentiated” signatures (GOBP_AXONOGENESIS) (fig. S30). Specific gene expression of these signatures included proliferative markers (*UBEC2*, *CCNA2*, *RRM2*, *TOP2A*, *BIRC5*, *SPAG5*, *TPX2*, *KIF2C*, *MELK*, *NUSAP1*, *PRC1*, *AURKB*, *PBK*, *CDK1*, and *TK1*), undifferentiated markers (*MYC*, *NPM1*, and *PABPC1*), and neuron-like genes/axonogenesis (*GPM6A*, *MAP2*, *AUTS2*, *SEMA3A*, and *SLIT2*) (Fig. 6D and fig. S30). Similar to our previous comparative analyses of TME in MB PDOX and TOX models, ATRT-SHH SJATRT041800 showed representation of most annotated cell types, the two largest by cell count including myeloid cells, and macrophages (cluster 0 and 1, respectively) (fig. S31). Both PDOX and TOX cells were represented for each classified cell type, except for D2 medium spiny neurons, for which no PDOX cells were annotated (fig. S31, C and D).

#### IHC and IF analyses of PDOX, TO, and TOX trios

**Fig. 7.**
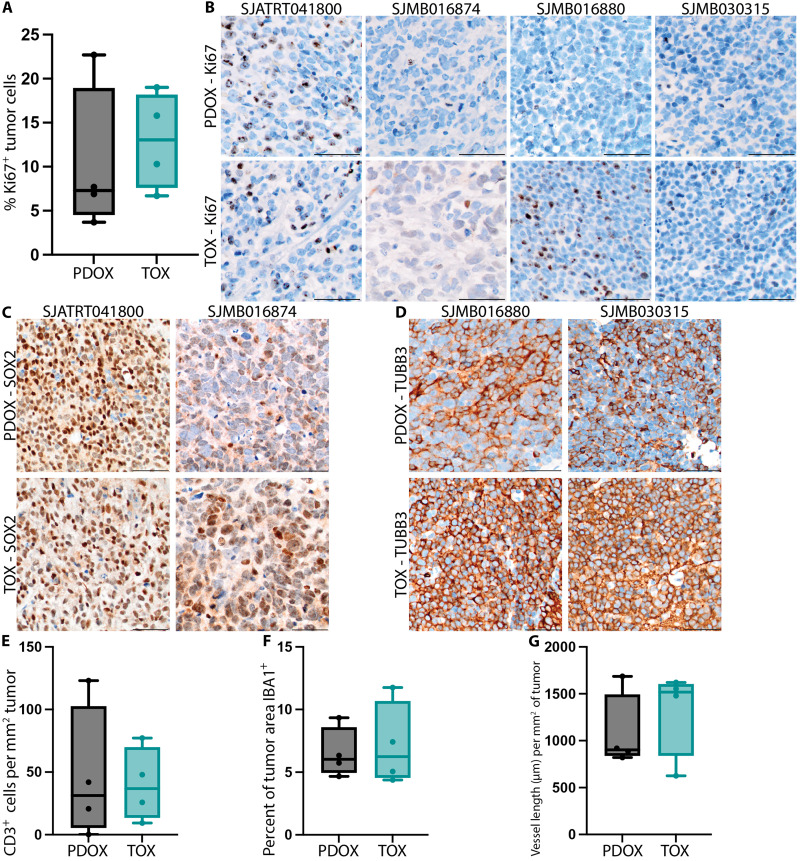
IHC analysis of PDOX and TOX models. (**A**) Quantification of the percent Ki67-positive cells in PDOX or TOX model types. *P* = 0.6857 (**B**) Representative images for Ki67 staining in PDOX and TOX duos: ATRT-SHH SJATRT041800, MB-SHH SJMB016874, MB-G3 SJMB016880, and MB-G3 SJMB030315. Scale bars, 50 μm (**C**) Representative images for SOX2 staining for ATRT-SHH SJATRT041800 and MB-SHH SJMB016874PDOX and TOX duos. Scale bars, 50 μm (**D**) Representative images for TUBB3 for MB-G3 SJMB016880 and MB-G3 SJMB030315 PDOX and TOX duos. Scale bars, 50 μm. (**E** to **G**) Quantifications of TME markers between PDOX and TOX duos (E) CD3 (*P* = 0.8857), (F) IBA1 (*P* ≥ 0.999), and (G) CD34 (*P* = 0.8857). All statistical tests were performed using Mann-Whitney *U* test.

To further characterize the PDOX, TO, and TOX trios, we performed immunohistochemical (IHC) staining on PDOX and TOX pairs and immunofluorescence (IF) staining on TOs. IHC assays showed no significant difference in the proportion of Ki67-positive cells between the PDOX and TOX tumor models (*P* = 0.6857) (Fig. 7, A and B, and table S7). As expected, ATRT-SHH SJATRT041800 and MB-SHH SJMB016874 were positive for SOX2, while the two MB-G3 models, SJMB016880 and SJMB0300315, were positive for B3-tubulin (TUBB3) (Fig. 7, C and D). The number of CD3-positive cells per square millimeter of tumor did not differ significantly between the PDOX or TOX for the four models (*P* = 0.8857) (Fig. 7E and table S7). Similar results were observed for the percent of tumor area positive for microglia (IBA1^+^, *P* => 0.999) (Fig. 7F and table S7) and the length of endothelial vessels per square millimeter of tumor (*P* = 0.8857) (Fig. 7G and table S7). IHC findings for Ki67, SOX2, and TUBB3 were recapitulated by IF assays for TOs of the same models (fig. S32 and table S7).

#### TOs and drug sensitivity

**Fig. 8.**
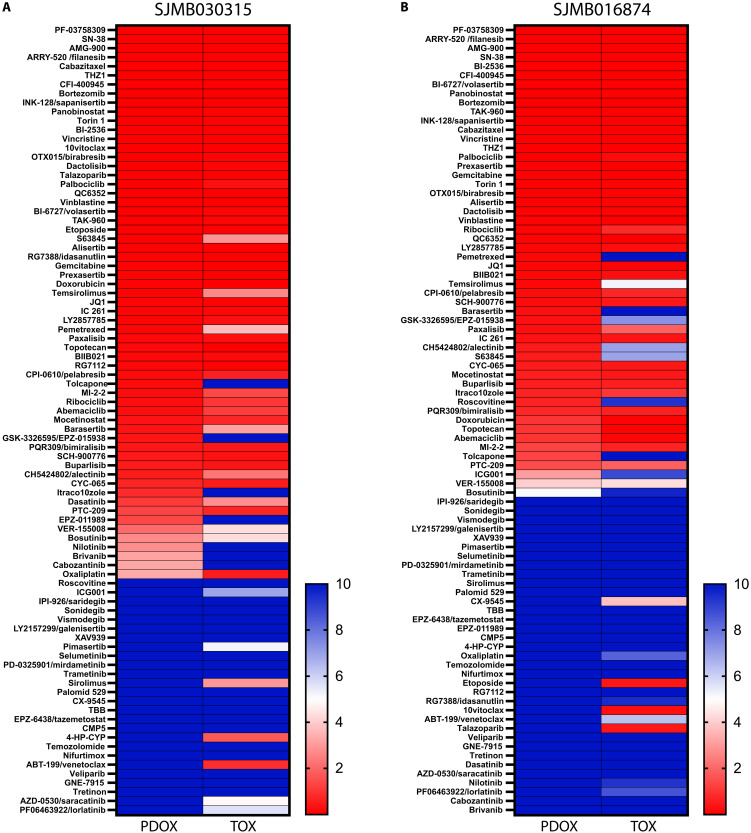
Functional validation of TOs for high-throughput drug screening. EC_50_ (micromolar) heatmaps of 87 single-agent drugs tested in dose response of the PDOX and TO after 7 days of treatment (**A**) MB-G3 SJMB030315, and (**B**) MB-SHH SJMB016874. Raw data are presented in table S7.

We previously reported that MB PDOXs with *MYC* or *MYCN* amplification could be grown transiently in culture from freshly harvested tumors and used for high-throughput drug screening (*20*). We screened two TO models, one MB-G3 and one MB-SHH, with the same 87 compound library, including Food and Drug Administration–approved drugs and those in clinical trials, used for the PDOX they were derived from. Both TOs showed similar sensitivities as their ancestral PDOXs (Fig. 8, A and B, and table S8). MB-G3 TO SJMB030315 showed no statistically different median effective concentration (EC_50_) dose response for 80 of 87 compounds (91.95%, *P* < 0.05, Welch’s *t* test) (Fig. 8A and table S8). This included several CDK inhibitors, including ribociclib (EC_50_ of 0.24 μM in PDOX and 0.08 μM in TO) and other CDK4/6 inhibitors and CHK1/2 inhibitors. MB-SHH TO SJMB016874 also displayed similar therapeutic sensitivity to its original PDOX, with 72 of 87 (82.76%, Welch’s *t* test) of compounds having equivalent EC_50_ values (*P* < 0.05) (Fig. 8B and table S8). MB-SHH TO and PDOXs were also sensitive to CDK4/6 inhibitors in agreement with MB having several genetic alterations of the RB pathway (*41*). MB-SHH were insensitive to MDM2 inhibitors as expected for tumors lacking a functional TP53. Chemotherapeutic drugs currently used in frontline therapy for MB, such as vincristine and etoposide, effectively suppressed the proliferation of MB-G3 and MB-SHH PDOX and TO models. MB-G3 and MB-SHH PDOX and corresponding TOs were insensitive to MEK inhibitors including trametinib, or the alkylating agent temozolomide used to treat gliomas but ineffective in the treatment of MB.

## DISCUSSION

We here present the derivation of multiple PDOXs and a pipeline for the establishment and characterization of PDOXs, TOs, and TOXs trios of embryonal pediatric brain tumors. Since our previous published report (*17*), we have developed an additional 42 PDOXs of 136 primary patient samples from SJCRH and 4 PDOXs from 55 COG samples. In all cases, once primary tumors generated PDOX in NSG mice, they were successfully amplified in CD1-nude mice and propagated orthotopically in the cortices of naïve recipient mice without any culture steps. Comprehensive molecular analysis of PDOXs demonstrated that they recapitulated the primary patient samples. Primary human tumors, including pineoblastoma, pineal parenchymal tumor with intermediate differentiation, pilocytic astrocytoma, choroid plexus carcinoma, and germinoma, did not engraft in NSG mice and did not generate PDOXs (*17*).

MB with *MYC* or *MYCN* amplification, ATRT from both the SHH and MYC subgroups, and ETMR were the only tumors that we could successfully grow as TOs (*23*). Each tumor subgroup necessitated different growth factor requirements for optimal culture conditions. While MB-SHH TOs required PDGF-AA and FGF2, MB-G3 instead benefited from FGF2 and EGF. As previously published, ATRT TOs required EGF, FGF2, PDGF-AA, and PDGF-BB (*23*). This agreed with the transcriptomic expression of their corresponding receptors.

TOs from *MYC* and *MYCN* amplified MB and two ATRT-SHH generated tumors, which aligns with previous reports that some TOs can generate tumors after implantation, a requirement for conducting preclinical studies (*27*). While TOs could be generated from cryopreserved PDOX tumors, the optimal method for TO development began with fresh PDOX resection followed by mouse cell depletion. We found that removal of mouse cells significantly accelerated the development of TOs, likely because mouse cells, such as stromal cells and fibroblasts, compete with the human tumor cells for nutrients and growth factors. We have not attempted to start TO cultures from primary tumors, owing to the small size of the samples received.

Most PDOX-derived tumor cells failed to proliferate after seeding in culture. This failure was probably due to the absence of essential growth factors or a supportive, tumor-promoting microenvironment. Strategies such as coculture with microglia or astrocytes, as well as the identification of necessary growth factors, could help improve in vitro propagation of other tumor types. Ongoing in silico analysis of growth factor receptor gene expression of non-*MYC*– or non-*MYCN*–amplified MB PDOX and other brain tumor models may help identify missing cytokines, chemokines, and growth factors required for TO development.

A key requirement for TO development is maintenance of the molecular characteristics, mutational profile, and intratumoral heterogeneity observed in the primary patient and PDOX tumors. These features are crucial for ensuring that the models faithfully reflect the biology of the disease and allow for accurate preclinical testing of therapeutic responses. We found that when successfully developed, TOs not only preserve the methylation, transcriptomic, and mutational signatures of the original tumors but also retain the cellular heterogeneity observed in PDOXs. Molecular analysis, including methylation signature analysis and both bulk and scRNA-seq for each tumor type: MB-G3, MB-SHH, and ATRT-SHH, including PDOX, TO, and TOX demonstrated that the trios were remarkably similar, indicating that both the TO and TOX models accurately mimicked the PDOX from which they were derived. One exception was the MB-G3 TO, SJMB033373, which exhibited a pronounced difference at the molecular level compared to the PDOX. This event highlights the need to check newly established TOs by STR analysis at each passage during development to ensure that they maintain the PDOX molecular characteristics.

While TOs lacked cells of the TME at the time of analysis, single-cell sequencing of PDOX and TOXs duos showed consistency in the presence of macrophages, myeloid cells, oligodendrocyte precursors, or/and Bergman glia depending on the models studied. These data were confirmed by IHC with markers of microglia and endothelial cells.

To assess whether TOs retain the same drug sensitivity as PDOXs, one *MYC*-amplified MB-G3, SJMB030315, and one *MYCN*-amplified, *TP53* mutant MB-SHH, SJMB016874, were treated with an 87-compound library in high-throughput dose-response assays. There was a remarkably similar response to more than 80% of compounds between the TO and PDOX from both models, highlighting that the developed TOs can act as a surrogate for PDOXs in functional studies. These included CHK, CDK4/6, and BET inhibitors. These data validate recently published findings that the combination of the CHK1/2 inhibitor, prexasertib, with chemotherapeutic agents and the CDK4/6 inhibitor, ribociclib, and gemcitabine was efficient in treating MB-G3 in preclinical trials and is currently evaluated in clinical trials [SJDAWN (NCT03434262), SJELIOT (NCT04023669)] (*20*, *21*, *42*). Similarly, the MB-G3 SJMB030315 TO was susceptible to CHK inhibitors (CHKi), SCH-900776 and S63845, corroborating recent studies showing that ecDNA-containing tumors are susceptible to CHKi (*43*). Not only can TOs be used to identify therapeutic targets but can also be exploited to corroborate findings initially identified in cell lines in a more relevant model before beginning time-consuming and expensive preclinical studies. The ease of manipulation and ability to quickly expand TOs compared to PDOXs make these ideal candidates for large-scale functional studies to accelerate discovery, preclinical, and clinical investigations. Established TOs and TOXs have been provided to collaborators, including from the Pediatric Dependency Accelerator Project (*44*), to study the basic biology of the *MYC* gene family and for preclinical validations of compounds and dependencies.

In summary, we describe a pipeline for the development and validation of TOs of malignant pediatric CNS tumors that recapitulate the primary patient tumor and PDOXs’ molecular identity and intratumoral heterogeneity. We demonstrated that several TOs can be propagated orthotopically in mice, allowing for functional manipulation and subsequent implantation, bypassing many limitations, including the long latency periods encountered when using PDOX models alone. These TOs will facilitate the investigation of basic and translational biology of pediatric brain tumors (*45*) and accelerate the development of preclinical trials before launching clinical trials.

## MATERIALS AND METHODS

### Development of PDOXs

PDOXs were developed as described previously (*17*). Briefly, primary tumors from the operating room or from autopsies were implanted first into NOD.Cg-Prkdc^scid^ Il2rg^tm1Wjl^/SzJ (NSG) (RRID:BCBC_4142) mice to ensure successful immortalization. Tumors developing in NSG mice were amplified in CD1-nude animals (Charles River, #086NU/NUCD1) (RRID:IMSR_CRL:086) for no more than three passages and cryopreserved for future experiments. CD1-nude mice while lacking T cells, maintain all other immune cells and are less prone to infection facilitating preclinical trials (*17*). All procedures performed in studies involving human participants were in accordance with the ethical standards of the Institutional and/or National research committee and with the 1964 Helsinki Declaration and its later amendments or comparable ethical standards. Formal consent to collect patient samples for the development of PDOXs was reviewed by the SJCRH Institutional Review Board and implemented under protocol NBTP01. All animal studies were approved by the Animal Care and Use Committee protocol number 0378-100630 and performed in accordance with best practices outlined by the National Institutes of Health Office of Laboratory Animal Welfare. OLAW:D16-00043; AAALAC:429.

### TO cultures and derivative tumors

Tumor cells were dissociated from cryopreserved vials or fresh tumors as described previously (*17*). If tumor cells came from fresh tumors, they were dissociated and subjected to mouse cell depletion using the Miltenyi Biotec mouse cell depletion kit (Miltenyi Biotec, #130-104-694) according to the manufacturer’s protocol. Tumor cells were seeded into a single well of an ultralow attachment plate based on cell count: 100,000 to 300,000 in a 96-well; 300,000 to 1,000,000 in a 24-well (if >1,000,000 and <2,000,000, the cells were divided between two wells in a 24-well plate) and >2,000,000 in a 6-well plate (Corning, #7007, #3473, #3471, respectively) in TSM full or EFab media supplemented with insulin (2.5 μg/ml) and l-glutamine and cultured at 37°C with 5% CO_2_ (table S9) (*23*).

Cultures were dissociated mechanically up to 20 times using fire-polished pipettes, counted, and replated as needed. We used the following formula to calculate the doubling time of TO cultures at each passage where cell growth was observed

$${\mathrm{DT}}_{\text{days}}=\frac{T\times \text{ln}\left(2\right)}{\text{ln}\left(X_{e}÷X_{b}\right)}$$

where *T* = time (in days); *X_e_* = final cell count (live and dead cells); and *X_b_* = initial cell count or number of cells seeded (live cells only).

To assay whether we could derive tumors from TOs, they were dissociated and implanted into the cortices of naïve CD1-nude mice from 0.5 to 1.0 × 10^6^ cells per mouse, as previously described (*17*). When tumors formed, they were excised, implanted for amplification, flash frozen for future molecular analysis, or cryopreserved for future studies.

### Growth factor requirement assay

To assess whether all four growth factors FGF, EGF, PDGF-AA, and PDGF-BB present in the TSM medium and required for ATRT TOs were necessary for TOs proliferation derived from PDOXs of G3 and SHH MB, 2 × 10^5^ cells were plated per six wells in ultralow attachment plates in TSM medium with or without a combination of each of the four growth factors. The cells were incubated for 30 to 36 days, either with or without passage, and counted.

### Wide-field microscope imaging

Images of intermediate cultures were collected with either differential interference contrast or phase contrast on a Nikon ECLIPSE Ti inverted microscope system (RRID:SCR_021242) through either a Plan Apo 10×/0.45 differential interference contrast N1 or a Plan Fluor 10×/0.3 Ph1 objective, respectively. Using an Andor Zyla 5.5 sCMOS camera, image exposure was set to 30 ms with a 200-MHz readout rate and pixels were binned 2 × 2. The illuminator voltage was set to 3.5 V. Stitched image acquisition was configured using NIS-Elements JOBS to use an 8% overlap with image registration and shading correction. Stage movement was slowed to 3 mm/s to prevent colony disturbance. Images of cells ready for passaging were collected using bright-field settings with a Nikon Eclipse Ts2 inverted microscope (RRID:SCR_025716) through a Nikon Plan Achromat 10×/0.25 PH1 DL objective using a PrimeCam Intervision 12 camera. PrimeCam Pro software was used for measurement analyses.

### FISH of primary patient samples and TOs

Dual-color FISH was performed on 4-μm paraffin-embedded primary patient tissue sections. Probes were derived from bacterial artificial chromosome (BAC) clones (BACPAC Resources, Oakland, CA) and labeled with either Alexa Fluor 488 or Alexa Fluor 555 fluorochromes (table S10). Probes were codenatured with the target cells on a slide moat at 90°C for 12 min. The slides were incubated overnight at 37°C on a slide moat and then washed in 4 M urea/2x SSC at 25°C for 1 min. Nuclei were counterstained with 4,6-diamidino-2-phenylindole (DAPI; 200 ng/ml; Vector Labs) for viewing on an Olympus BX51 fluorescence microscope equipped with a 100-W mercury lamp; fluorescein isothiocyanate, rhodamine, and DAPI filters; 100× PlanApo (1.40) oil objective; and a Jai CV digital camera. Images were captured and processed using the cytovision v7.3 software from Leica Biosystems (Richmond, IL).

Detection of *MYC* amplification by FISH assay used purified *MYC* promoter BAC DNA (RP11-237F24/8q24/128,690,817–128,824,097mb Hg19) labeled with a red deoxyuridine triphosphate (dUTP; SeeBright 580, Enzo) by nick translation. Chromosome 8 control DNA (D8Z2) was labeled with a green dUTP (SeeBright 496, Enzo). FISH assay for *MYCN* amplification using purified *MYCN* BAC DNA (RP11-1183P10/2p24) was labeled with a red dUTP (SeeBright 580, Enzo) by nick translation. Chromosome 2 control DNA (RP11-527J8/2q11.2) was labeled with a green dUTP (SeeBright 496, Enzo). Probes were combined and hybridized as red/green pairs to metaphase and interphase nuclei using a solution containing 50% formamide, 10% dextran sulfate, and 2× SCC. After an overnight hybridization, the slides were washed, stained with DAPI, and analyzed. Two hundred cells per sample were scored for the presence/absence of *MYC* or *MYCN* amplification. One hundred fifty metaphase cells were scored for each type of amplification (double minutes versus homogenously/high staining region).

### DNAm array

All PDOX, TO, and TOX samples were analyzed using the Illumina Infinium Methylation EPIC or Illumina Methylation EPIC v2.0 bead chip arrays according to the manufacturer’s instructions (Illumina, #20087708). Briefly, genomic DNA (300 to 550ng total) was bisulfite treated using the Zymo EZ-96 DNAm Kit (Zymo Research, #D5004) according to the manufacturer’s instructions. The converted DNA was cleaned up with the ZR-96 Genomic DNA Clean & Concentrator-5 (Zymo Research, #D4067) according to the manufacturer’s instructions. Whole-genome amplification (Illumina, #WG-317-1001) was performed on the bisulfite-converted DNA, and the amplified material was endpoint fragmented and precipitated. DNA pellets were resuspended and hybridized to the Methylation EPIC array for approximately 20 hours. The beadchips were processed by iScan and autoloader using the Methylation NXT scan settings file. Samples were classified using the Molecular Neuropathology MNP v12.5-8 pipeline (*29*). All DNAm array data were processed using R package minfi (v1.52) (RRID:SCR_012830), and only autosomal probes shared between 450K, 850K, and 850Kv2 were used for analysis. Probes were normalized using the single-sample Noob method with dye-bias correction and converted to beta values. UMAPs were generated using the top 5000 most variably methylated probes across samples included in each UMAP analysis, including reference samples from the Capper *et al.* (*29*) reference set, as determined by the median absolute deviation (MAD). Hierarchical clustering and plots were produced using ComplexHeatmap (v2.2.0) (RRID:SCR_017270) (*46*). Copy number variation (CNV) analysis was performed using Conumee 2.0 using normal tissue samples from the Capper *et al.* (*29*) reference set as the reference cohort.

### Whole-genome sequencing

Genomic DNA was extracted from samples using phenol chloroform as previously described (*17*). All samples were sequenced with the NovaSeq 6000 (paired end, 150 cycles, targeting >30× base coverage). Mouse reads were cleansed using bbsplit (parameters: maxindel = 20, ambiguous = “best,” ambiguous2 = “toss”) (*47*). The WGS and WES analysis was performed as previously published (*48*); where available, tumor/normal pairs were compared and variants were retained in a consensus approach. All retained variants required support of ≥2 callers, high read-depth (>3 SDs from the mean), and VAF >0.10. All variants were annotated with Annovar (RRID:SCR_012821) (*49*) and Cosmic Cancer Gene/Variant Census v101 (RRID:SCR_002260) (*50*) and retained if previously reported or not annotated as benign or likely-benign. A REVEL score >0.50 was further applied (*51*). CNV and structural variant (SV) analysis of WGS data was performed using CONSERTING (*52*) and Manta v1.5 (RRID:SCR_022997) (*53*), respectively, and annotated using AnnotSV (*54*).

### Bulk RNA extraction, library preparation, sequencing, analysis

Total RNA was extracted from PDOXs and TOs using TRIzol reagent (Invitrogen, #15596026). RNA quantity was measured using Qubit 3.0 fluorometer (Life Technologies, #Q33216) and RNA quantification, broad range reagents (Thermo Fisher Scientific, #Q10211). Total RNA libraries were prepared using the TrueSeq Stranded Total RNA kit (Illumina, #20020597). Each RNA sample was sequenced using a paired-end approach with the Illumina NovaSeq 6000 platform requesting the following parameters per sample: 100 million reads with 100–base pair (bp) paired end reads. Mouse reads were cleansed using bbsplit (parameters: maxindel = 100000, ambiguous = best, ambiguous2 = toss). All reads were mapped to human genome version GENCODE GRCh38v31 with STAR (v2.7.1) (RRID:SCR_004463) (*55*) and processed with RSEM (v1.3.1) (RRID:SCR_000262) (*56*). RNA-seq profiles were normalized using R package DESeq2 (v1.42.0) (RRID:SCR_015687) (*57*), and the variance stabilized gene counts of the 1000 most variably expressed genes (by MAD) were hierarchically clustered and plotted using ComplexHeatmap (v2.2.0) (*46*, *58*). Gene fusions were analyzed using STAR-fusion (RRID:SCR_025853) (*59*), annotated using ChimerDBv4 (*60*), and visualized using ChimeraViz (*61*). Previously reported gene fusions, and in-frame gene fusion events were retained and reported.

### 10X single-cell RNA-seq library preparation and sequencing

PDOX and TOs were dissociated into a single-cell suspension. If tumors were extracted from a mouse tumor, mouse cells were depleted with the mouse cell depletion kit from Miltenyi Biotec (Miltenyi Biotec #130-104-694) according to the manufacturer’s protocol. All samples were subjected to fluorescence-activated cell sorting for live singlets before processing for the 10X Genomics Chromium Next GEMM Single Cell 3′ GEM v3.1 (10X Genomics, #1000121) chemistry according to the manufacturer’s protocol with a targeted recovery of 7000 cells. Microfluidics chips were run on the 10X Genomics Chromium X machine. Final libraries were run on an Illumina NovaSeq 6000 requesting the following parameters per sample: 300 million reads with 100-bp paired end reads.

### 10X single-cell sequencing data analysis

All scRNA-seq datasets were cleansed of contaminating mouse cells using XenoCell (v2.1.0) (*62*) with default values. Host versus graft specific cells were defined as having >90% mapped reads to their respective genomes (human/graft: GRCh38-2024-A, mouse/host: GRCm39-2024-A), and reads were processed with CellRanger (v8.0.1) (RRID:SCR_023221) (*38*). Potential multiplets were removed using DoubletFinder (v2.0.4) (RRID:SCR_018771) (*63*), and a cellular percentage of 20.0% threshold was applied to mitochondrial reads. Additional filters were applied: genes in fewer than five cells; cells with 500 < nGenes < 8000, or nUMI <2000. sctransform (SCT) normalization, variable feature selection, and clustering was performed using R package Seurat (RRID:SCR_007322) (*64*). Dataset integration was also performed using Seurat using “rpca” with 5000 features and 20 anchors. Cell type annotation was aided through use of SingleR (RRID:SCR_023120) (*35*) with previously annotated MB scRNA-seq datasets (*36*), and UCell v2.10.1 scoring (RRID:SCR_027109) (*37*) (maxRank = 500), using previously reported MB-specific and MSigDB genesets (RRID:SCR_016863) (*65*, *66*). Mouse cell type annotation was performed using SlimR (*39*) with CellMarker2.0 (RRID:SCR_018503) (*40*) (species: “Mouse,” tissue_class: “Brain”) using default values. Adaptive thresholds used to determine clusters with a differentiated MB cell signature was determined as $\text{quantile}\;(\text{cellscor}e_{i},\text{probs}=\text{diffcell}.\text{threshold})>0_{i=1}^{\text{nCluster}}$, where diffcell.threshold= $2\left(\frac{\text{nDiffCells}}{\text{nCells}}\right)$, $\text{nDiffCells}=\sum_{i=1}^{\text{nCells}}1_{\left\{\text{cellscor}e_{i}>0\right\}}$, and $\text{cellscore}={\left\{\text{log}2(\frac{\text{UCell Differentiate}d_{i}+0.01}{\text{UCell Undifferentiate}d_{i}+0.01})\right\}}_{i=1}^{\text{nCells}}$.

### IHC staining and analysis

IHC was performed on formalin-fixed paraffin-embedded tissues sectioned at 4 μm. All assay steps for SOX2 (Abcam, ab97959, 1:250), TUBB3 (BioLegend, 801202, 1:9000), Ki67 (R&D, MAP7617, 1:500), CD3 (Santa Cruz Biotechnology, sc-1127, 1:500), and IBA1 (BioCare Medical, CP290A, 1:300), including deparaffinization, rehydration, and epitope retrieval, were performed on the Ventana DISCOVERY Ultra (RRID:SCR_021254) with Ventana Reaction Buffer (Ventana Medical Systems, 950-300) rinses between steps. Antibody binding was detected using the OmniMap Rabbit Detection kit (Roche, 760-4311) for 16 min (TUBB3 first labeled with rabbit anti-mouse secondary antibody and CD3 with rabbit anti-goat antibody), followed by ChromoMap DAB (Roche, 760-159) for 10 min. All assay steps for CD34 (PharMingen, 553731, 1:100) were performed on the Bond Max with Bond wash buffer (Leica, AR9590) rinses between steps. Slides were incubated with the primary antibody; antibody binding was detected using the anti-rabbit Bond Polymer Refine Detection kit (Leica, DS9800), with CD34 first labeled with a rabbit anti-rat secondary antibody.

Tumors were determined whether to be positive or negative for SOX2 and TUBB3 by visual evaluation. Tumor slides labeled with Ki67, IBA1, CD3, and CD34 were scanned to a 20× scalable whole-slide image using a PANNORAMIC 250 Flash III slide scanner (3DHISTECH Ltd.) (RRID:SCR_022184). The HALO image analysis program v3.6.4134.137 with the following algorithms (Indica Labs) were used to quantify the IHC after manually annotating the tumor tissue: for Ki67, CytoNuclear v2.0.9; for IBA1, Area Quantification v2.4.3. For CD3, the Area Quantification algorithm was used to measure the total tumor area and CD3-positive cells were manually counted in that area, for a readout of cells per square millimeter of tumor. For CD34, the Area Quantification algorithm was used to measure the tumor area and set a threshold for labeling; then, the length of all intratumoral vessels was measured and combined for a readout of total microns of vessel length per square mm of tumor.

### Statistical analysis of IHC staining

Mann-Whitney *U* test was used to determine dissimilarity between PDOX and TOX cell counts and tumor area for Ki67, CD3, IBA1, and CD34 staining. All relevant statistical values are listed in table S7.

### IF staining and image analysis

Eight-well chamber microscope slides (ibidi, #80841) were coated with 300 μl of 1% growth factor–reduced Matrigel (Corning, #354230) in phosphate-buffered saline (Gibco, #10010023) per chamber and incubated at 37°C for 30 min. Matrigel solution was aspirated, and TOs were seeded in each well at a density of 75,000 cells per well. The cells were simultaneously fixed and permeabilized with 4% paraformaldehyde and 0.5% Triton X-100 for 30 min. The cells were stained with antibodies targeting either Ki67 (abcam, #ab15580), SOX2 (abcam, #ab171380), or TUBB3 (abcam, #ab18207) overnight at 4°C with gentle rocking. Secondary antibodies Alexa Fluor 546 goat anti-mouse immunoglobulin G (IgG, Thermo Fisher Scientific, #A11030) or Alexa Fluor 647 goat anti-rabbit IgG (Thermo Fisher Scientific, #A21245) were incubated for 1 hour at room temperature with gentle rocking. ProLong Diamond with DAPI (Thermo Fisher Scientific, #P36966) was used for mounting media.

Fluorescence imaging was performed using a Nikon Eclipse Ti epifluorescence microscope equipped with an Andor Zyla camera at 20× magnification. Multiple fields of view were acquired per well. Quantification of positive cells was performed using the Cell Detection Analysis module in QuPath (v0.6.0) (RRID:SCR_018257) (*67*) with the following parameters:

DAPI: Requested pixel size = 0.5; background radius = 500; median filter radius = 0; sigma = 1; minimum area = 12; maximum area = 750; threshold = 750; split by shape = off; cell expansion = 0; include cell nucleus = off.

Ki67: Requested pixel size = 0.5; background radius = 500; median filter radius = 0; sigma = 1; minimum area = 12; maximum area = 750; threshold = 2750; split by shape = off; cell expansion = 0; include cell nucleus = off.

SOX2: Requested pixel size = 0.5; background radius = 500; median filter radius = 0; sigma = 1; minimum area = 12; maximum area = 750; threshold = 1000; split by shape = off; cell expansion = 8; include cell nucleus = off.

TUBB3: Requested pixel size = 0.5; background radius = 500; median filter radius = 0; sigma = 1; minimum area = 12; maximum area = 750; threshold = 2750; split by shape = off; cell expansion = 0; include cell nucleus = off.

### High-throughput drug screens

Cell density optimizations for one MB-G3 TO (SJMB030315) and one MB-SHH TO (SJMB016874) were performed as previously published (*20*). Dimethyl sulfoxide (0.08%) and 1.6 μM staurosporine were used as negative and positive controls, respectively. Twenty-four hours before drugging, dissociated cells were filtered through a 40-μm strainer to remove any remaining clumps, counted, resuspended in fresh TSM media containing the appropriate amounts of growth factors and then distributed to 384-well plates using an Integra VIAFILL automated dispenser, at concentration of 4000 cells per well for SJMB030315 and 2000 cells per well for SJMB016874. Compounds were added to the cell plates via an ECHO 655 automated acoustic liquid handler (Beckman Coulter) and ECHO server software. Thirty nanoliters of compounds were transferred to the cell plates in 30 μl of media per well 24 hours after cell plating. Post drugging, the plates were incubated at 37°C with 5% CO_2_. On day 7, replicate plates were subjected to Cell Titer-Glo assay (Promega, #G7375) and luminescence signals collected via an EnVision 2105 Multimode Plate Reader (RRID:SCR_018038) with EnVision Manager (RRID:SCR_016681) software. EC_50_ values were calculated as previously reported (*20*). Data for replicate assays were averaged to determine the final EC_50_ value for each compound. Visualizations were produced using GraphPad Prism 10.4.1 (RRID:SCR_002798).

### Statistical analysis of high-throughput drug screen

Two-tailed Welch’s *t* test was used to determine EC_50_ dissimilarity between PDOX and TO responses. Three replicates for each model type were used for statistical analysis. All relevant statistical values can be found in table S8.
