# Supplementary material for: Patient-derived pediatric brain tumor orthotopic xenografts and tumor organoids faithfully recapitulate primary tumors
Source: Sci Adv. 2026 Apr 15;12(16):eaea4966. doi: 10.1126/sciadv.aea4966 (PMC13235006; doi:10.1126/sciadv.aea4966)
Supplement: Supplementary file 1 — Figs. S1 to S32 Tables S5, S9, and S10 Legends for tables S1 to S4, S6 to S8 [file sciadv.aea4966_sm.pdf]

Science Advances
AAAS

# Supplementary Materials for

## Patient-derived pediatric brain tumor orthotopic xenografts and tumor organoids faithfully recapitulate primary tumors

Justin S. Williams *et al.*

Corresponding author: Martine F. Roussel, martine.roussel@stjude.org

*Sci. Adv.* **12**, eaea4966 (2026)
DOI: 10.1126/sciadv.aea4966

**The PDF file includes:**

Figs. S1 to S32
Tables S5, S9, and S10
Legends for tables S1 to S4, S6 to S8

**Other Supplementary Material for this manuscript includes the following:**

Tables S1 to S4, S6 to S8

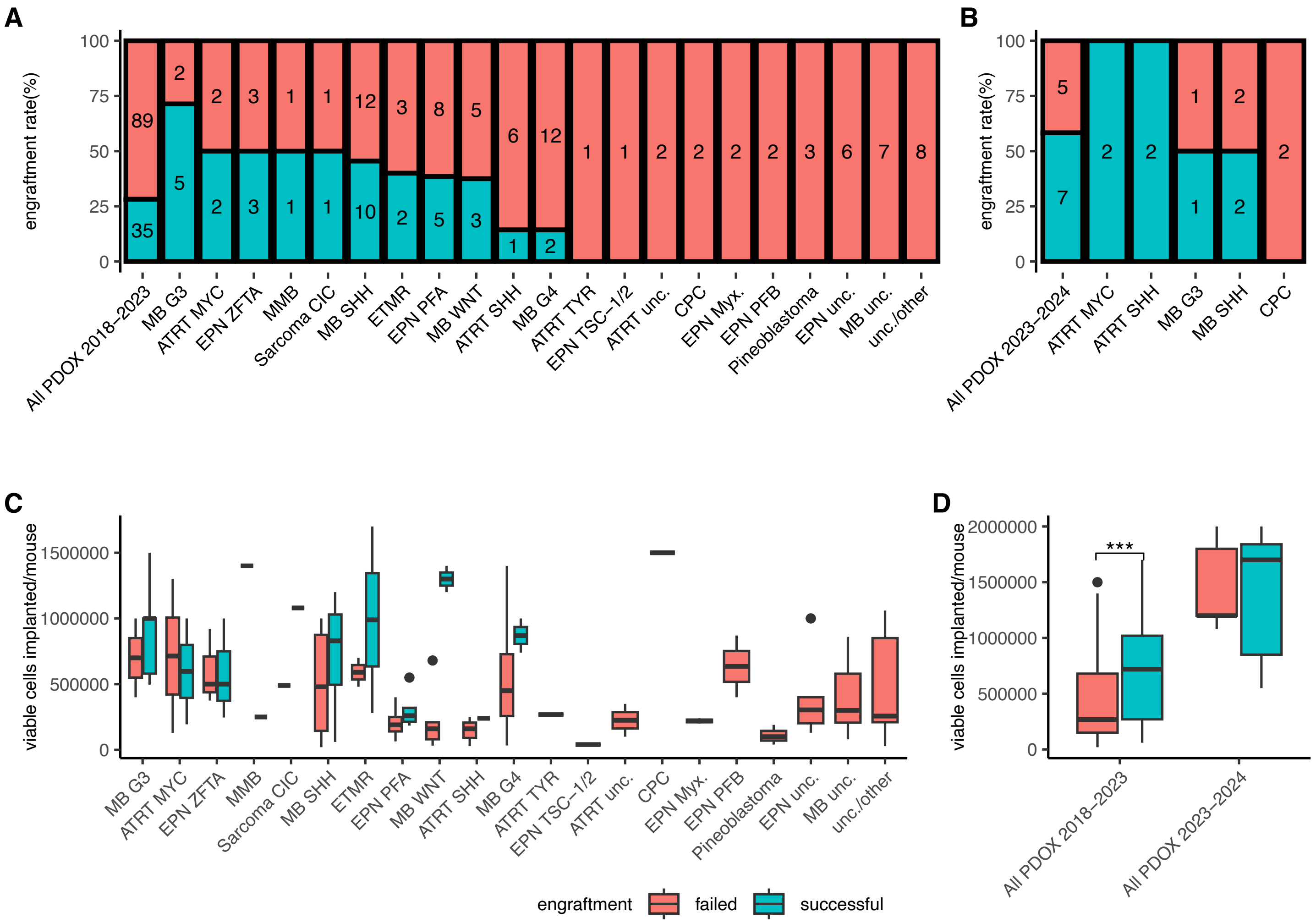


**Fig. S1. Engraftment rate and implanted cell statistics of PDOX models by subgroup.**
(**A-B**) PDOX engraftment rate by cancer subgroup for years 2018-2023 (**A**) and 2023-2024 (**B**). Numbers overlaying bars represent the successful (blue) and failed (red) engraftments. An overall engraftment rate of 28% (35/124) in the years 2018-2023 increased to a success rate of 58% (7/12) for the years 2023-2024 following a median increase of $1{\times}10^6$ viable cells injected per mouse. Unc.: unclassified tumor or subgroup. (**C**) Distribution of viable tumor cells implanted per mouse for the years 2018-2023 by cancer subgroup. (**D**) Distribution of viable tumor cells implanted per mouse for successful and failed engraftments comparing the years 2018-2023 to 2023-2024. Unpaired wilcox test p-value *** < 0.001.

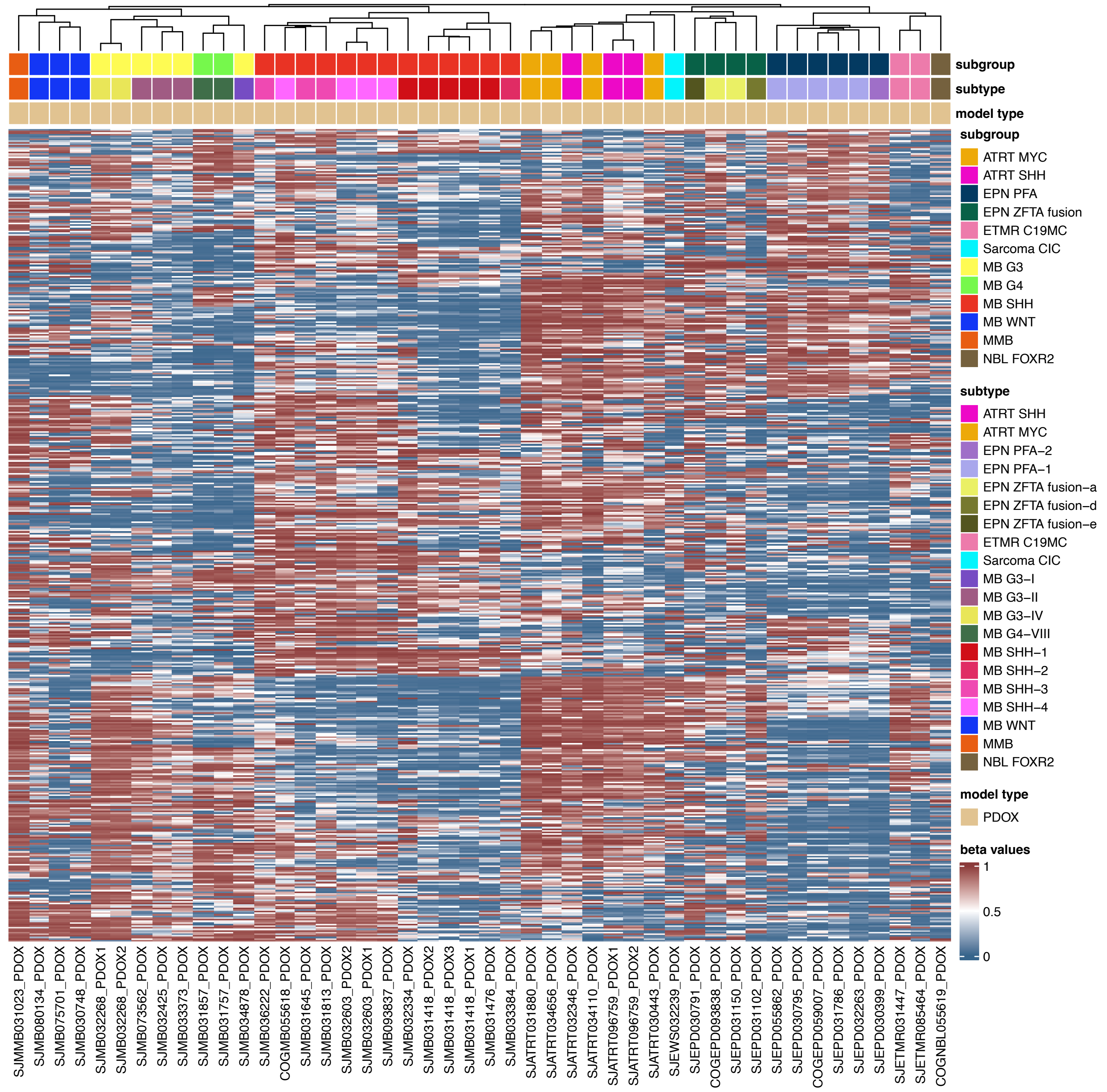


**Fig. S2. Methylation-based clustering of PDOX models.**

Unsupervised hierarchical clustering of the 1000 topmost differentially methylated probes by median absolute deviation.

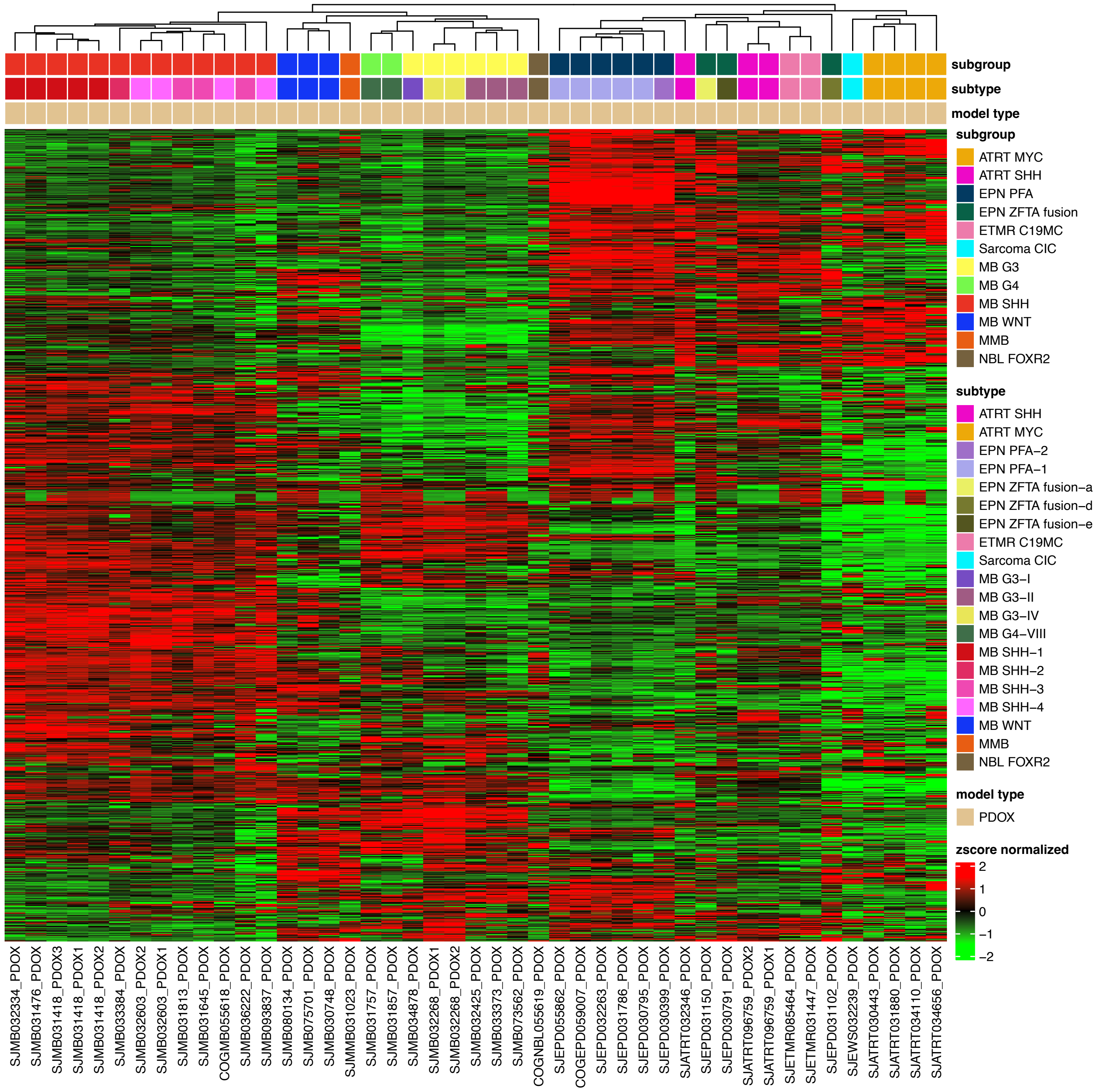


**Fig. S3. Transcriptomic clustering of PDOX models.**
Unsupervised hierarchical clustering of RNA-seq profiles using the 1000 topmost variably expressed genes by median absolute deviation.

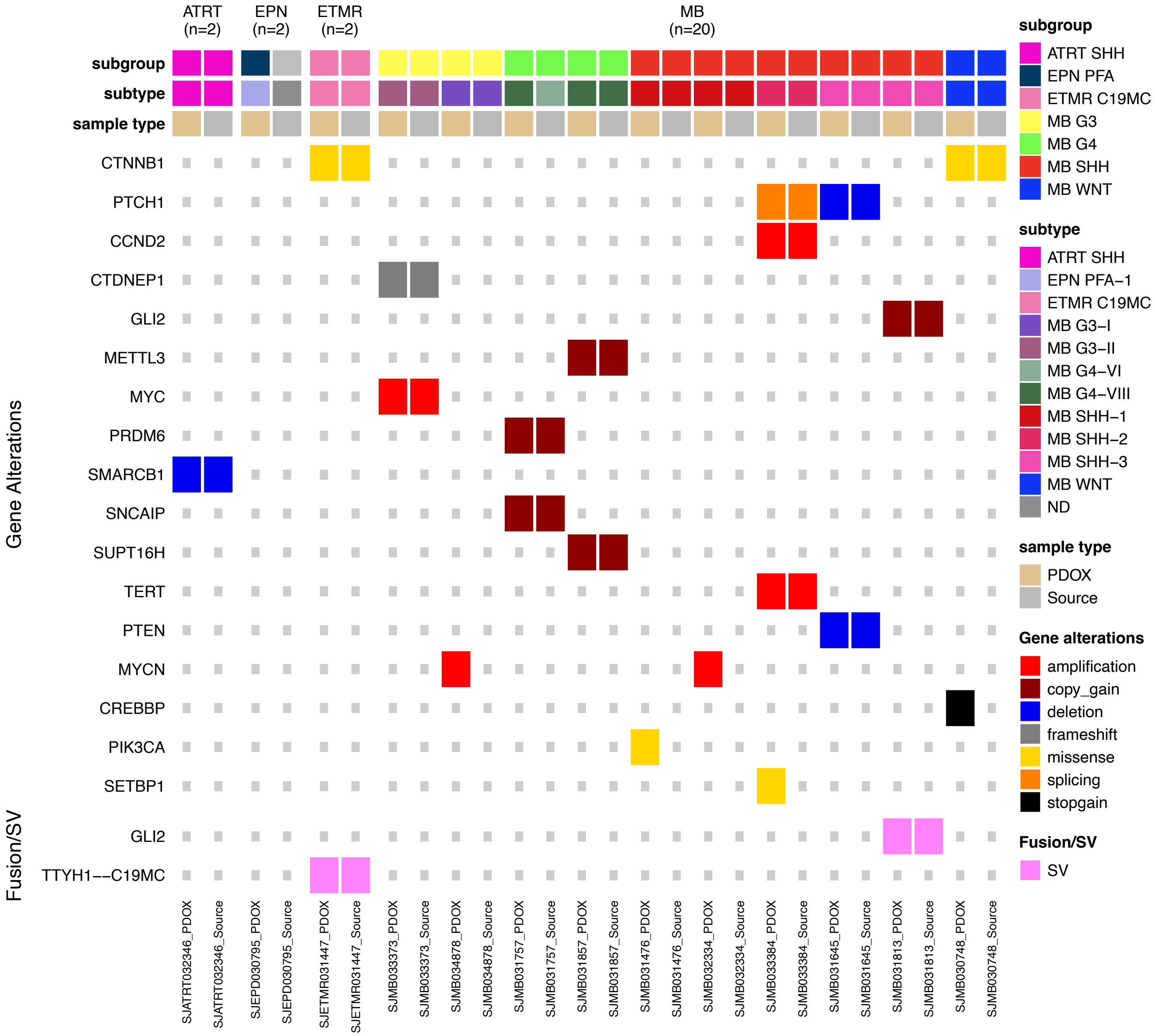


**Fig. S4. Molecular characterization of PDOX primary tumors.**
A comparison of hallmark driver alterations identified in source tumors (where sequencing data were available) and the corresponding PDOX model. Only unpaired WES was available for source tumors SJMB034878 and SJMB032334. A known driver alteration was not determined for source tumor SJMB031476.

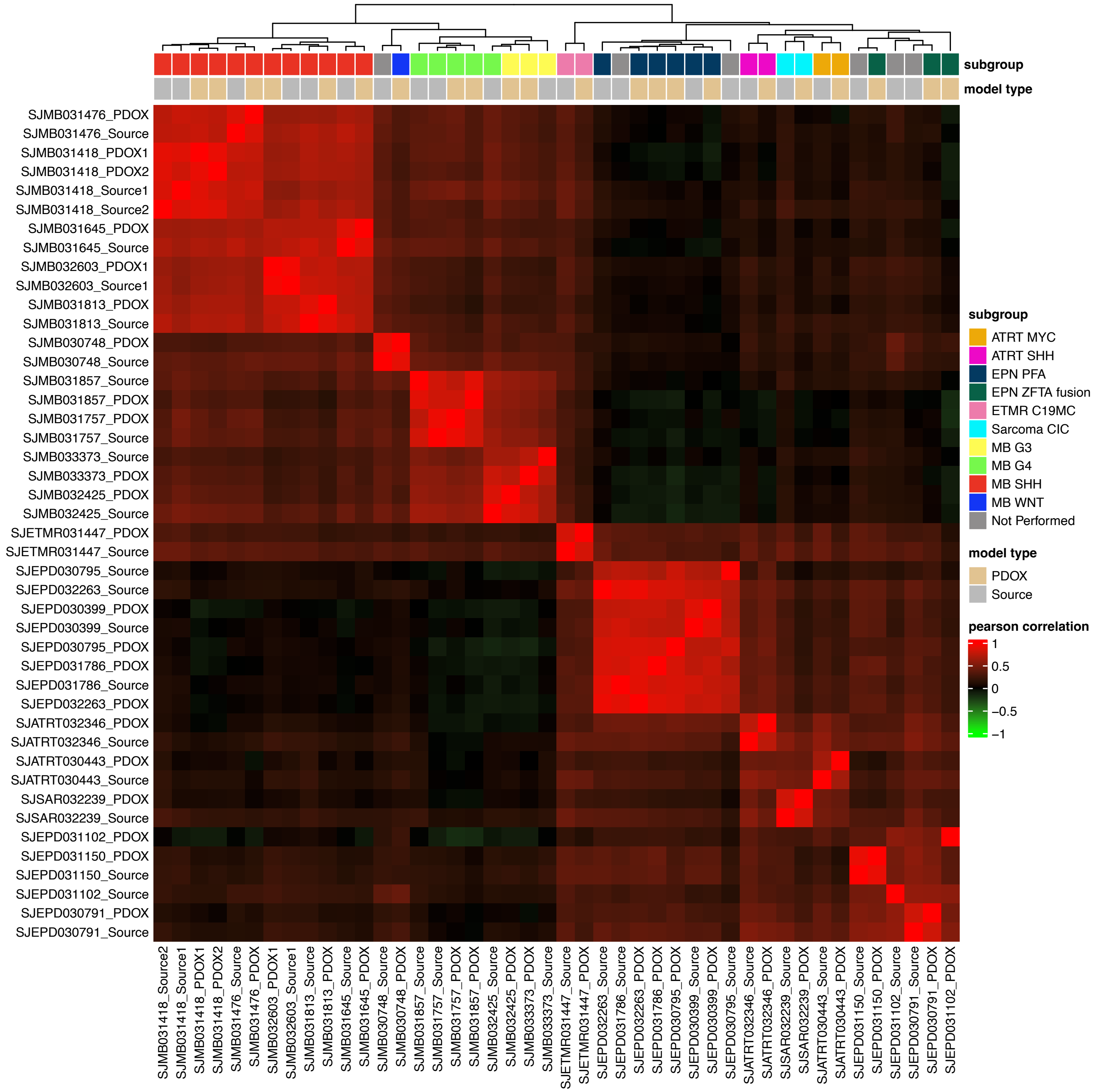


**Fig. S5. Comparison of transcriptomic profiles between PDOX and the matching source tumor.** Hierarchical clustering of transcriptomic profile consistency as determined by pairwise sample Pearson correlation. Pearson correlation was calculated using the top 1000 most variably expressed genes by median absolute deviation.

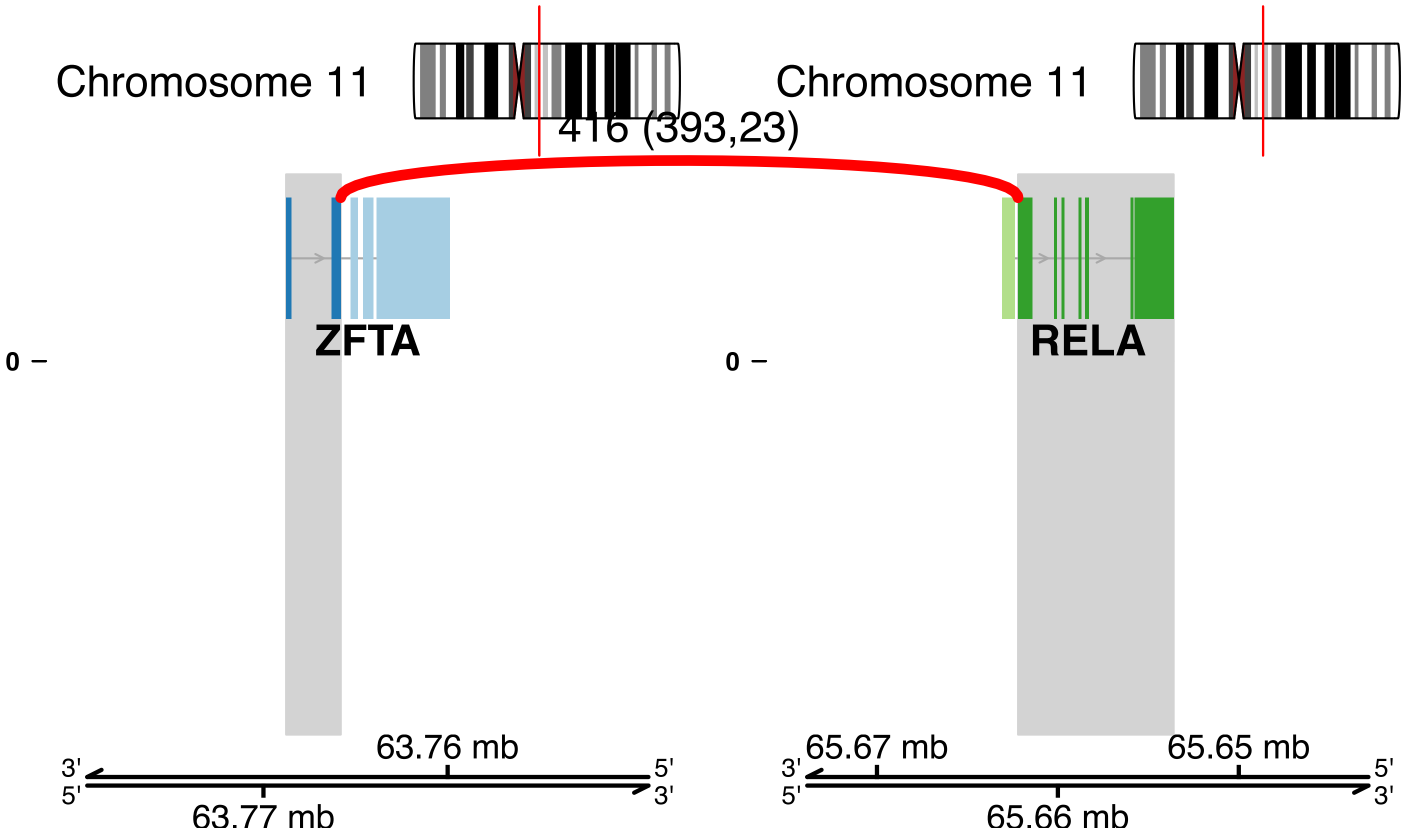


**Fig. S6. Representative EPN ZFTA-RELA fusion.**
Diagram of hallmark gene fusion characterized in ZFTA-RELA fusion ependymomas, including SJEPD031150, SJEPD031102, and SJEPD030791 (both PDOX and source tumor), as detected by STAR-fusion and visualized by ChimeraViz.

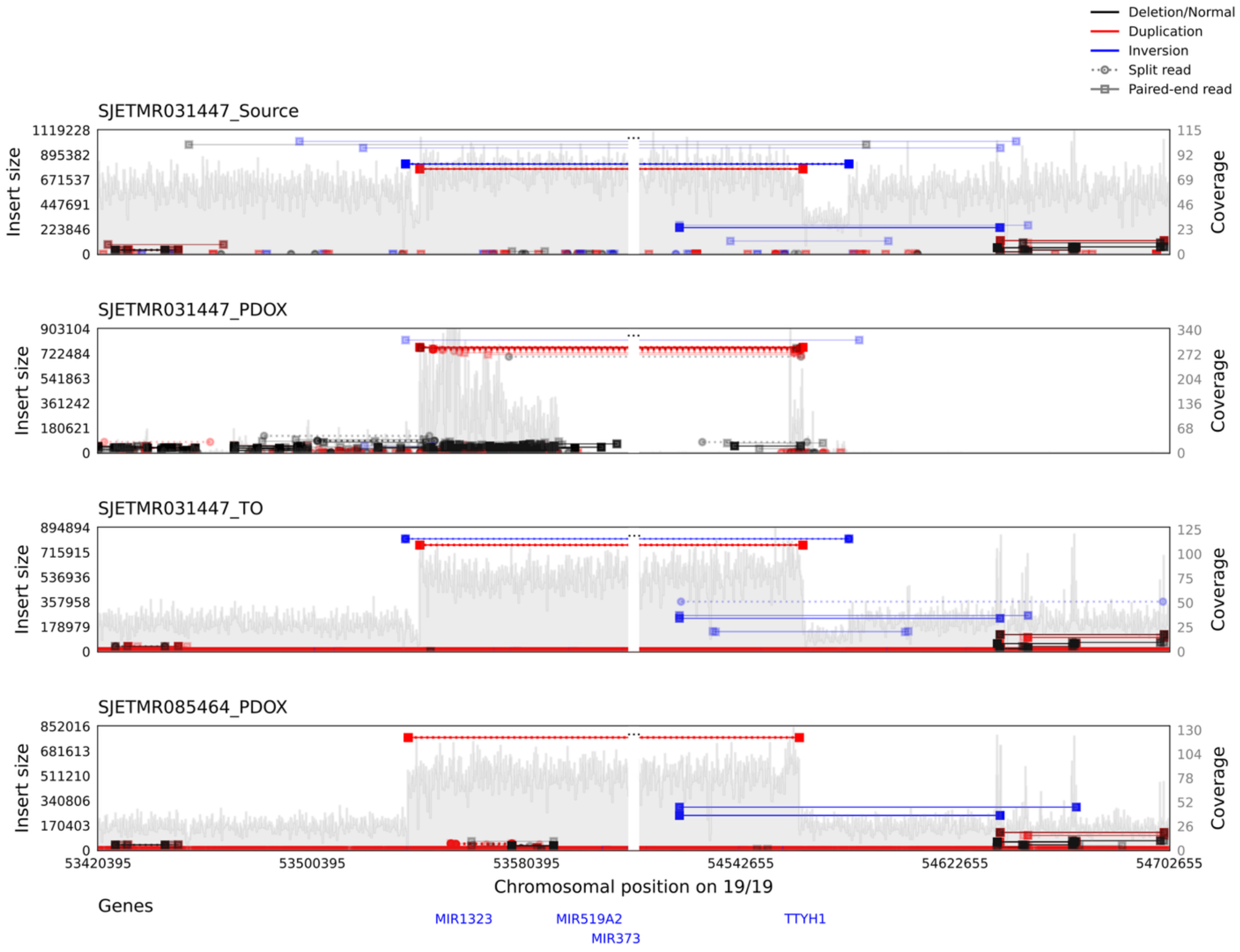


**Fig. S7. ETMR PDOX TTYH1-C19MC rearrangement.**
ETMR C19MC amplified Source, PDOX, and TO model structural variant read support as visualized by samplot. The C19MC locus is demarcated by representative MIR genes. Plots are zoomed in to visualize 500 Kbp (+/-) surrounding breakpoints. Red lines indicate duplications; blue lines indicate inversions. Dotted lines and solid lines indicate split read support and paired read support, respectively. Coverage tracks highlighting copy gain/loss are plotted in gray.

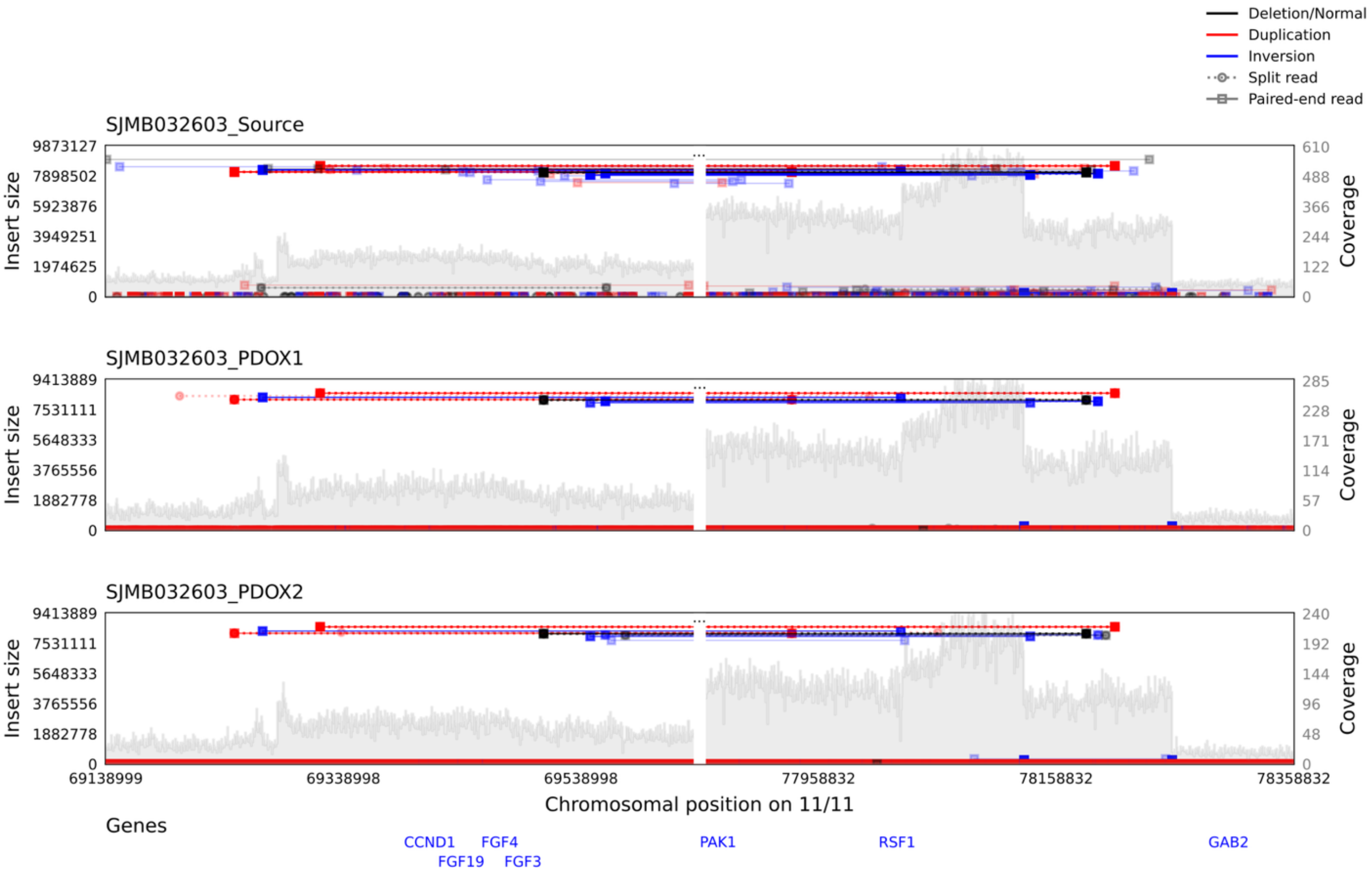


**Fig. S8. SJMB032603 *CCND1*/Chr11 rearrangement.**
SJMB02603 (MB-SHH) Source and PDOX model structural variant read support indicating both duplication and rearrangement of chromosome 11q, harboring *CCND1*. Plots are zoomed in to visualize 500 Kbp (+/-) surrounding breakpoints. Red lines indicate duplications; blue lines indicate inversions. Dotted lines and solid lines indicate split read support and paired read support, respectively.  Coverage tracks highlighting copy gain/loss are plotted in gray.

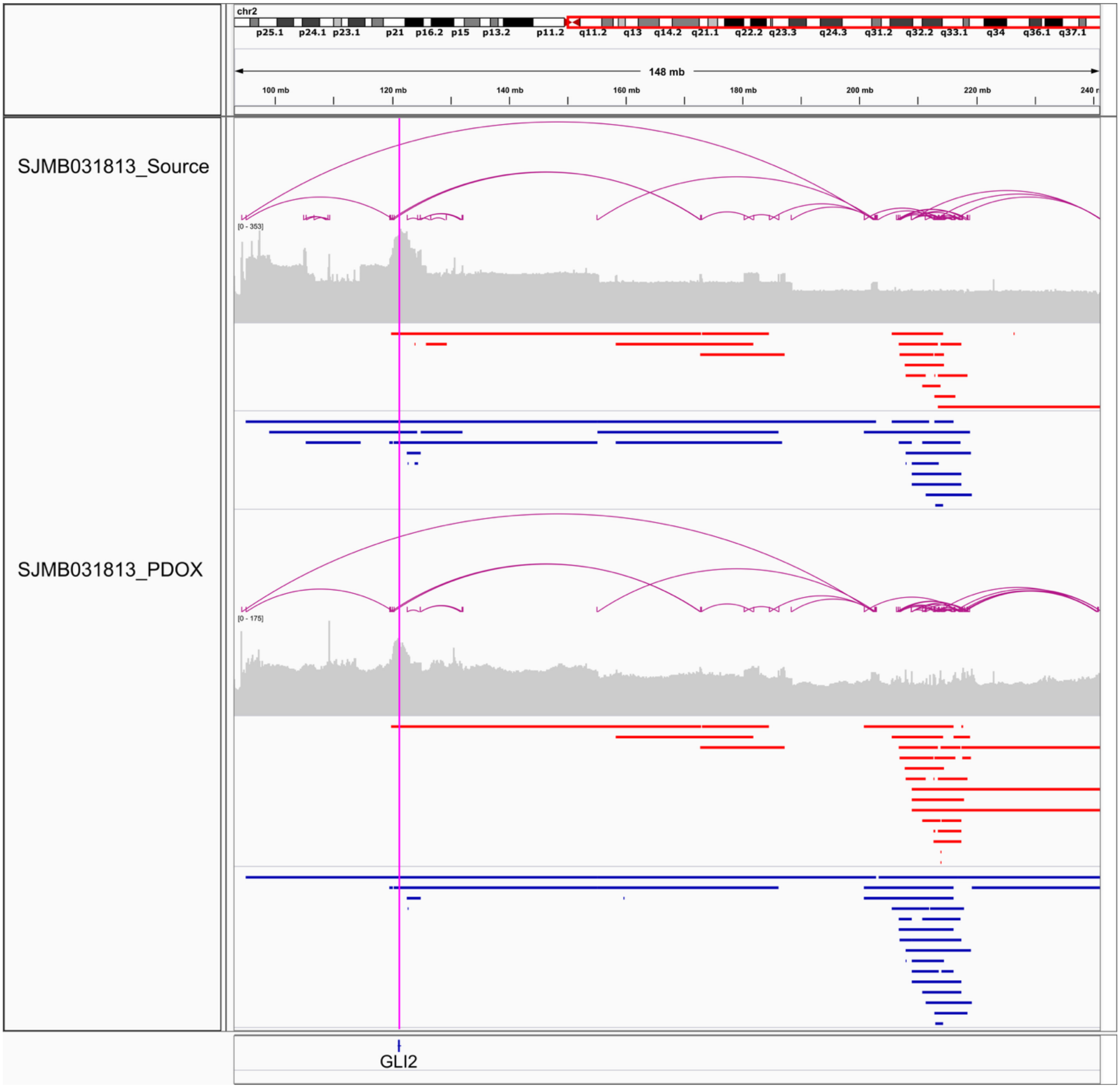


**Fig. S9. SJMB031813 *GLI2* harboring a structural variant.**
SJMB031813 (MB-SHH) Source and PDOX model structural variant call visualization, indicating duplication and rearrangement of chromosome 2q, harboring *GLI2*. Manta structural variant calls, which passed default filters, are presented, including inversions (purple arcs), tandem duplications (red bars), and deletions (blue bars). WGS coverage highlighting copy gain/loss is plotted in gray; *GLI2* is annotated across samples by a magenta bar.

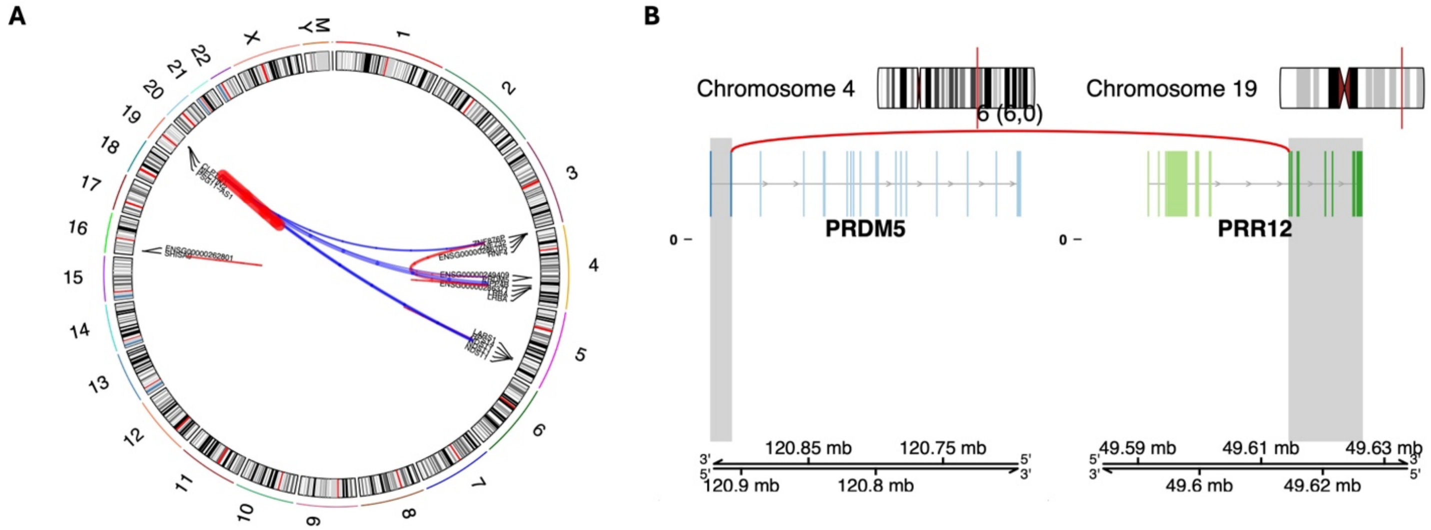


**Fig. S10. SJSAR032239 (Sarcoma CIC-rearranged) PDOX model fusion event visualization.** (**A**) CIRCOS plot summarizing all detected RNA fusions in PDOX SJSAR032239. Intra- and inter-chromosomal fusion events are represented by red and blue lines, respectively. (**B**) An in-frame RNA fusion detected by STAR-fusion and visualized by ChimeraViz, between transcription factor *PRDM5* and DNA binding protein-encoding gene of unknown function, *PRR12*.

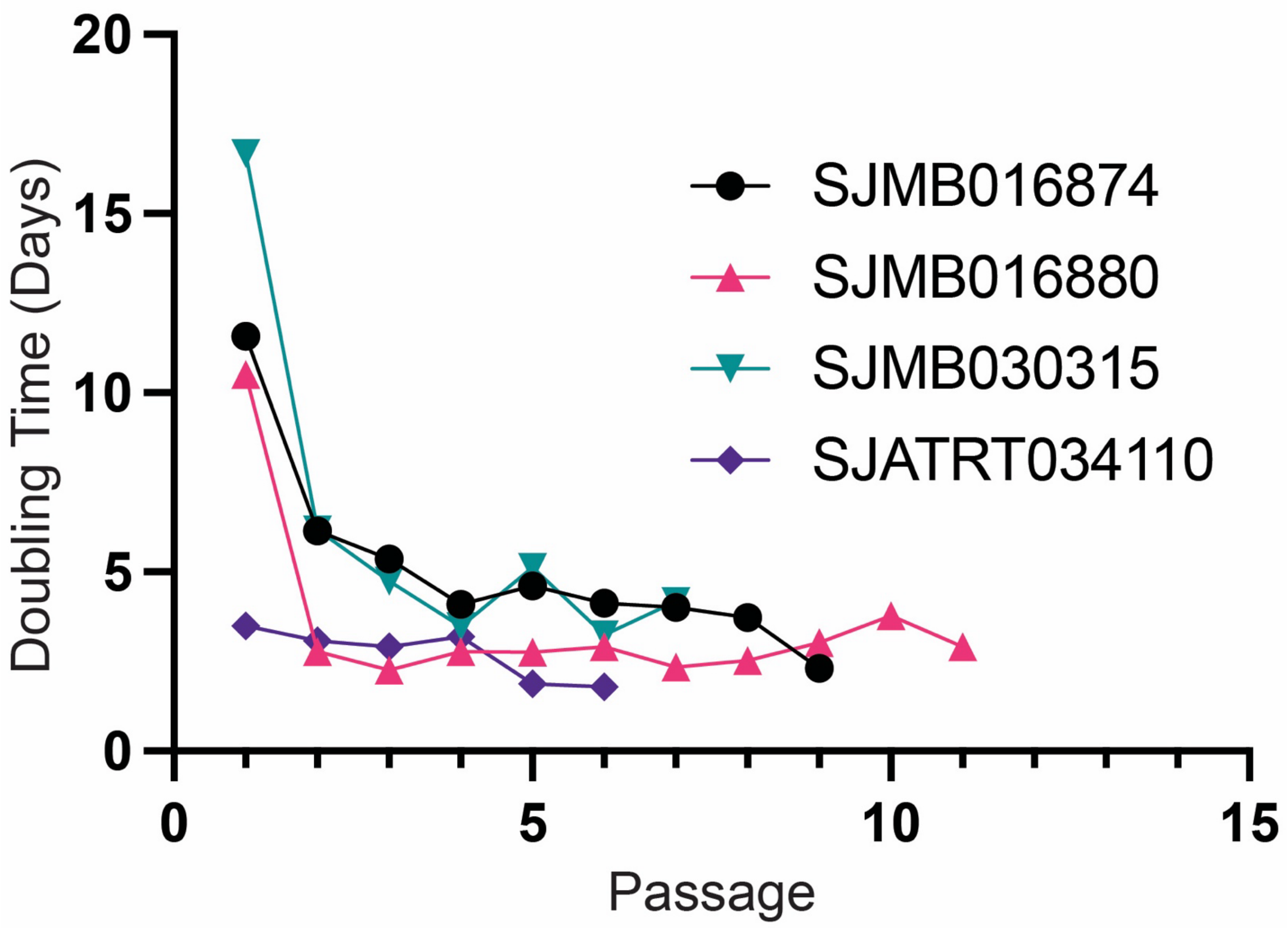


**Fig. S11. Tumor organoid establishment growth curve.**
Growth curves demonstrating the variation in doubling time (days) over multiple non-consecutive passages when tumor organoid growth was observed in cultures.

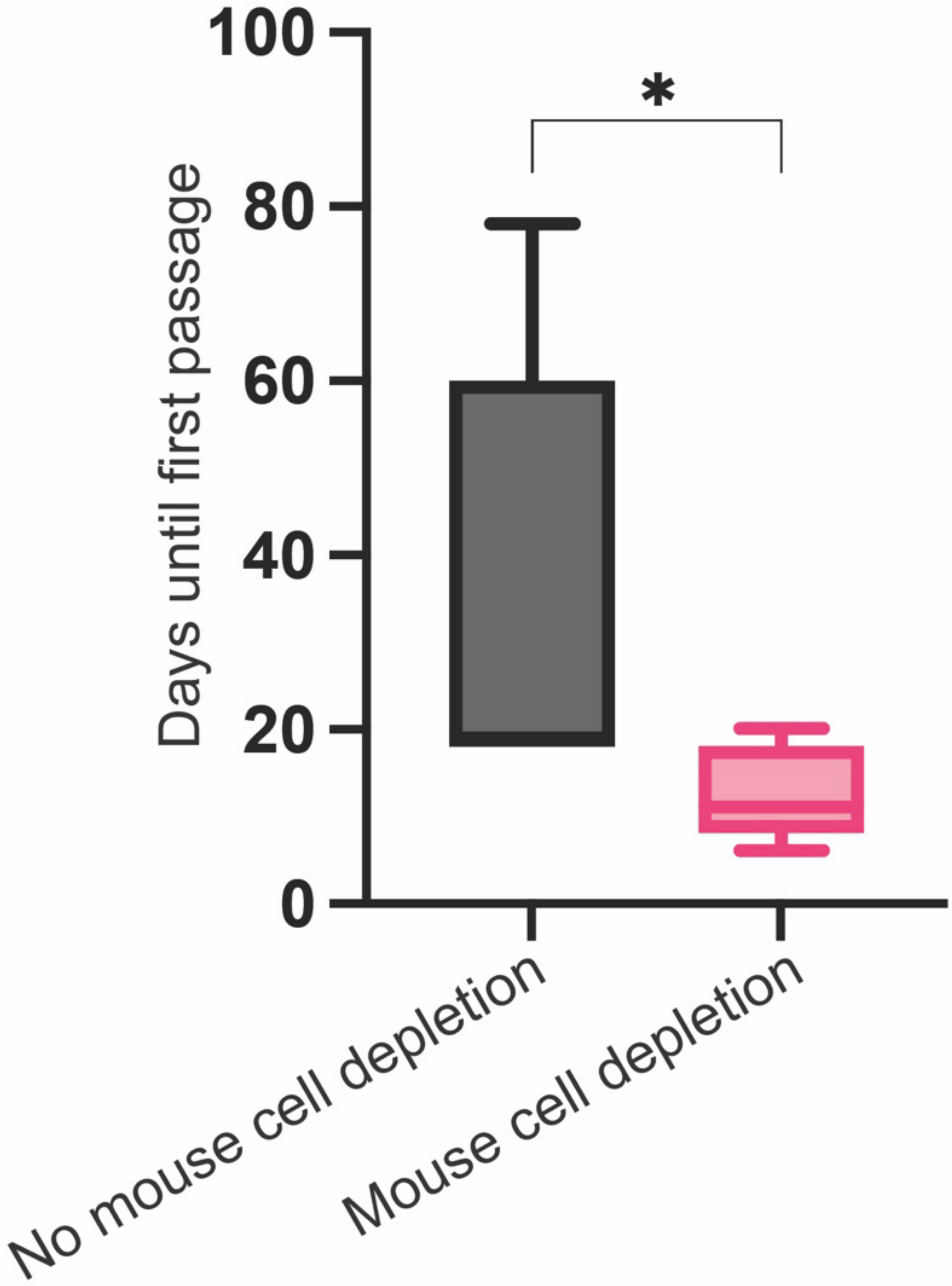


**Fig. S12. Mouse cell depletion decreases time to first passage for tumor organoid establishment.** Number of days from initial seeding until tumor organoid cultures were ready for first passaging. Non-parametric t-test, p = 0.0476, n = 5 per group.

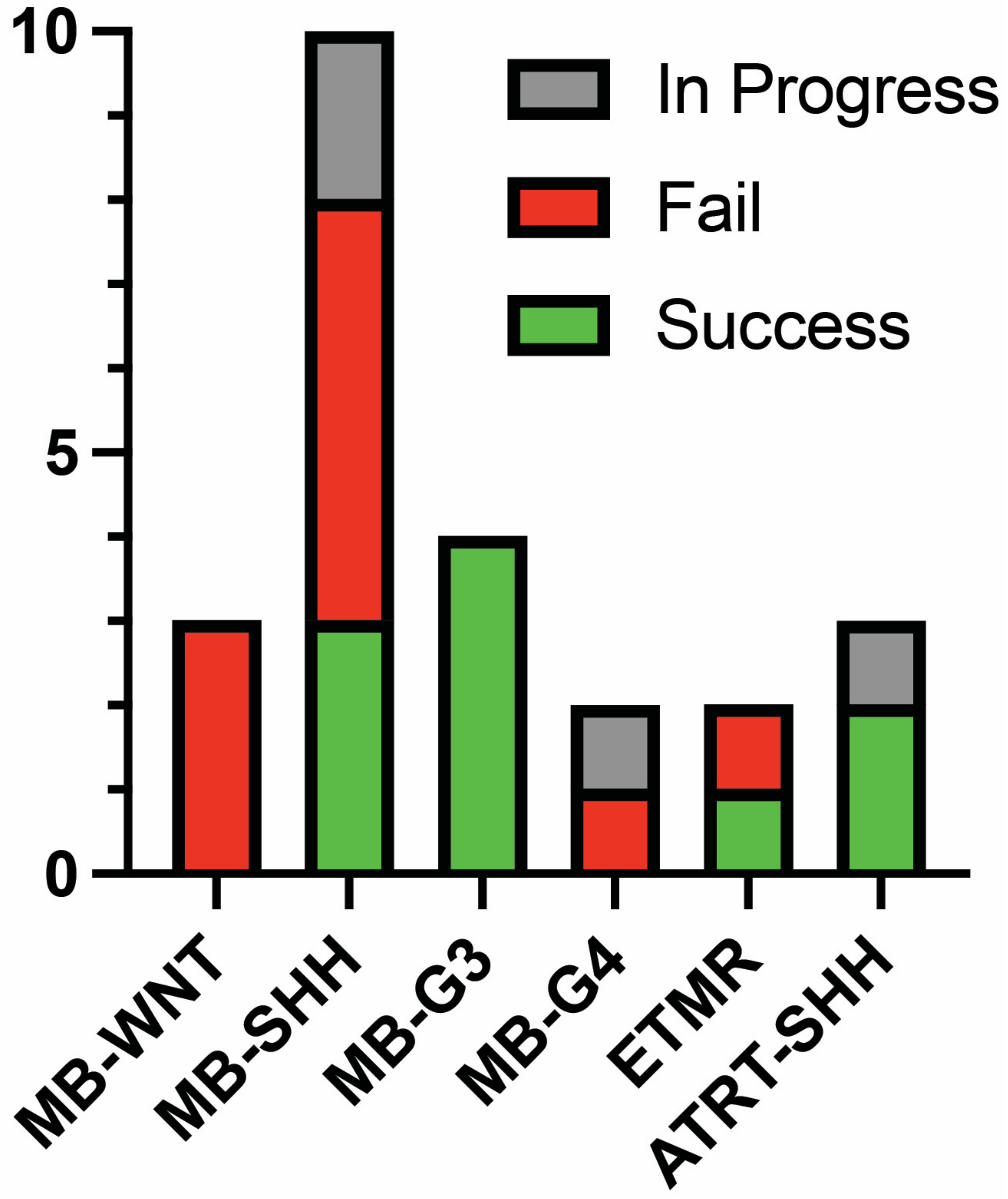


**Fig. S13. Tumor organoid establishment rate by tumor subtype.**
Bar graph depicting the number of successful, failed, or in progress tumor organoid models based on molecular classification of the parental PDOX.

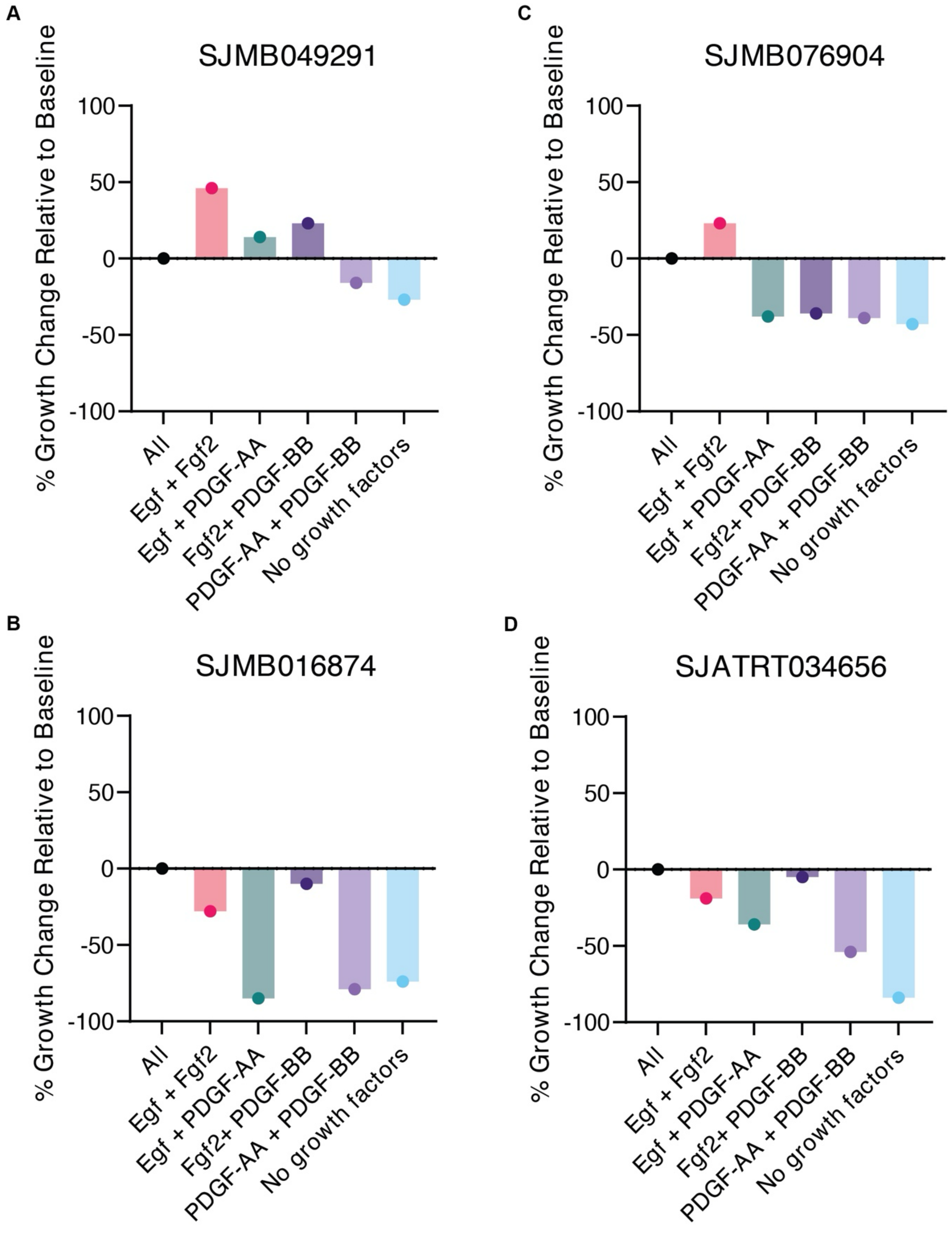


**Fig. S14. Growth factor dependencies of various tumor organoid types.**
Growth factor dependency assays show that Group 3 medulloblastoma (**A, B**) have optimal growth with only EGF and FGF2 whereas SHH medulloblastoma (**C**) and ATRT (**D**) both require all four growth factors for optimal culture conditions.

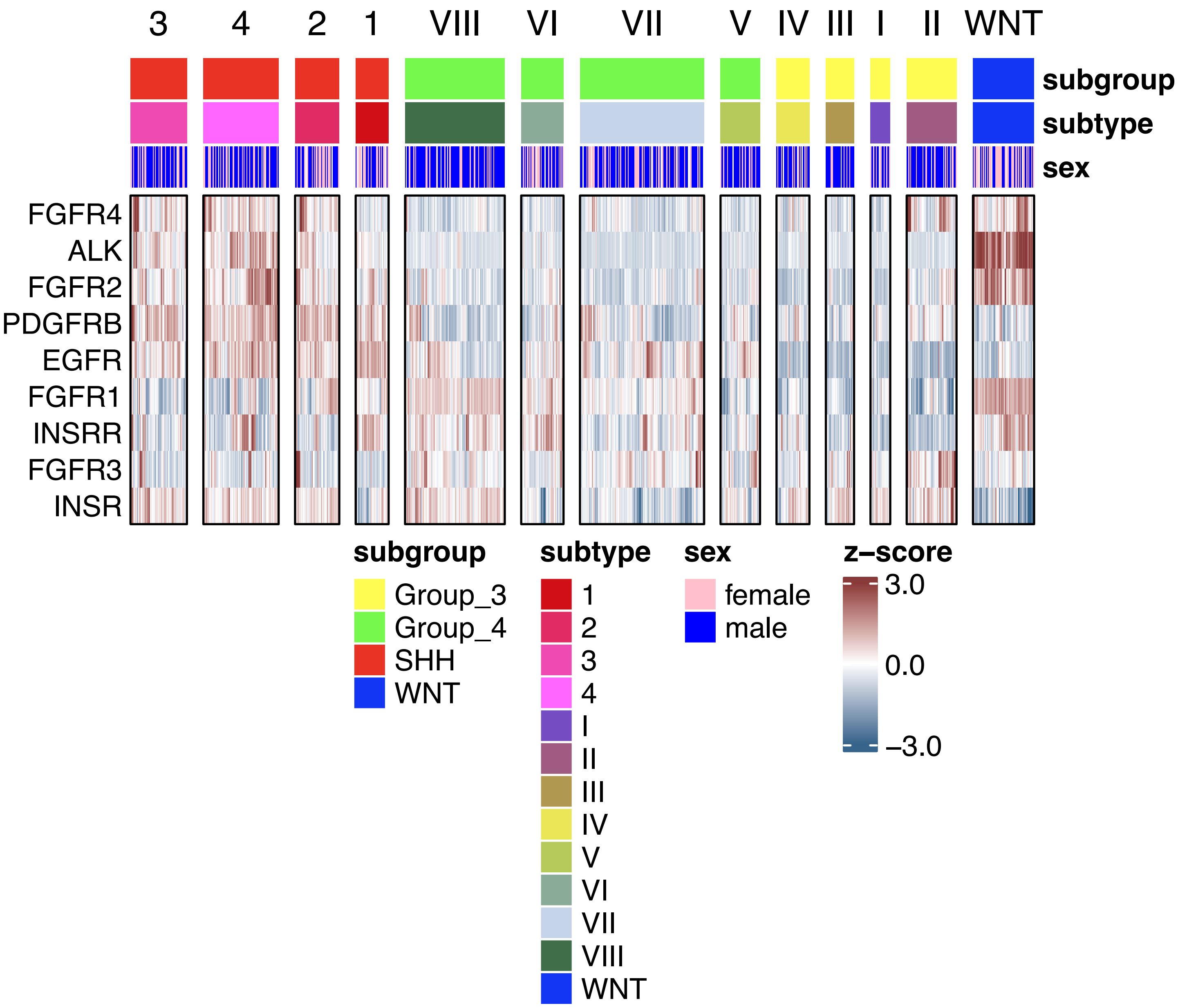


**Fig. S15. Expression of growth factor receptors in pediatric embryonal brain tumors.**
Graphical representation of growth factor receptor expression by normalized Z-score for SHH-MB and G3-MB PDOX models. Included growth factor receptors are those that have been tested in tumor organoid media formulations. These data show high expression of PDGFRB in the SHH-MB models, highlighting the need of this ligand in the media to promote tumor organoid growth. Moreover, these data show high expression of FGFR3 and FGFR4 in G3-MB subtype II PDOXs, highlighting the necessity for FGF2 to develop tumor organoids of this tumor subtype. G3-MB subtype II is characterized by *MYC* amplification, which is harbored in all established G3-MB tumor organoids.

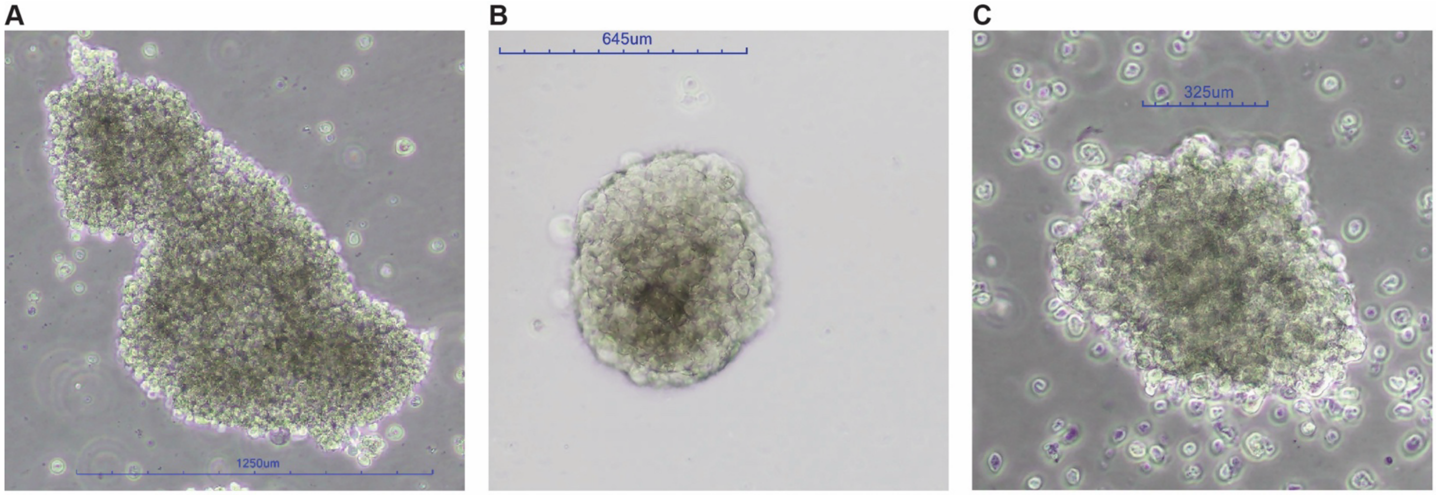


**Fig. S16. Tumor organoids ready for passaging.**
Representative wide-field microscopy images of tumor organoids ready to be passaged: (**A**) ATRT- SHH SJATRT096759, (**B**) MB-SHH SJMB016874, and (**C**) MB-G3 SJMB016800.

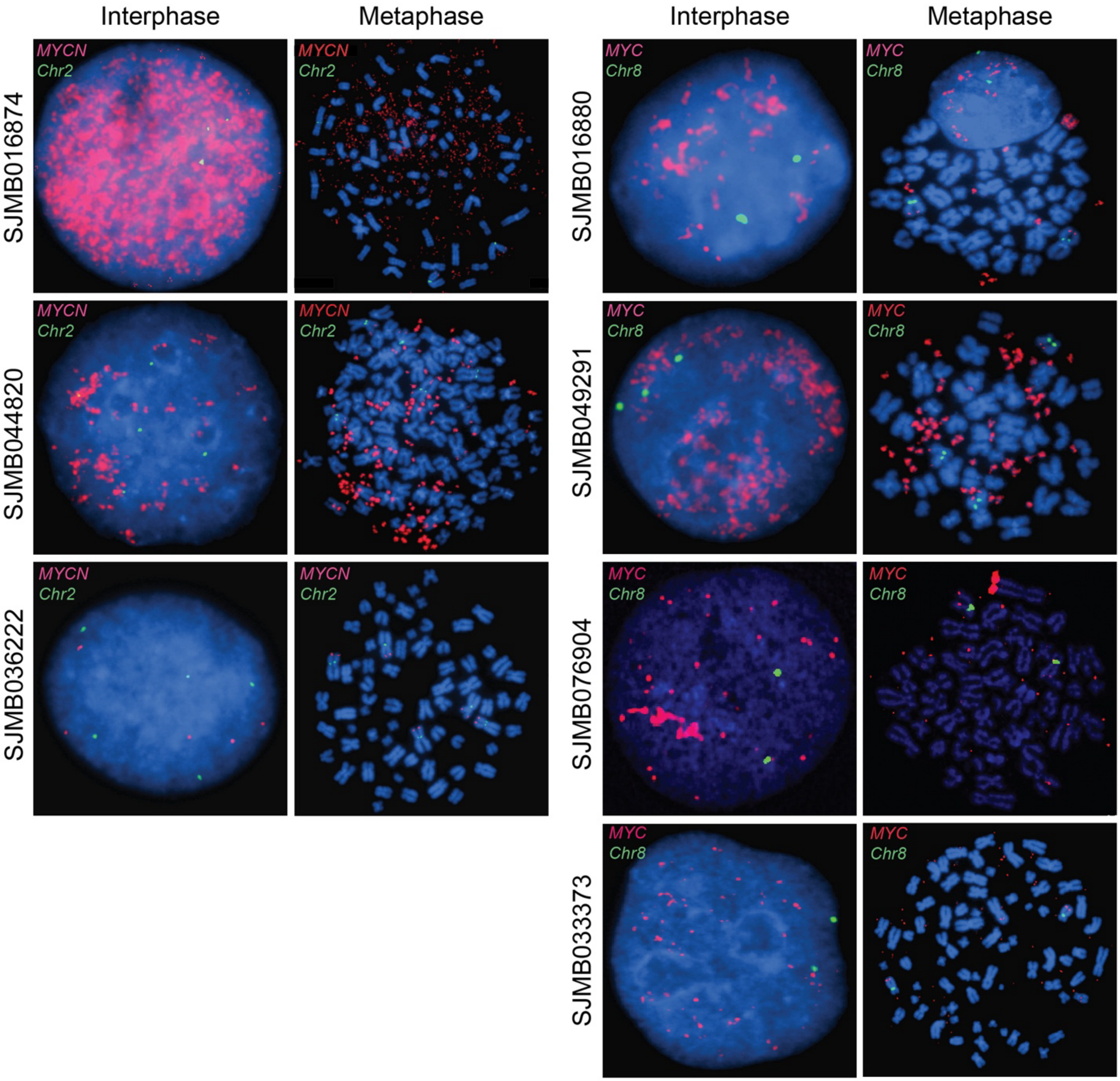


**Fig. S17. FISH imaging of established tumor organoid models.**
Representative interphase (left) and metaphase (right) showing *MYCN* or *MYC* amplification in the form of extrachromosomal DNA for the SHH-MB and G3-MB tumor organoid models, respectively.

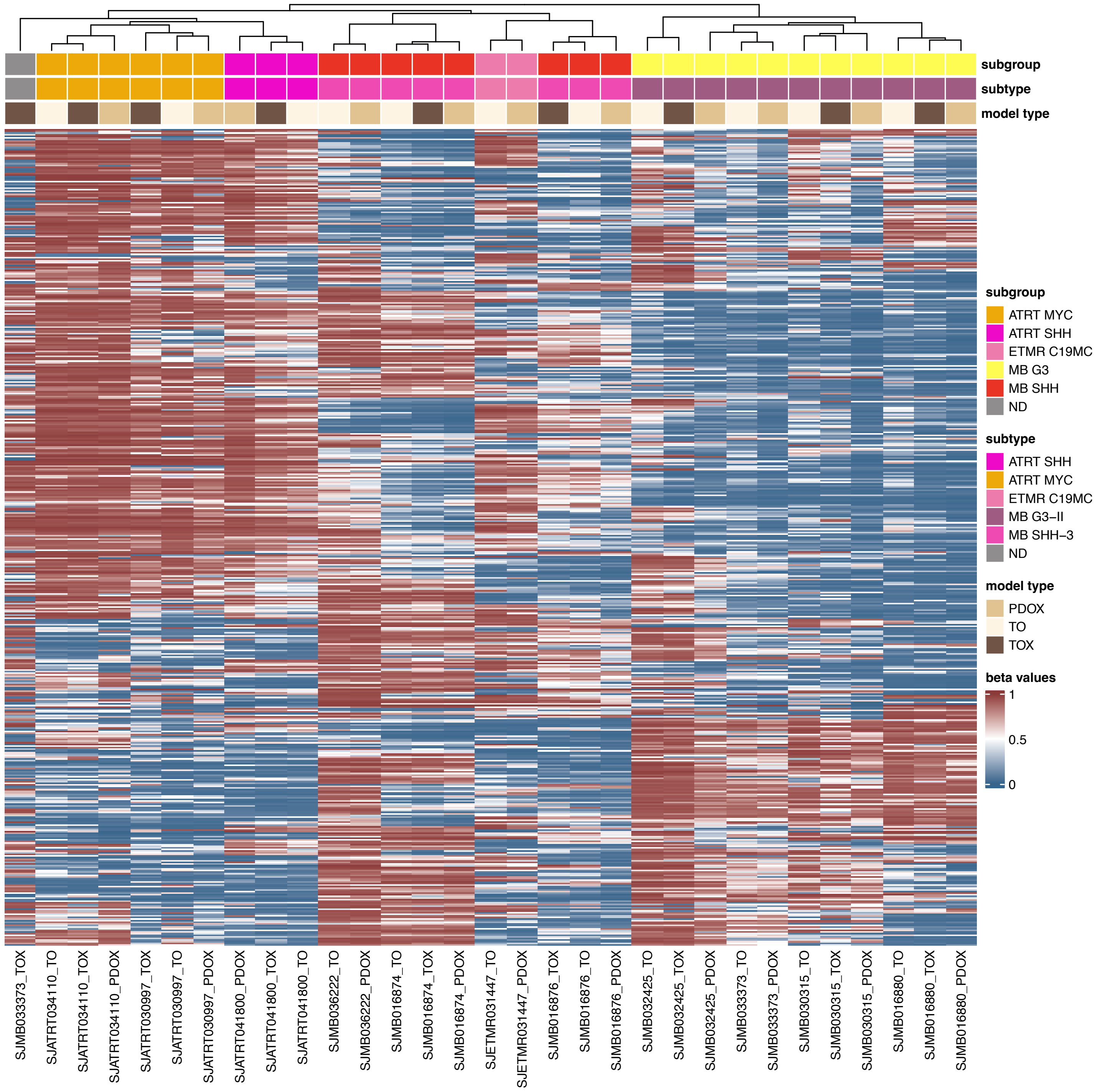


**Fig. S18. Methylation-based clustering of PDOX, TO, and TOX models.**
Unsupervised hierarchical clustering of 1000 most variably methylated probes by median absolute deviation.

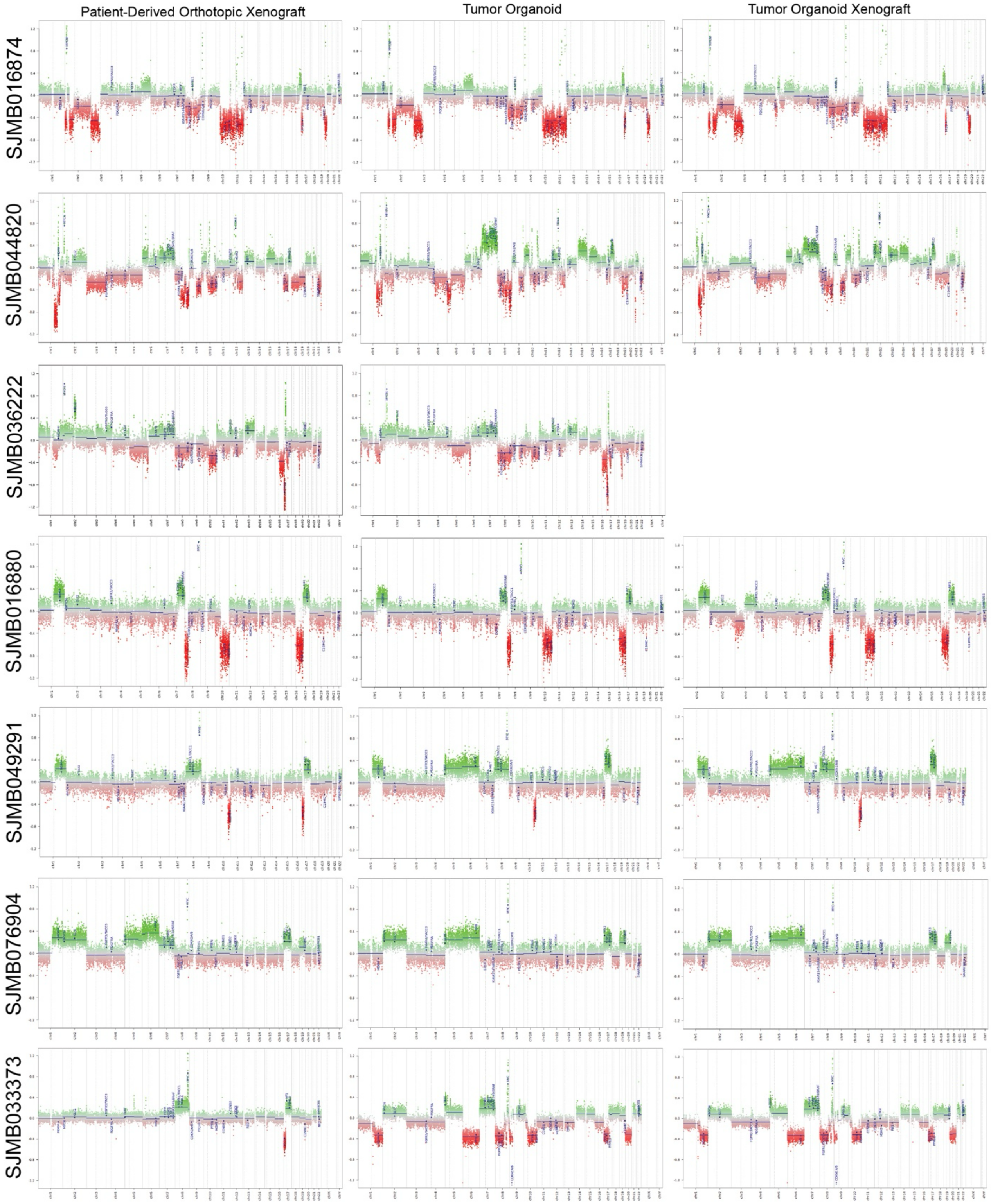


**Fig. S19. Methylation-based CNV plots of established PDOX, TO, and TOX models.**
Methylation-based copy number variation plots of all established tumor organoid models show conservation of genetic amplification and deletion events at all different developmental stages. SJMB033373 is the only model that undergoes a shift in culture. This has been replicated by three independent attempts by placing this model in culture, suggesting that this shift is due to an underlying subclone in the PDOX tumor rather than by random chance.

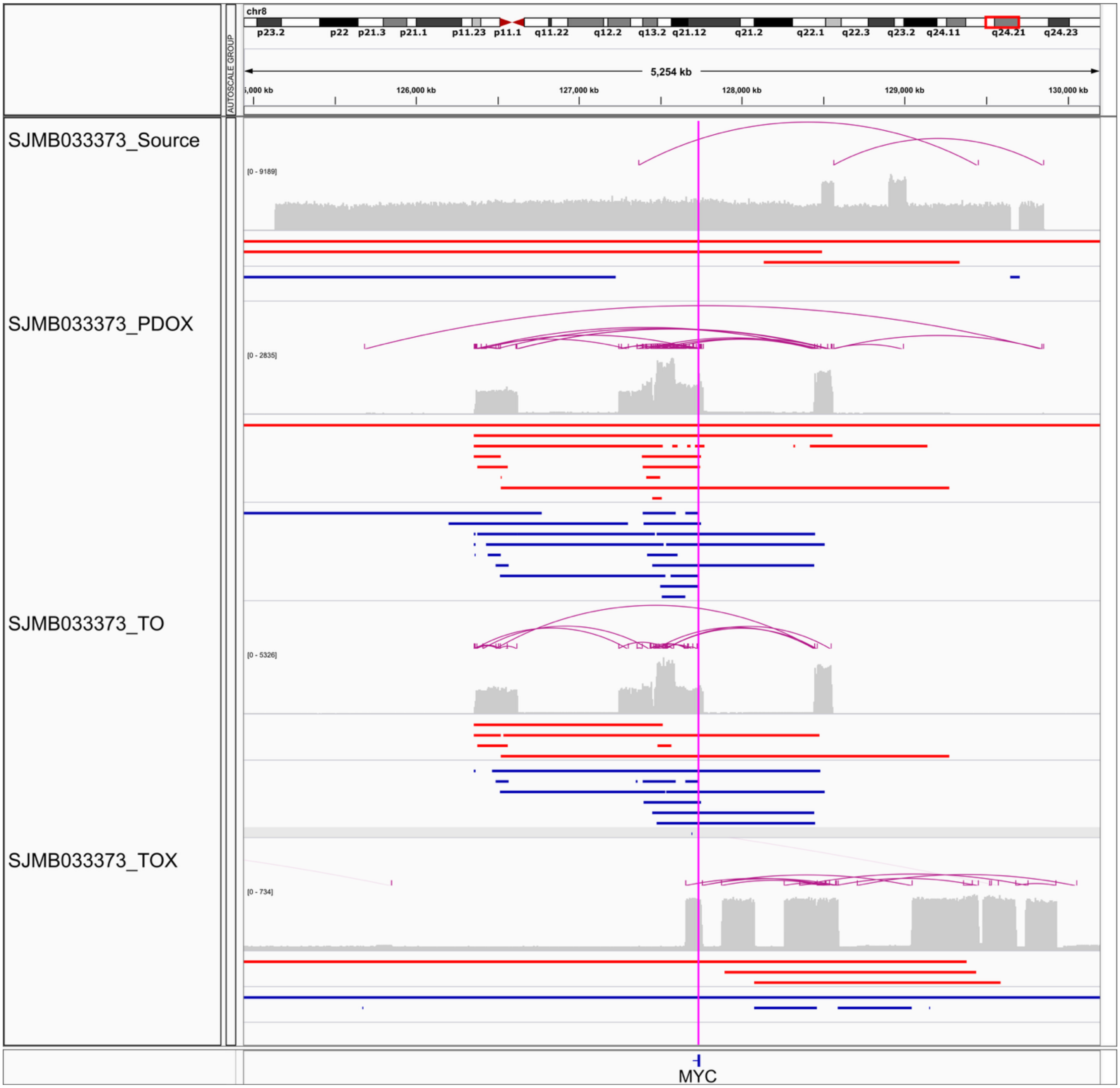


**Fig. S20. Variation of *MYC*-harboring structural variants in SJMB033373.**
SJMB033373 (MB G3-II) Source, PDOX, TO, and TOX model structural variant call visualization, indicating *MYC* amplification. Manta structural variant calls that passed default filters are presented, including inversions (purple arcs), tandem duplications (red bars), and deletions (blue bars). WGS coverage highlighting copy gain/loss is plotted in gray; *MYC* is annotated across samples by a magenta bar.

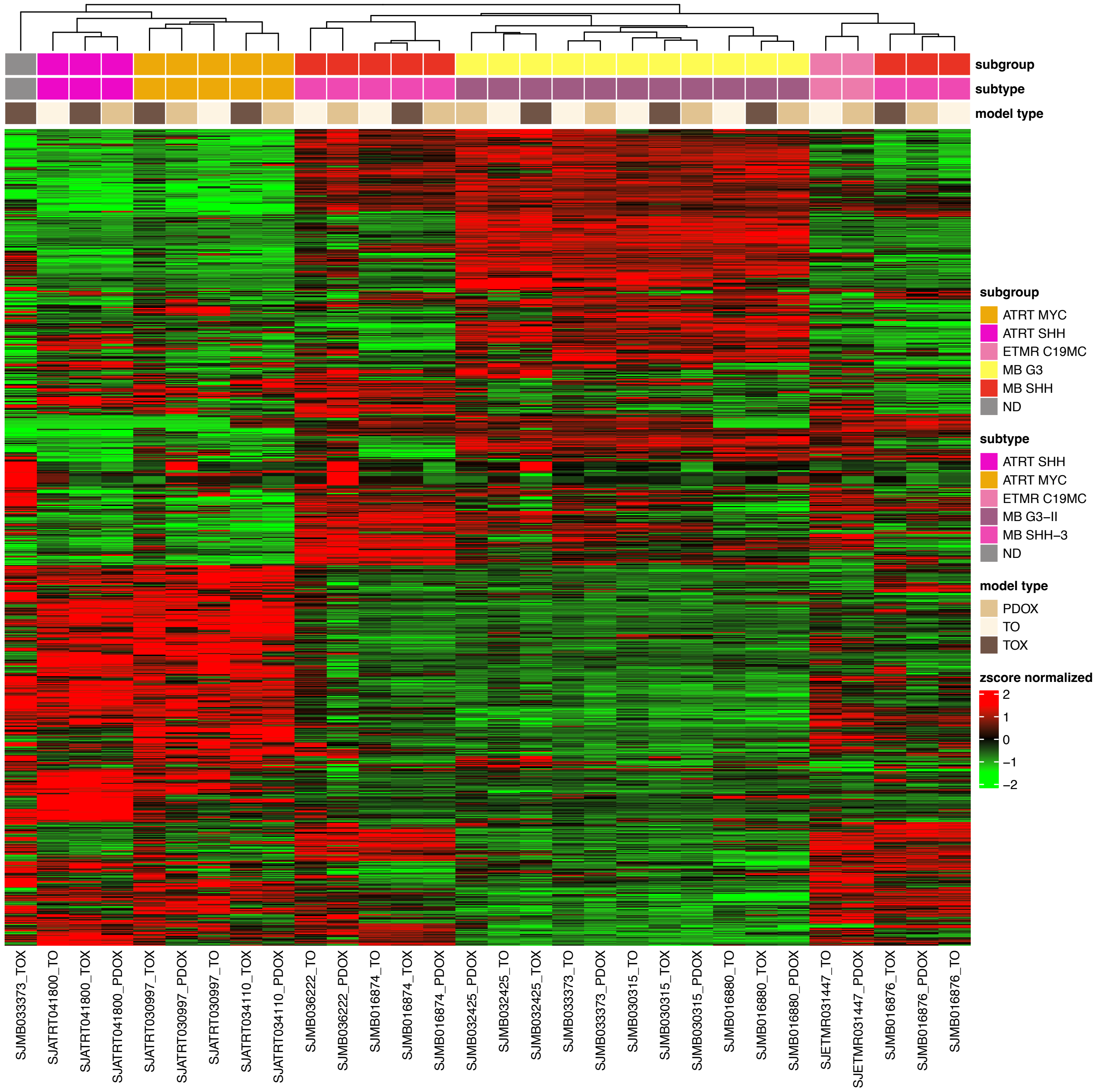


**Fig. S21. Transcriptomic clustering of PDOX, TO, and TOX models.**
Unsupervised hierarchical clustering of bulk RNA-seq profiles using the 1000 topmost variably expressed genes by median absolute deviation.

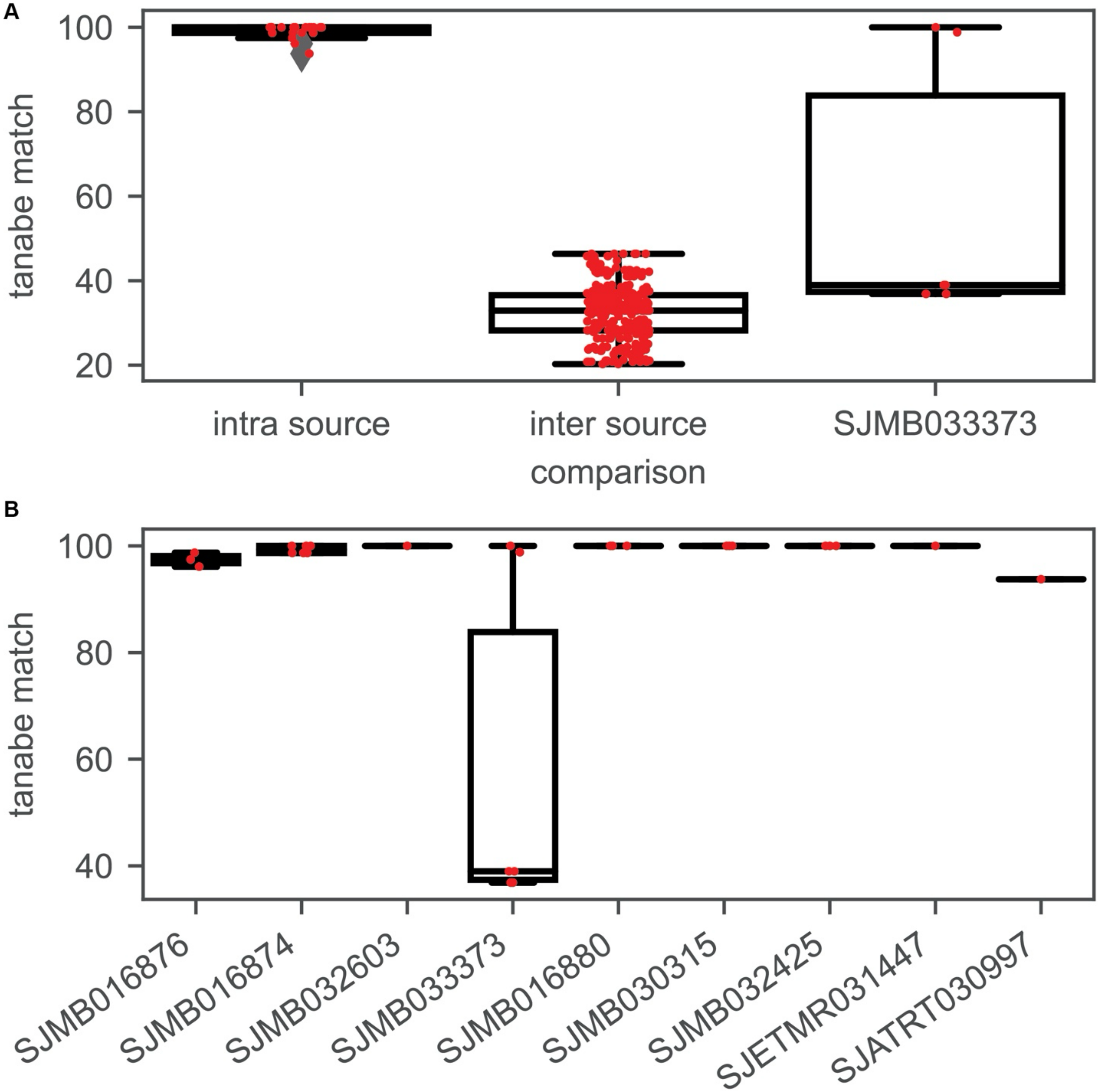


**Fig. S22. SJMB033373 PDOX, TO, and TOX short tandem repeat analysis.**
(**A**) Short tandem repeat (STR) profile similarity as calculated by the Tanabe match (0-100, 100 indicating a perfect match) was performed between models of the same source (intra-source, highest theoretical similarity), between independent models (inter-source, least similarity) as compared to pairwise comparisons of all SJMB033373 models (a sample specific intra-source comparison). (**B**) Individual sample intra-source STR profile comparisons.

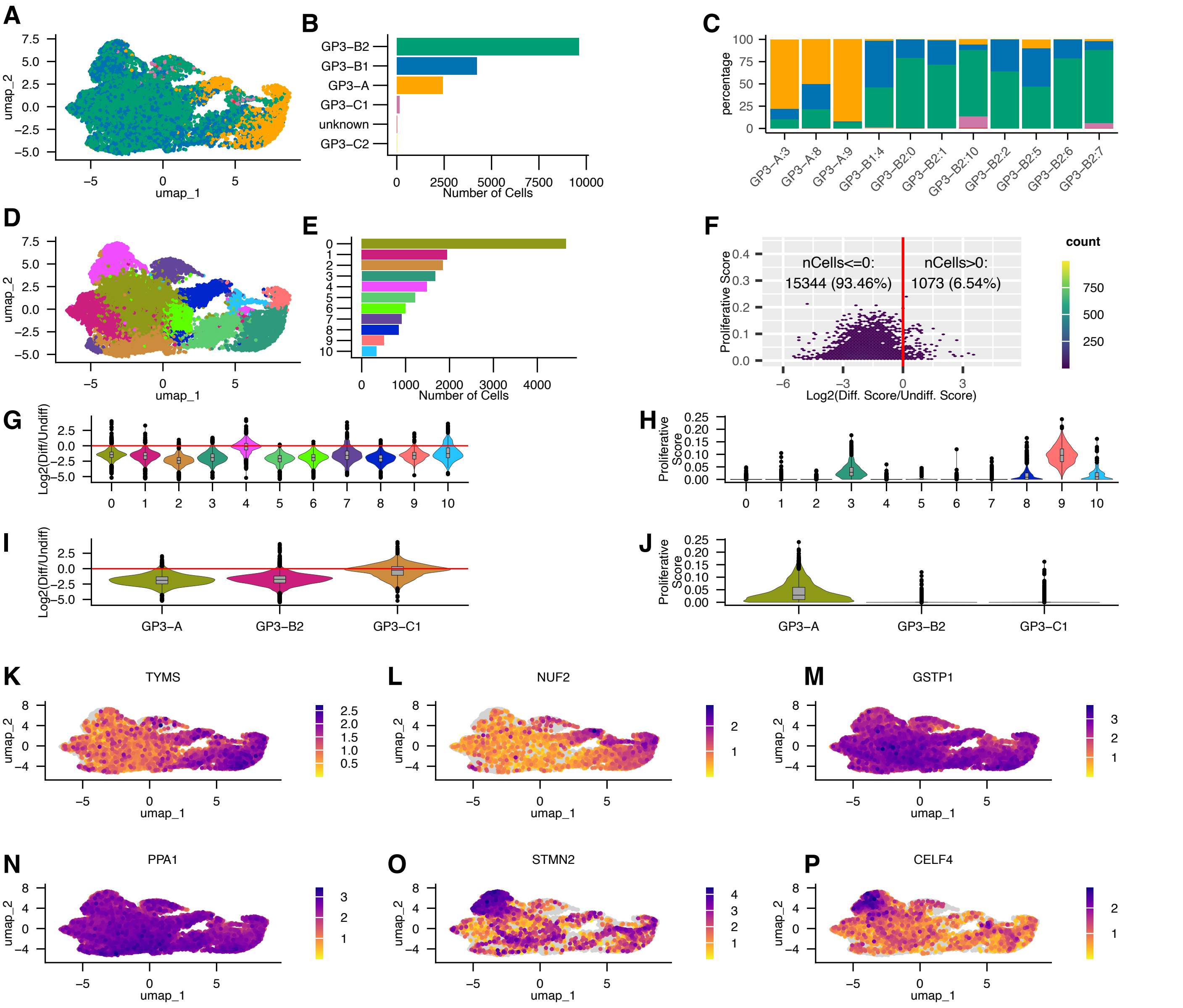

**Fig. S23. SJMB030315 (MB-G3) single-cell sequencing.**
(**A**) UMAP of integrated PDOX/TO/TOX dataset annotated by SingleR with MB-G3 malignant cell datasets. (**B**) The number of each SingleR annotated cell types as represented in (**A**). (**C**) Percentage of SingleR annotated cell types from (**A, B**) found in each identified subcluster. (**D**) UMAP plot of the integrated PDOX/TO/TOX dataset, with each cluster highlighted; the number of cells in each cluster are shown in (**E**). (**F**) The distribution of UCell scores with the y-axis indicating cell Proliferative scores, and the x-axis indicating the log2(differentiated UCell score + 0.01/undifferentiated UCell score + 0.01), highlighting the majority of cells are undifferentiated (negative x-axis) while maintaining a smaller group of differentiated cells (positive x-axis). (**G**) The distribution of the log2(Differentiated/Undifferentiated) scores (y-axis) for each cluster (x-axis). (**H**) the distribution of UCell proliferative scores (x-axis) for each cluster (y-axis). (**I**) the distribution of the log2(Differentiated/Undifferentiated) scores (y-axis) by annotated cell type on the x-axis. (**J**) the distribution of UCell proliferative scores (y-axis) for each annotated cell type, split by model (y-axis). (**K-P**) representative cell marker expression overlaying the integrated dataset UMAP plot for each cell type; (**K, L**)=proliferative, (**M, N**)=undifferentiated, (**O, P**)=neuron-like/differentiated.

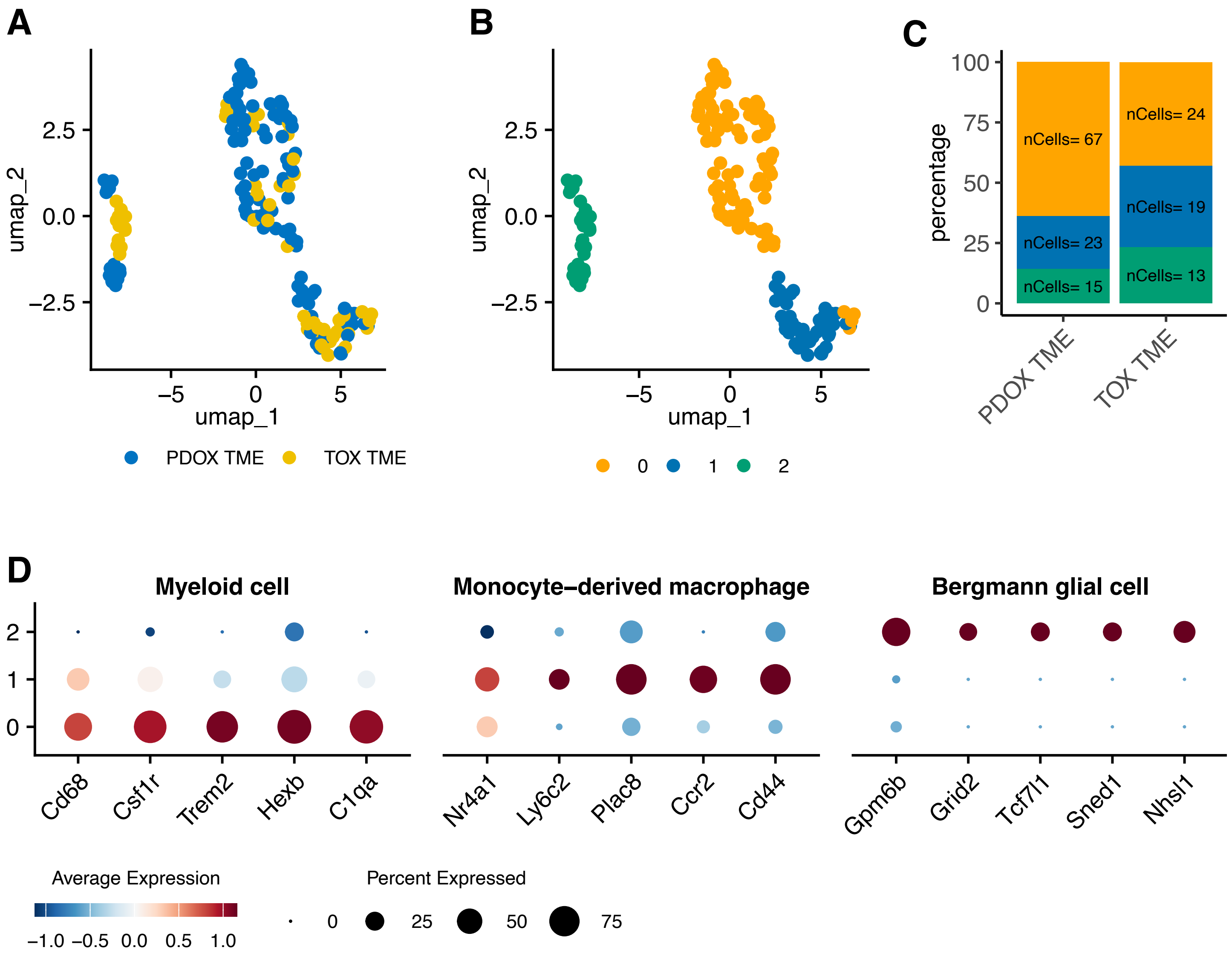


**Fig S24. Tumor microenvironment analysis of SJMB030315 (MB-G3) by single-cell sequencing.** (**A**) UMAP of combined mouse cell tumor microenvironment (TME) scRNA-seq datasets comprised of PDOX TME (blue) and TOX TME (yellow). (**B**) UMAP plot of the combined mouse cell dataset clusters annotated by SlimR/CellMarker2.0 and cell marker expression. (**C**) The percentage of cells identified in each subcluster (named 0, 1, 2), annotated as myeloid cells, monocyte-derived macrophages, and Bergmann glial cells, respectively. (**D**) Dotplot of cell type specific markers identified in each subcluster (padj < 0.05, percent expressed >20%, LogFC > 0.25).

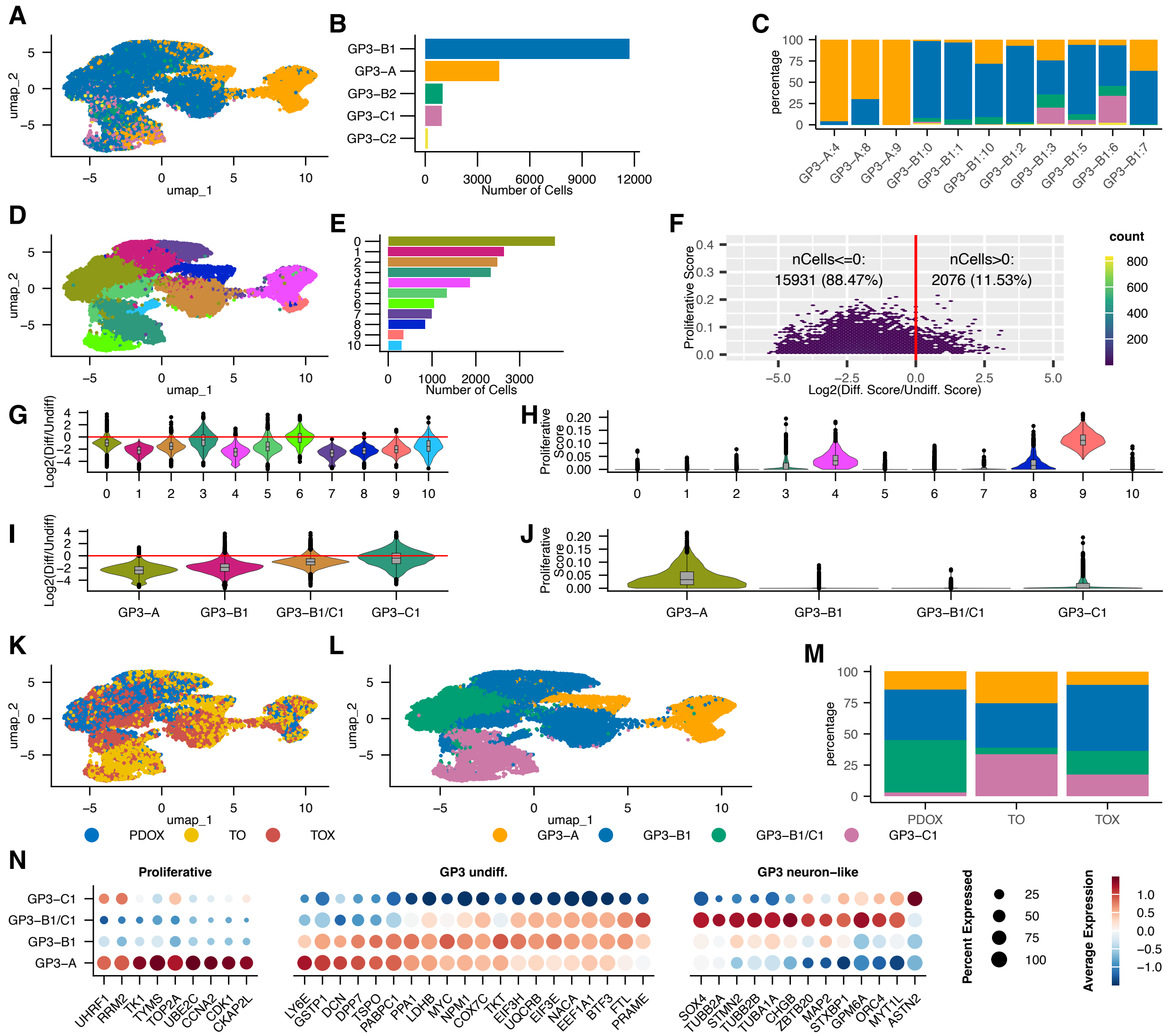

**Fig. S25. SJMB016880 (MB-G3) single-cell sequencing.**
(**A**) UMAP of integrated PDOX/TO/TOX dataset annotated by SingleR with MB-G3 malignant cell datasets. (**B**) The number of each SingleR annotated cell types as represented in (**A**). (**C**) Percentage of SingleR annotated cell types from (**A**, **B**) found in each identified subcluster. (**D**) UMAP plot of the integrated PDOX/TO/TOX dataset, with each cluster highlighted; the number of cells in each cluster are shown in (**E**). (**F**) The distribution of UCell scores with the y-axis indicating cell Proliferative scores, and the x-axis indicating the log2(differentiated UCell score + 0.01/undifferentiated UCell score + 0.01), highlighting the majority of cells are undifferentiated (negative x-axis) while maintaining a smaller group of differentiated cells (positive x-axis). (**G**) The distribution of the log2(Differentiated/Undifferentiated) scores (y-axis) for each cluster (x-axis). (**H**) the distribution of UCell proliferative scores (x-axis) for each cluster (y-axis). (**I**) the distribution of the log2(Differentiated/Undifferentiated) scores (y-axis) by annotated cell type on the x-axis. (**J**) the distribution of UCell proliferative scores (y-axis) for each annotated cell type, split by model (y-axis). (**K**) UMAP plot of the integrated single-cell RNA-seq dataset comprised of PDOX (blue), TO (yellow), and TOX (red) cells. (**L**) UMAP plot of the integrated dataset subclusters annotated by cell types previously reported in Group3 medulloblastoma. (**M**) Percentage of annotated cell types in each model from the integrated dataset. (**N**) Dotplot of key proliferative (GP3-A; orange), undifferentiated (GP3-B1; blue), mixed differentiated/undifferentiated (GP3-B/C; green) and differentiated (GP3-C; pink) cell markers differentially expressed (padj < 0.05, percent expressed > 50%, LogFC > 0.25) in annotated subclusters.

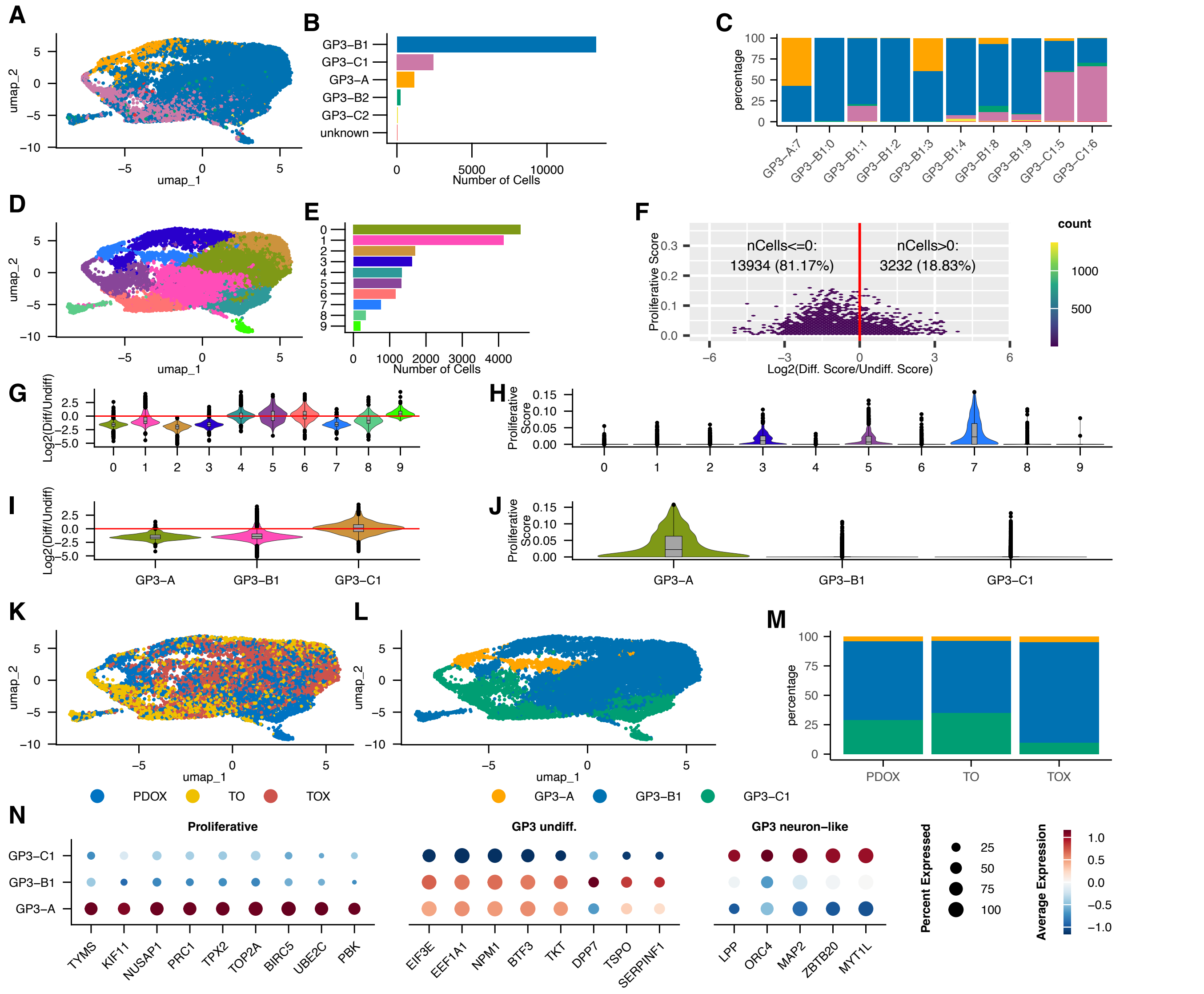

**Fig. S26. SJMB032425 (MB-G3) single-cell sequencing.**
(**A**) UMAP of integrated PDOX/TO/TOX dataset annotated by SingleR with MB-G3 malignant cell datasets. (**B**) The number of each SingleR annotated cell types as represented in (**A**). (**C**) Percentage of SingleR annotated cell types from (**A**, **B**) found in each identified subcluster. (**D**) UMAP plot of the integrated PDOX/TO/TOX dataset, with each cluster highlighted; the number of cells in each cluster are shown in (**E**). (**F**) The distribution of UCell scores with the y-axis indicating cell Proliferative scores, and the x-axis indicating the log2(differentiated UCell score +0.01/undifferentiated UCell score + 0.01), highlighting the majority of cells are undifferentiated (negative x-axis) while maintaining a smaller group of differentiated cells (positive x-axis). (**G**) The distribution of the log2(Differentiated/Undifferentiated) scores (y-axis) for each cluster (x-axis). (**H**) the distribution of UCell proliferative scores (x-axis) for each cluster (y-axis). (**I**) the distribution of the log2(Differentiated/Undifferentiated) scores (y-axis) by annotated cell type on the x-axis. (**J**) the distribution of UCell proliferative scores (y-axis) for each annotated cell type, split by model (y-axis). (**K**) UMAP plot of the integrated single-cell RNA-seq dataset comprised of PDOX (blue), TO (yellow), and TOX (red) cells. (**L**) UMAP plot of the integrated dataset subclusters annotated by cell types previously reported in Group3 medulloblastoma. (**M**) Percentage of annotated cell types in each model from the integrated dataset. (**N**) Dotplot of key proliferative (GP3-A; orange), undifferentiated (GP3-B1; blue), and differentiated (GP3-C; green) cell markers differentially expressed (padj< 0.05, percent expressed > 50%, LogFC > 0.25) in annotated subclusters.

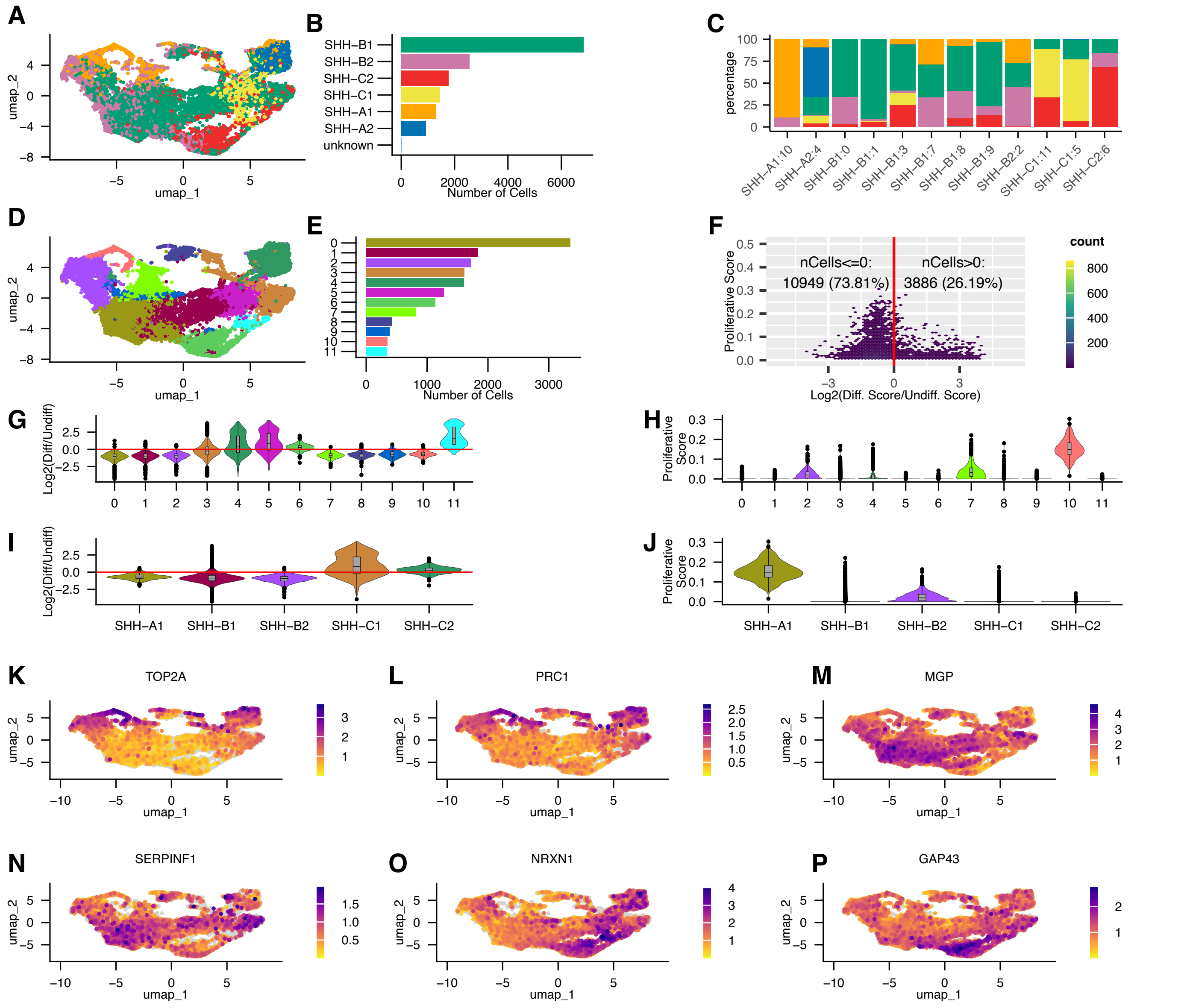


**Fig. S27. SJMB016874 (MB-SHH) single-cell sequencing.**

(**A**) UMAP of integrated PDOX/TO/TOX dataset annotated by SingleR with MB-SHH malignant cell datasets. (**B**) The number of each SingleR annotated cell types as represented in (**A**). (**C**) Percentage of SingleR annotated cell types from (**A**, **B**) found in each identified subcluster. (**D**) UMAP plot of the integrated PDOX/TO/TOX dataset, with each cluster highlighted; the number of cells in each cluster are shown in (**E**). (**F**) The distribution of UCell scores with the y-axis indicating each individual cell's Proliferative scores, and the x-axis indicating the log2(differentiated UCell score + 0.01/undifferentiated UCell score + 0.01), highlighting a larger subpopulation of undifferentiated cells (negative x-axis) while maintaining a smaller group of differentiated cells (positive x-axis). (**G**) The distribution of the log2 (Differentiated/Undifferentiated) scores (y-axis) for each cluster (x-axis). (**H**) The distribution of UCell proliferative scores (x-axis) for each cluster (y-axis). (**I**) The distribution of the log2(Differentiated/Undifferentiated) scores (y-axis) by annotated cell type and split by model (PDOX, green; TO, red; TOX, purple) on the x-axis. (**J**) The distribution of UCell proliferative scores (y-axis) for each annotated cell type, split by model (y-axis). (**K-P**) representative cell marker expression overlaying the integrated dataset UMAP plot for each cell type; (**K, L**)=proliferative, (**M, N**)=undifferentiated, (**O, P**)=neuron-like/differentiated.

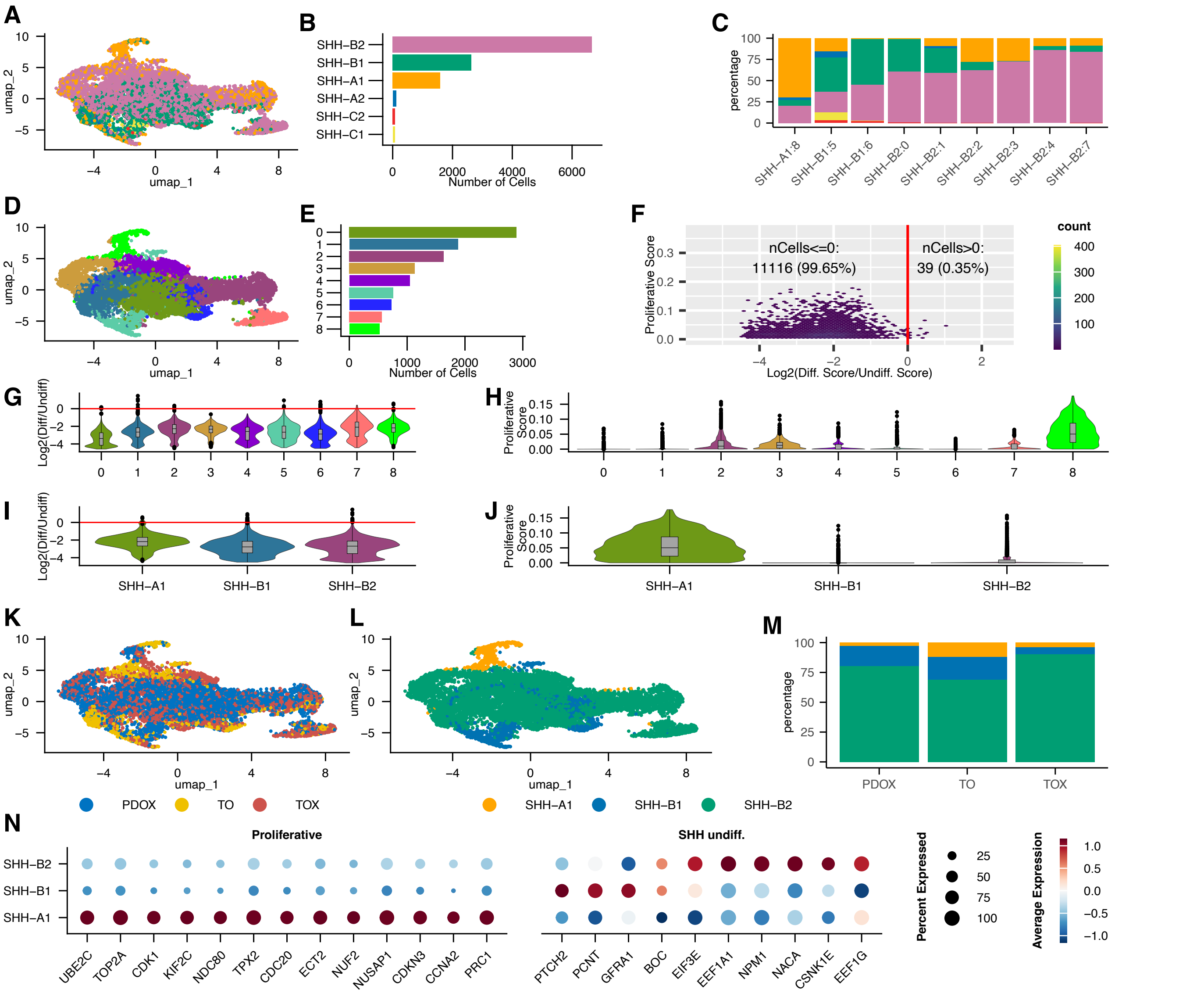


**Fig. S28. SJMB016876 (MB-SHH) single-cell sequencing.**
(**A**) UMAP of integrated PDOX/TO/TOX dataset annotated by SingleR with MB-SHH malignant cell datasets. (**B**) The number of each SingleR annotated cell types as represented in (**A**). (**C**) Percentage of SingleR annotated cell types from (**A**, **B**) found in each identified subcluster. (**D**) UMAP plot of the integrated PDOX/TO/TOX dataset, with each cluster highlighted; the number of cells in each cluster are shown in (**E**). (**F**) The distribution of UCell scores with the y-axis indicating cell Proliferative scores, and the x-axis indicating the log2(differentiated UCell score + 0.01/undifferentiated UCell score + 0.01), showing <1% of the cell population have a differentiated signature. (**G**) The distribution of the log2(Differentiated/Undifferentiated) scores (y-axis) for each cluster (x-axis). (**H**) The distribution of UCell proliferative scores (x-axis) for each cluster (y-axis). (**I**) The distribution of the log2(Differentiated/Undifferentiated) scores (y-axis) by annotated cell type on the x-axis. (**J**) The distribution of UCell proliferative scores (y-axis) for each annotated cell type, split by model (y-axis). (**K**) UMAP plot of the integrated single-cell RNA-seq dataset comprised of PDOX (blue), TO (yellow), and TOX (red) cells. (**L**) UMAP plot of the integrated dataset subclusters annotated by cell types previously reported in SHH medulloblastoma. (**M**) Percentage of annotated cell types in each model from the integrated dataset. (**N**) Dotplot of key proliferative (SHH-A1; orange) and undifferentiated (SHH-B1/2; blue/green) cell markers differentially expressed (padj < 0.05, percent expressed > 50%, LogFC > 0.25) in annotated subclusters.

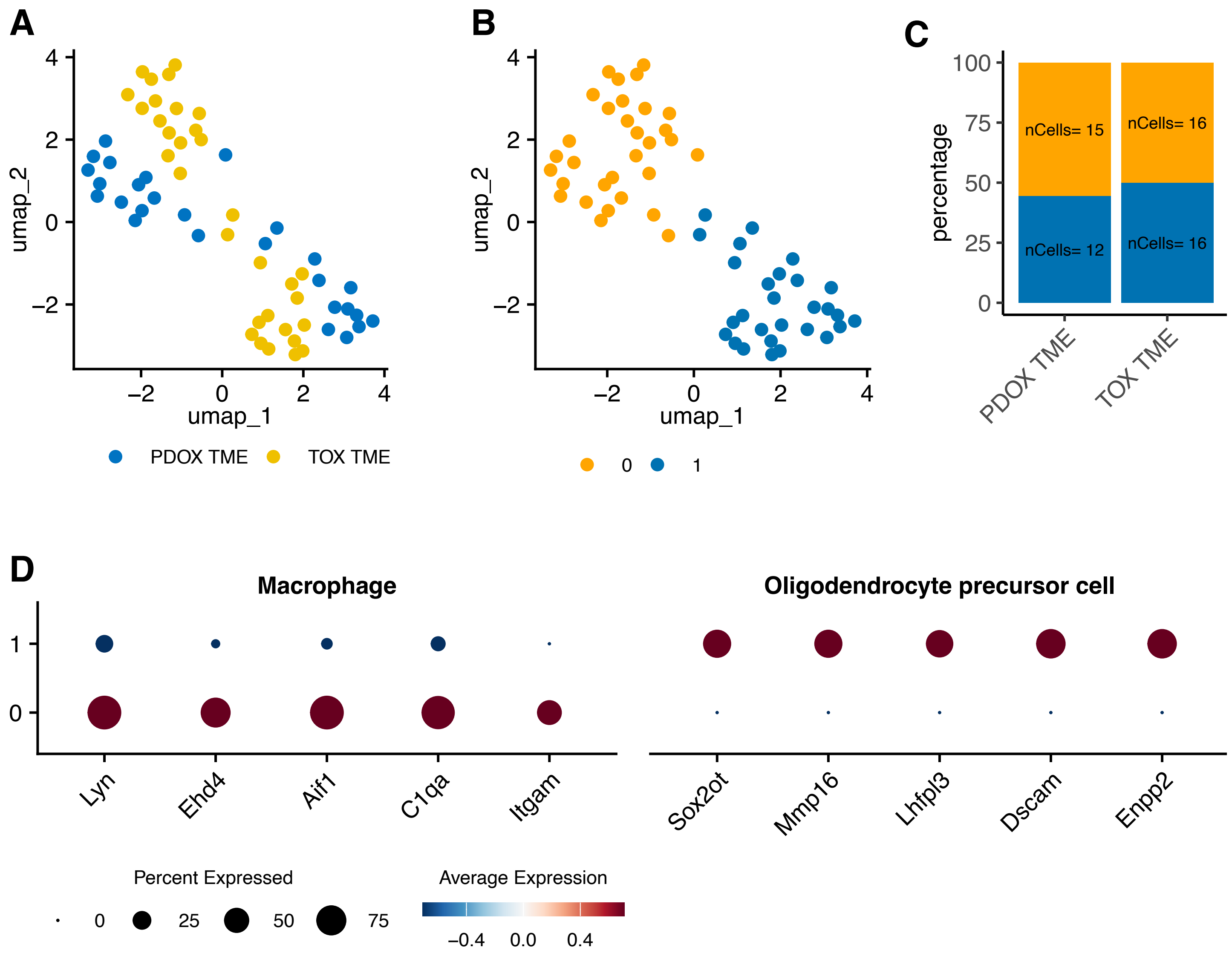


**Fig. S29. Tumor microenvironment analysis of SJMB016876 (MB-SHH) by single-cell sequencing.** (**A**) UMAP of combined mouse cell tumor microenvironment (TME) scRNA-seq datasets comprised of PDOX TME (blue) and TOX TME (yellow). (**B**) UMAP plot of the combined mouse cell dataset clusters annotated by SlimR/CellMarker2.0 and cell marker expression. (**C**) The percentage of cells identified in each subcluster (named 0 and 1), annotated as macrophage and oligodendrocyte precursor cells, respectively. (**D**) Dotplot of cell type specific markers identified in each subcluster (padj < 0.05, percent expressed >20%, LogFC > 0.25).

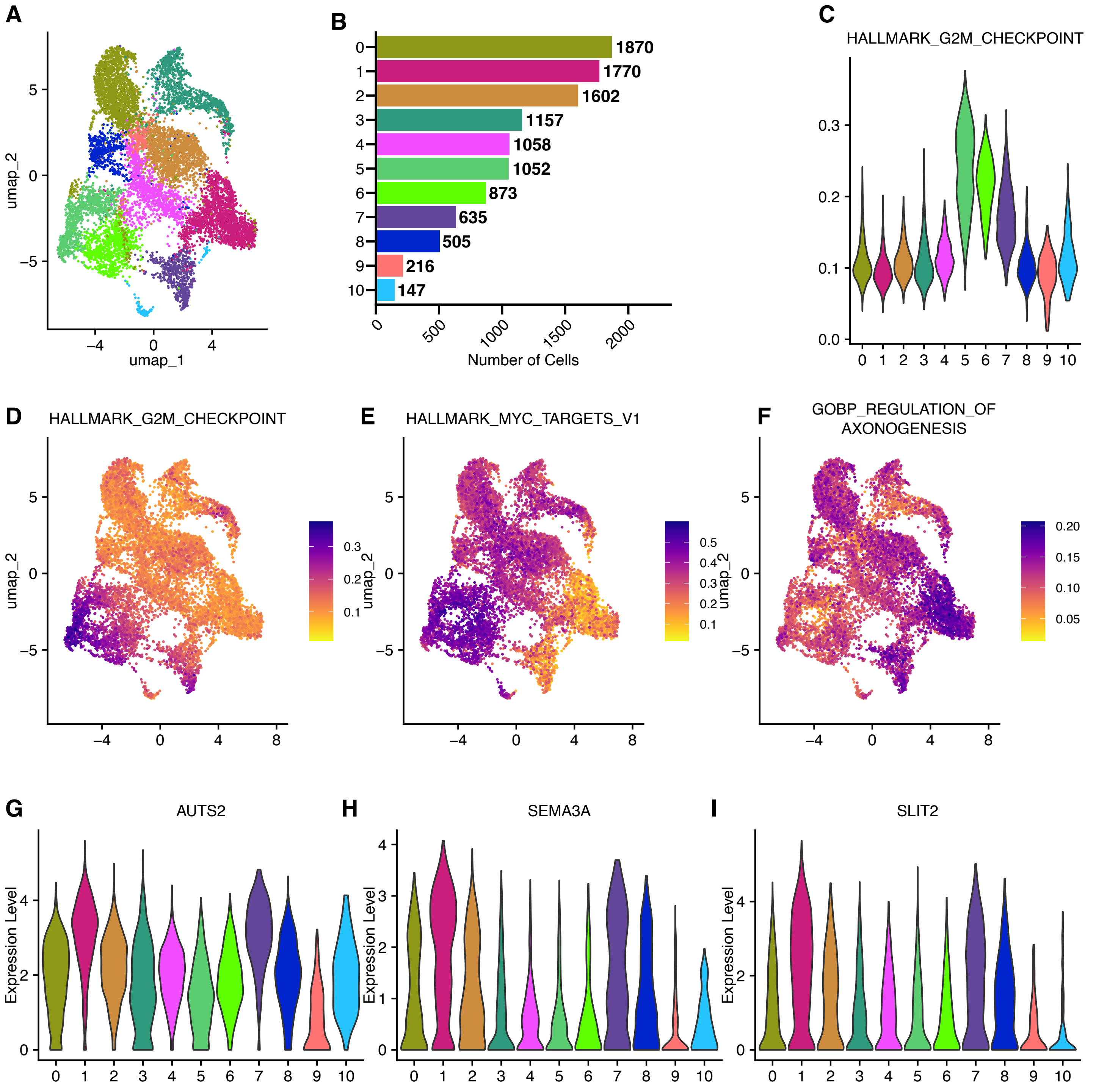


**Fig. S30. SJATRT041800 (ATRT-SHH) single-cell sequencing.**
(**A**) UMAP plot of the integrated PDOX/TO/TOX dataset, with each cluster highlighted; the number of cells in each cluster is shown in (**B**). (**C**) The distribution of HALLMARK_G2M_CHECKPOINT UCell scores (y-axis) in each cluster (x-axis). (**D-F**) UMAP plot of the integrated datasets with UCell scores for HALLMARK_G2M_CHECKPOINT (**D**), HALLMARK_MYC_TARGETS_V1 (**E**) and GOBP_REGULATIONOF_AXONOGENESIS (**F**). (**G-I**) Distribution of single cell RNA-seq expression (y-axis) of representative differentially expressed axonogenesis genes in each cluster (x-axis).

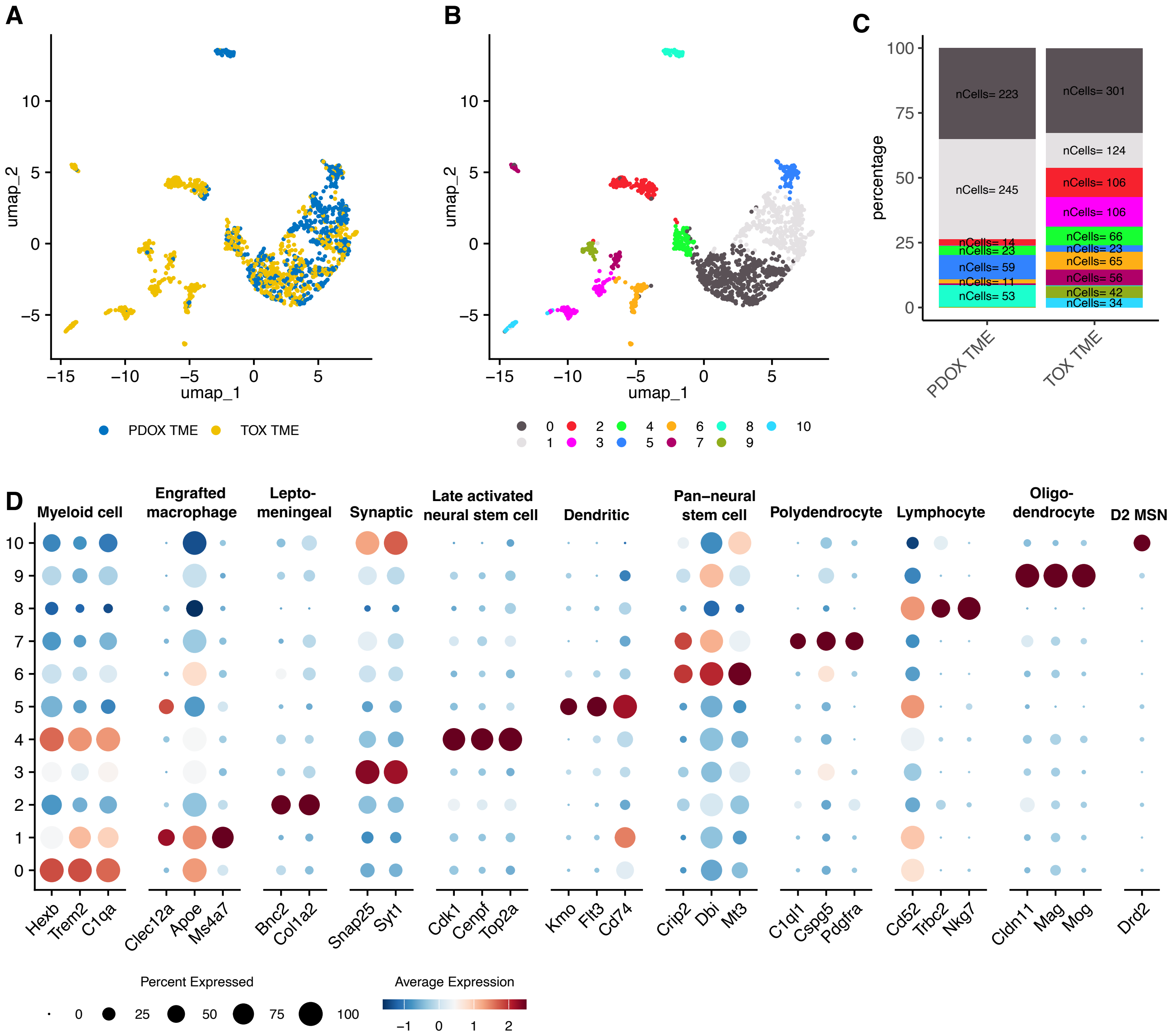


**Fig. S31. Tumor microenvironment analysis of SJMB041800 (ATRT-SHH) by single-cell sequencing.** (**A**) UMAP of combined mouse cell tumor microenvironment (TME) scRNA-seq datasets comprised of PDOX TME (blue) and TOX TME (yellow). (**B**) UMAP plot of the combined mouse cell dataset clusters annotated by SlimR/CellMarker2.0 and cell marker expression. (**C**) The percentage of cells identified in each subcluster (named 0-9), annotated as myeloid, macrophage, leptomeningeal, synaptic, late activate neural stem cell, dendritic, pan-neural stem cell, polydendrocyte, lymphocyte, oligodendrocyte, and D2 medium spiny neuron (D2 MSN), respectively. Subcluster 9 (D2 MSN) PDOX TME cells were not annotated. Stacked bars representing clusters with ≥ 5 cells are labeled with number of cells. (**D**) Dotplot of cell type specific markers identified in each subcluster/annotated cell type (padj < 0.05, percent expressed >20%, LogFC > 0.25).

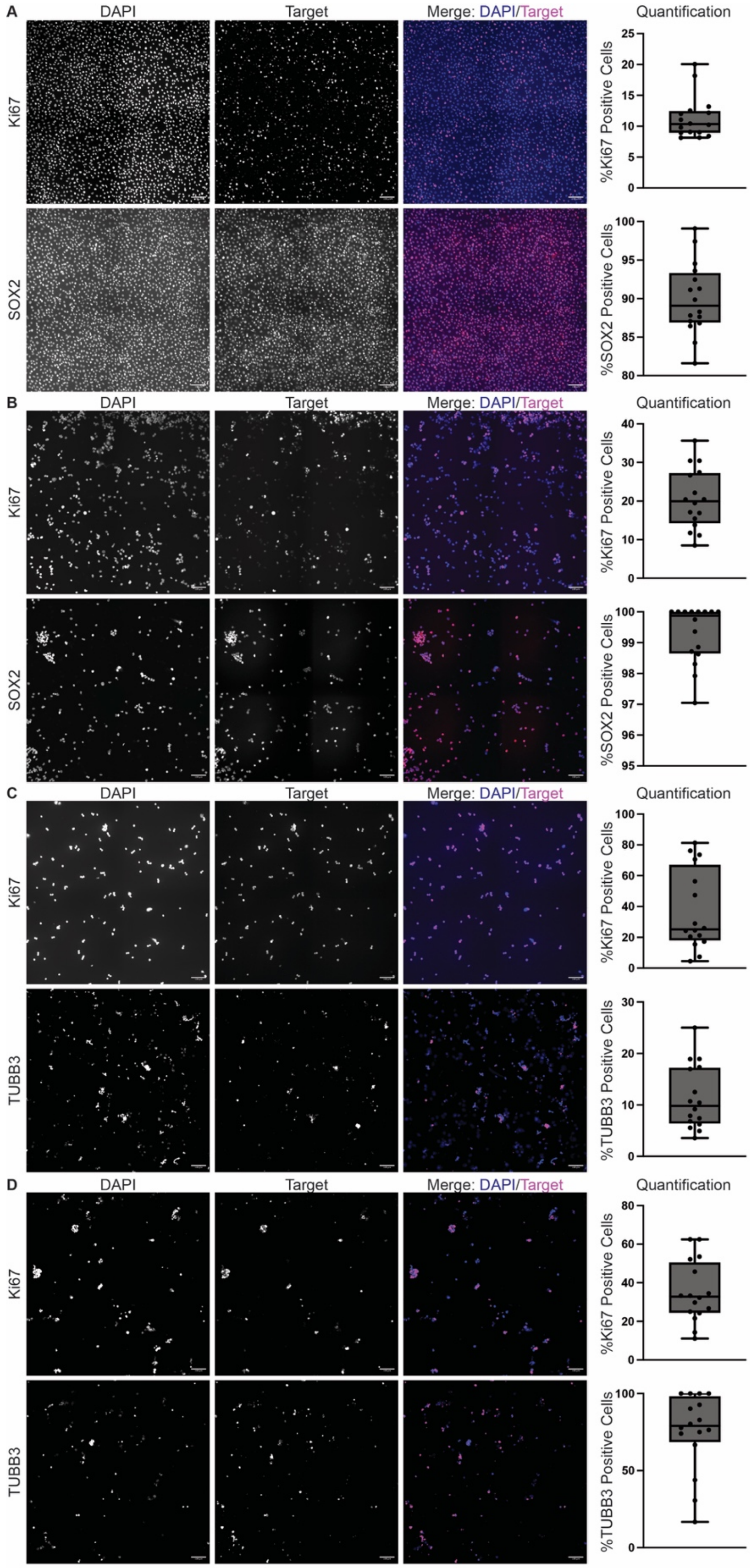


**Fig. S32. Immunofluorescent imaging of tumor organoids.**

Immunofluorescent imaging and quantification of tumor organoids for Ki67 or SOX2 in (**A**) ATRT-SHH SJATRT041800 and (**B)** MB-SHH SJMB016874; Ki67 or TUBB3 in MB-G3 (**C**) SJMB016880 and (**D**) SJMB030315.

**Table S1. Summary of all samples analyzed and discussed.**
Excel file matching all sample IDs with ComBioID with additional data, including which cohort the sample came from, the original patient tumor classification, methylation-based classification, and the sequencing methods performed.

**Table S2. Supporting information for called SNV/InDels.**
Excel file containing supporting information from consensus variant callers for called SNVs and InDels.

**Table S3. Supporting information for called CNVs/SVs.**
Excel file containing supporting information from CONSERTING and Manta v1.5 for called CNVs and SVs.

**Table S4. Supporting information for called RNA fusions.**
Excel file containing supporting information from STAR-fusion for called RNA fusions.

| Tumor Organoid Model | Growth Factor Combination | Growth Normalized to Media Composition 1 |
|---|---|---|
| SJMB030315 | Egf + Fgf2 + PDGF-AA + PDGF-BB | Baseline |
| SJMB030315 | Egf + Fgf2 | 46% increase |
| SJMB030315 | Egf + PDGF-AA | 14% increase |
| SJMB030315 | Fgf+ PDGF-BB | 23% increase |
| SJMB030315 | PDGF-AA + PDGF-BB | 16% decrease |
| SJMB030315 | No growth factors | 27% decrease |
| | | |
| SJMB030425 | Egf + Fgf2 + PDGF-AA + PDGF-BB | Baseline |
| SJMB030425 | Egf + Fgf2 | 23% increase |
| SJMB030425 | Egf + PDGF-AA | 38% decrease |
| SJMB030425 | Fgf+ PDGF-BB | 36% decrease |
| SJMB030425 | PDGF-AA + PDGF-BB | 39% decrease |
| SJMB030425 | No growth factors | 43% decrease |
| | | |
| SJATRT034656 | Egf + Fgf2 + PDGF-AA + PDGF-BB | Baseline |
| SJATRT034656 | Egf + Fgf2 | 19% decrease |
| SJATRT034656 | Egf + PDGF-AA | 36% decrease |
| SJATRT034656 | Fgf+ PDGF-BB | 5% decrease |
| SJATRT034656 | PDGF-AA + PDGF-BB | 54% decrease |
| SJATRT034656 | No growth factors | 84% decrease |
| | | |
| SJMB016876 | Egf + Fgf2 + PDGF-AA + PDGF-BB | Baseline |
| SJMB016876 | Egf + Fgf2 | 28% decrease |
| SJMB016876 | Egf + PDGF-AA | 85% decrease |
| SJMB016876 | Fgf+ PDGF-BB | 10% decrease |
| SJMB016876 | PDGF-AA + PDGF-BB | 79% decrease |
| SJMB016876 | No growth factors | 74% decrease |

**Table S5. Quantifications for growth factor dependency assays.**
Quantifications for the tumor organoid growth factor dependency assay presented in **Supplementary Fig. S10**.

**Table S6. STR analysis for established tumor organoid models.**
Excel file containing all STR sequencing results from each established tumor organoid and (if applicable) tumor organoid xenograft compared to the ancestral PDOX, highlighting that all but one model (SJMB033373) does not display genetic fingerprint drift after establishment as an *in vitro* model.

**Table S7. Raw data and statistical analysis of IF staining for tumor organoids.**
Excel file containing raw counts for each model and each staining target corresponding to **Figure 7** and **Supplementary Figure S32**. Also included are the relevant statistical values for similarity between PDOX and TOX cell counts.

**Table S8. Raw data and statistical analysis of PDOX and tumor organoid high-throughput screening.** Excel file containing the raw $EC_{50}$ values for each sample, drug, and replicate used in the high-throughput screening assay. Also included are the relevant statistical values used to calculate response similarity between the PDOX and tumor organoid samples.

| Component | Final Concentration | TSM | Efab |
|---|---|---|---|
| DMEM/F-12 HEPES | | ● | |
| Neurobasal-A | | ● | |
| KnockOut™ DMEM/F-12 | | | ● |
| Neurobasal® Medium | | | ● |
| StemPro® Neural Supplement | 10% | | ● |
| HEPES | 10mM | ● | |
| Glutamax | 1X | ● | ● |
| Sodium Pyruvate | 1X | ● | ● |
| Non-Essential Amino Acids | 1X | ● | ● |
| Penicillin/Streptomycin | 1X | ● | ● |
| B27 (-Vitamin A) | 1X | ● | ● |
| N2 Supplement | 1X | ● | ● |
| Heparin | 5 IU/ml | ● | ● |
| hu rPDGF-AA | 10 ng/ml | ● | ● |
| hu rPDGF-BB | 10 ng/ml | ● | ● |
| hu rEGF | 20 ng/ml | ● | ● |
| hu FPF-2 (bFgf) | 20 ng/ml | ● | ● |
| hu rInsulin, zinc solution | 2.5 ug/ml | ● | ● |
| L-glutamine | 2mM | ● | |

**Table S9. Tumor organoid culture conditions.**
Media formulations for TSM full and Efab media used to establish tumor organoids.

| Probe | Clone | Locus |
|---|---|---|
| *MYCN* probe 1 | RP11-355H10 | 2p24.1 |
| *MYCN* probe 2 | RP11-348M12 | 2p24.1 |
| *MYCN* control 1 | RP11-296A19 | 2q35 |
| *MYCN* control 2 | RP11-38408 | 2q35 |
| *MYC* probe 1 | CTD-3056022 | 8q24 |
| *MYC* probe 2 | CTD-2267H22 | 8q24 |
| *MYC* control 1 | RP11-1077A8 | 8p11.21 |
| *MYC* control 2 | RP11-867P15 | 8p11.21 |

**Table S10. Primary patient FISH probes.**
List of probes used for primary patient tumor FISH analysis, including BAC clone number and the targeted genomic locus.
